# Plasma Metabolomic and Lipidomic Alterations Associated with COVID-19

**DOI:** 10.1093/nsr/nwaa086

**Published:** 2020-04-28

**Authors:** Di Wu, Ting Shu, Xiaobo Yang, Jian-Xin Song, Mingliang Zhang, Chengye Yao, Wen Liu, Muhan Huang, Yuan Yu, Qingyu Yang, Tingju Zhu, Jiqian Xu, Jingfang Mu, Yaxin Wang, Hong Wang, Tang Tang, Yujie Ren, Yongran Wu, Shu-Hai Lin, Yang Qiu, Ding-Yu Zhang, You Shang, Xi Zhou

**Affiliations:** 1 Joint Laboratory of Infectious Diseases and Health, Wuhan Institute of Virology & Wuhan Jinyintan Hospital, Wuhan Institute of Virology, Center for Biosafety Mega-Science, Chinese Academy of Sciences (CAS), Wuhan, Hubei 430023 China; 2 State Key Laboratory of Virology, Wuhan Institute of Virology, Center for Biosafety Mega-Science, CAS, Wuhan, Hubei 430071, China; 3 Center for Translational Medicine, Jinyintan Hospital, Wuhan, Hubei 430023 China; 4 Joint Laboratory of Infectious Diseases and Health, Wuhan Institute of Virology & Wuhan Jinyintan Hospital, Wuhan Jinyintan Hospital, Wuhan, Hubei 430023 China; 5 Department of Critical Care Medicine, Union Hospital, Tongji Medical College, Huazhong University of Science and Technology, Wuhan, Hubei 430030 China; 6 Department of Infectious Diseases, Tongji Hospital, Tongji Medical College, Huazhong University of Science and Technology, Wuhan, Hubei 430030 China; 7 Wuhan Metware Biotechnology Co., Ltd, Wuhan, Hubei 430075 China; 8 Department of Neurology, Union Hospital, Tongji Medical College, Huazhong University of Science and Technology, Wuhan, Hubei 430022 China; 9 State Key Laboratory of Cellular Stress Biology, Innovation Center for Cell Signaling Network, School of Life Sciences, Xiamen University, Xiamen, Fujian 361102 China; 10 University of Chinese Academy of Sciences, Beijing 100049 China

## Abstract

The pandemic of the coronavirus disease 2019 (COVID-19) has become a global public health crisis. The symptoms of COVID-19 range from mild to severe conditions. However, the physiological changes associated with COVID-19 are barely understood. In this study, we performed targeted metabolomic and lipidomic analyses of plasma from a cohort of COVID-19 patients who had experienced different symptoms. We found the metabolite and lipid alterations exhibit apparent correlation with the course of disease in these COVID-19 patients, indicating that the development of COVID-19 affected whole-body metabolism of the patients. In particular, malic acid of the TCA cycle and carbamoyl phosphate of urea cycle reveal the altered energy metabolism and hepatic dysfunction, respectively. It should be noted that carbamoyl phosphate is profoundly down-regulated in fatal patients compared with mild patients. And more importantly, guanosine monophosphate (GMP), which is mediated by not only GMP synthase but also CD39 and CD73, is significantly changed between healthy subjects and COVID-19 patients, as well as between the mild and fatal groups. In addition, the dyslipidaemia was observed in COVID-19 patients. Overall, the disturbed metabolic patterns have been found to align with the progress and severity of COVID-19. This work provides valuable knowledge about plasma biomarkers associated with COVID-19 and potential therapeutic targets, as well as important resource for further studies of COVID-19 pathogenesis.

## Introduction

The outbreak of COVID-19, caused by severe acute respiratory syndrome coronavirus 2 (SARS-CoV-2), has been declared a pandemic by the World Health Organization (WHO). Up to the date of April 21, 2020, there are around 2.4 million confirmed COVID-19 cases and 162,956 deaths worldwide according to the situation report of WHO. Based on a recent study of 44,672 confirmed COVID-19 cases up to February 11 by Chinese Center for Disease Control and Prevention, over 19% COVID-19 patients developed severe or critical conditions (1). The global fatality rate is around 4.8% in all the confirmed cases until March 31, and has even reached 10% in some developed countries probably due to a more elderly population (2).

The main attacking organ of SARS-CoV-2 is low respiratory tract, and some patients develop life-threatening acute respiratory distress syndrome (ARDS). Besides, the attacks of liver, muscle, gastrointestinal tract, lymph node, and heart by SARS-CoV-2 have also been found or proposed (3-6). On the other hand, although more than 80% COVID-19 patients experienced only mild symptoms, it has been found that the conditions can rapidly progress from mild to severe ones, particularly in the absence of adequate medical care. Moreover, the mortality rate of COVID-19 in critically ill cases can be over 60%, posing great pressure on treatment (7). However, the physiological changes associated with COVID-19 under different symptomatic conditions are barely understood.

Metabolites and lipids are major molecular constituents in human plasma. During critical illness, metabolic and lipid abnormalities are commonly observed, which are believed to contribute to physiology and pathology. Moreover, previous studies have demonstrated dramatic alterations of metabolome and lipidome in human plasma caused by various diseases including viral infections, like Ebola virus disease (8, 9). Here, we performed the targeted metabolomic and lipidomic profiles of plasma samples collected from a cohort of COVID-19 patients, including COVID-19 fatalities and survivors recovered from mild or severe symptoms. Our findings here show many of the metabolite and lipid alterations, particularly ones associated with hepatic functions, align with the progress and severity of the disease, which would provide valuable knowledge about plasma biomarkers associated with COVID-19 as well as potential therapeutic targets, and shed light on the pathogenesis of COVID-19.

## Results

### Study design and patients

Blood samples were harvested at Wuhan Jinyintan Hospital from COVID-19 patients who were confirmed by laboratory nucleic acid test of SARS-CoV-2 infection. Serial samples were collected over the course of disease from 9 patients with fatal (F) outcome (F1-F4), 11 patients diagnosed as severe (S) symptoms (S1-S2), and 14 patients diagnosed as mild (M) symptoms (M1-M2) (Table S1). Of note, all the patients in the severe (S) and mild (M) groups had survived from COVID-19 and been discharged from the hospital. F1 represents the first samples collected from the COVID-19 fatal patients, while F4 represents the last samples before additional samples could be collected. S1 or M1 represents the samples during the disease peak of the patients in the severe or mild group as being determined based on the Diagnosis and Treatment Protocol for Novel Coronavirus Pneumonia (6th edition) published by the National Health Commission of China (10), while S2 or M2 represents the last samples collected from patients in each group before the patients were discharged from the hospital. For comparison, the blood samples from 10 healthy volunteers, whose throat swabs and serological testing were negative for SARS-CoV-2, were collected. The hydrophilic and hydrophobic metabolites were extracted from each plasma sample, respectively and measured by employing liquid chromatography electrospray ionization tandem mass spectrometry (LC-ESI-MS/MS) system. The metabolite identification was conducted by home-made database with retention time and ion pairs. For those metabolites without authentic standards in our database, we still used MS/MS spectra to search against the public databases for improving the confidence of metabolite identification. The orthogonal partial least-squares discriminant analysis (OPLS-DA) was used to discriminate metabolomic profiles between the groups of COVID-19 patients and healthy people (Figure S1-S4). In total, 431 metabolites and 698 lipids were identified and quantified, and both metabolome and lipidome showed dramatic alterations in the plasma of these COVID-19 patients (Table S2 and S3).

**Plasma metabolomic alternations associated with clinical symptoms of COVID-19**

For different courses of fatal COVID-19 patients (F1-F4), we analyzed the metabolites that underwent significant change [F4 vs. H, >1 $\log_2$ fold change (FC) <-1, typically $P$ <0.05]. For F vs. H, 87 of the total 431 metabolites were significantly different ($P$ < 0.05) at F1, while the number of significantly altered metabolites increased to 162 at F4 in the fatalities; and most of the changes are down-regulated (Table S2). We found a positive correlation between the alteration of metabolites and the course of disease deterioration in fatal patients (Figure 1A and Table S4), indicating that the development of disease affects the metabolism of metabolites.

We also profiled the metabolites in the different courses of severe and mild COVID-19 patients (S1 and S2; M1 and M2), and analyzed those that underwent the significant change [S1 vs. H, >1 $\log_2$ FC <-1, typically $P$ <0.05; M1 vs. H, >1 $\log_2$ FC <-1, typically $P$ <0.05] (Figure 1B and Table S5). There are apparently less metabolites with significant changes (>1 $\log_2$ FC <-1, typically $P$ <0.05) observed in severe and mild patient groups when compared with those of fatal patients, and almost all the significantly altered metabolites were down-regulated. These results indicate that the alterations of metabolic pathways were more extensive in fatal COVID-19 cases than in severe and mild ones who finally survived.

In addition, it is noteworthy that although the patients in both severe and mild groups had met the hospital discharge criteria in the time points S2 and M2 as their COVID-19 nucleic acid tests which turn out to be negative twice consecutively, our metabolomic data clearly show that many of their metabolites had not returned to normal levels when compared with those in healthy volunteers (Figure 1B), suggesting that these discharged patients had not been fully recovered from the impacts of COVID-19 in physiology.

To further analyze the metabolomic data, the differentiating metabolites were divided into those shared by all groups (F vs. H, S vs. H, and M vs. H) or those unique to the fatal group (F vs. H). Then, we performed Kyoto Encyclopedia of Genes and Genomes (KEGG) functional enrichment analysis to annotate the potential functional implication of differentiating metabolites among these groups (Figure 2). As shared by all the three symptomatic groups, differentiating metabolites enriched in total 12 pathways and significantly enriched in 3 pathways including pyrimidine metabolism, fructose and mannose metabolism, and carbon metabolism (Figure 2A-B and Table S6).

On the other hand, in the case of the fatality group, differentiating metabolites significantly enriched in 4 pathways, including thyroid hormone synthesis, thyroid hormone signaling, purine metabolism, and autoimmune thyroid (Figure 2C-D and Table S7), suggesting that the alterations in these pathways are associated with the progress and deterioration of COVID-19.

A prominent signature observed among fatal COVID-19 patients was an acute reduction of metabolites in patient plasma with the aggravation of the course of COVID-19. By comparing healthy subjects and fatal patients, we highlighted top 5 differentiating metabolites (Figure 3). For instance, malic acid, an intermediate of the tricarboxylic acid (TCA) cycle, exhibited the greatest $\log_2$ FC (-5.2) among all significantly altered metabolites in the fatalities. Similarly, aspartic acid shows markedly down-regulated in the plasma of patients (Table S4 and Table S5). Thereby, we postulate that a deficiency of malic acid and aspartate due, at least in part, to SARS-CoV-2 replication by hijacking nucleic acids from host cells, because the TCA cycle and aspartate would be preferent for purine and pyrimide nucleotide biosynthesis. One more differentiating metabolite guanosine monophosphate (GMP) is also observed, which involved the metabolic reactions mediated by not only GMP synthase but also CD39 and CD73. Both CD39 and CD73 are the immunomodulatory enzymes, suggesting that CD39/CD73 axis imbalance may occur in COVID-19 patients. In addition, we observed that the level of carbamoyl phosphate was significantly and gradually reduced over the course of COVID-19 fatalities. Carbamoyl phosphate is synthesized from free amino donors by carbamoyl phosphate synthetase I (CPSI) in mitochondria of liver cells, and participates in the urea cycle to remove excess ammonia and produce urea (14-17). Its reduction in fatal cases of COVID-19 suggests the possibility of liver damage, which could also impair amino acid and pyrimidine metabolisms since CPSI could also maintain pyrimidine pool. Notably, both GMP and carbamoyl phosphate show significant changes between fatal and mild patients, indicating that the disease progression is associated with immune dysfunction and nucleotide metabolism.

**Plasma lipidomic alternations correspond to clinical symptoms of COVID-19**

We analyzed the lipids in different courses of fatal COVID-19 patients (F1-F4) that underwent significant change [F4 vs. H, >1 $\log_2$ FC <-1, typically $P$ <0.05]. Most of the significantly changed lipids are up-regulated and a positive correlation between the alteration of lipids and the course of disease deterioration could be readily observed in the fatal patients (Figure 4A and Table S8). Lipid subclasses including diglycerides (DGs), free fatty acids (FAAs), and triglycerides (TGs), were identified in higher abundance in the fatality group (F vs. H), and the relative abundances of these lipids increased with the deterioration of the disease. Particularly, DG(16:0/20:2/0:0) exhibited the greatest $\log_2$ FC (+4.15) in DGs, and TG(14:0/22:1/22:3) exhibited the greatest $\log_2$ FC (+4.17) in all significantly altered TGs. The increases of DGs, FFAs, and TGs under pathological conditions have been previously reported. For instance, lipolysis of adipose tissue increases due to EBOV infection, which converts TG to FFA and DG, and also results in enhanced recycling of the fatty acids back into TGs (9).

We observed that phosphatidylcholines (PCs) were gradually reduced over the course of COVID-19 fatalities. We also analyzed the lipids in different courses of severe and mild COVID-19 patients (S1 and S2; M1 and M2) that underwent the significant change [S1 vs. H, >1 $\log_2$ FC <-1, typically $P$ <0.05; M1 vs. H, >1 $\log_2$ FC <-1, typically $P$ <0.05] (Figure 4B and Table S9). Similar to those of metabolites, the total numbers of significantly altered lipids (>1 $\log_2$ fold change (FC) <-1, typically $P$ <0.05) in the severe and mild groups (S1 vs H, S2 vs H, M1 vs. H, and M2 vs H) were similar, which are significantly less than the number of altered lipids in the fatality group, indicating that the alterations of lipid metabolism were much more dramatic in fatal COVID-19 patients than in survivors. Besides, for either severe or mild groups of patients, many of their lipids had not returned to normal before their discharge from hospital (Figure 4B, S2 vs H and M2 vs H), even though SARS-CoV-2 could not be detected and the major clinical signs had disappeared in these patients based on official discharge criteria. Obviously, like the observations of metabolomic alterations, these discharged patients, no matter if they had experienced severe or mild symptoms, had not been fully recovered from the aftermath of COVID-19 in the aspects of both metabolite and lipid metabolisms.

Furthermore, the differentiating lipids were divided into those shared by all groups (F vs. H and S vs. H plus M vs. H) or those unique to the fatality group (F vs. H), and subsequently subjected to KEGG functional enrichment analysis. As shared by all the three symptomatic groups, differentiating lipids enriched in total 7 pathways, in particular, phosphatidylinositol signaling system, long-term depression, leishmaniasis, and inositol phosphate metabolism (Figure 5A-B and Table S10). In the case of fatality group, differentiating lipids were significantly enriched in 6 pathways, including retrograde endocannabinoid signaling, pathogenic *Escherichia coli* infection, Kaposi sarcoma-associated herpesvirus infection, glycosylphosphatidylinositol-anchor biosynthesis, glycerophospholipid metabolism and autophagy (Figure 5C-D and Table S11).

To highlight the top differentiating lipids, we showed 8 down-regulated lipids and 7 up-regulated lipids in COVID-19 patients compared with those in the healthy group (Figure 6 and Table S12), suggesting the dyslipidaemia in COVID-19 patients.

## Logistic regression and receiver operating characteristic curve analysis

To rule out the possibility of potential biomarkers induced by age difference and/or gender disparity, we developed a logistic regression model for COVID-19 patients and healthy controls. As shown in Table 1, odds ratio (OR) for age is 0.95, indicating that age does not significantly affect the five key plasma metabolites as shown in Figure 3. Although OR for gender is 3.67, the P-value is higher than 0.05, indicating that gender is not strongly associated with the metabolite panel. Therefore, either age or gender is not markedly contributed to the plasma metabolite panel highlighted by comparing COVID-19 with healthy controls.

Next, we generated ROC curves to assess the potential usefulness of plasma metabolite signatures for the diagnosis of COVID-19. Our ROC analyses revealed that combined five plasma metabolites were effective in discriminating COVID-19 patients from controls, with an area under the curve (AUC) value of 1.00 (data not shown). Then we analyzed ROC curve of each metabolite between COVID-19 and healthy subjects, revealing that malic acid and D-Xylulose 5-phosphate (Xu-5-P) show the best AUC values of 0.994 and 0.959, respectively (Figure 7A). Furthermore, the combined five plasma metabolite panel discriminating fatal group from mild group (Figure 7B), discriminating severe group from mild group (Figure 7C), and fatal group from severe group (Figure 7D) in ROC analyses, show the AUC values of 0.865, 0.708, and 0.737, respectively. Therefore, the combined five plasma metabolites could be a useful panel for COVID-19 diagnosis. Moreover, we further generated ROC curves of down-regulated lipids (Figure 7E) and up-regulated lipids (Figure 7F) for discriminating COVID-19 patients and healthy controls, respectively. Intriguingly, ROC curve analysis of glycerol 3-phosphate with an AUC value of 1.00 was observed, suggesting that circulating glycerol 3-phosphate would be a good biomarker for COVID-19. These obtained results suggest metabolomics and lipidomics provide a potential tool for disease diagnosis and drug targets in the current pandemic.

## Discussion

The main purposes of this study were to generate a high-quality resource of metabolomic and lipidomic datasets associated with COVID-19 and identify the potential biomarkers for disease diagnosis for a better understanding of the pathogenesis of COVID-19.

Among the highlighted biomarkers, malic acid and glycerol 3-phosphate showed the greatest reduction when comparing the fatality patients with healthy volunteers, and also showed dramatic reduction in both severe and mild groups. Malic acid has important physiological functions, as it can directly enter the circulation of TCA cycle to participate in human energy metabolism. Besides, malic acid can accelerate ammonia transformation to lower ammonia concentration in liver and to protect liver (23, 24). Therefore, the dramatic reduction of malic acid is consistent with the hepatic impairment associated with COVID-19. Moreover, malic acid has been found to protect endothelial cells of human blood vessels and resist damage to endothelial cells.

Xu-5-P is a metabolite of the pentose phosphate pathway that mediates the effects of carbohydrate feeding on the glycolytic pathway, as well as fatty acid and triglyceride synthesis. Xu-5-P is the coordinating signal that both activates phosphofructokinase in glycolysis and promotes transcription of the genes for lipogenesis, the hexose monophosphate shunt, and glycolysis, and is required for *de novo* synthesis of fat and hepatic energy utilization (25-28). The reduction of Xu-5-P suggests that altered glucose and lipid metabolisms are also a reflection of hepatic impairment.

Carbamoyl phosphate is an important intermediate metabolite involved in removing excess ammonia in the urea cycle (14, 15). This metabolite is the downstream product of CPSI in mitochondria of liver cells. The observed down-regulation of carbamoyl phosphate levels is associated with the severity of COVID-19, as its level in the mild patients were affected in the least extent. Importantly, the dramatic reduction of carbamoyl phosphate is usually associated with urea cycle disorder, raising the concern about the possibility of hyperammonemia and liver failure in COVID-19 patients. The postulation seems to be consistent with deficiency of malic acid as mentioned above. In addition, the metabolisms of purine and thyroid hormones were significantly altered in the fatality group. Purine metabolism mainly occurs in human liver, and the thyroid hormone can affect hepatic protein synthesis and glycogen decomposition. Therefore, our findings imply that the development of COVID-19 may cause hepatic impairment in these patients, which is consistent with the observations that a large number of COVID-19 patients showed liver function abnormalities (Table S13) (6).

Besides, the reductions of dihydrouracil, an intermediate breakdown product of uracil and guanosine monophosphate (GMP) (29), are proposed to be caused by the defects of human metabolism. It should be noted that GMP production is not only mediated by GMP synthase but also CD39 and CD73. Indeed, CD39/CD73 axis plays a crucial role in immunity and inflammation (30). Another metabolite glycerol 3-phosphate is a conserved three-carbon sugar and an obligatory component of energy-producing reactions including glycolysis and glycerolipid biosynthesis (31). Moreover, glycerol 3-phosphate is an important mobile regulator of systemic acquired resistance, which provides broad spectrum systemic immunity in response to pathogenic infections (32). These metabolites show good correlation with the progress and severity of COVID-19, and can therefore serve as biochemical indicators for immune dysfunction of this disease. Besides, SARS-CoV-2 replication would utilize nucleic acids from host cells, probably causing the metabolic perturbation, including depletion of malic acid, GMP, and carbamoyl phosphate.

Meanwhile, the lipids involved in glycerol metabolism pathway were upregulated, which maintain the balance of the energy metabolites of the body and are beneficial to the energy required for viral replication, suggesting that SARS-CoV-2 probably hijacks cellular metabolism like many other viruses (33). Our data show that the metabolic pathway of glycerophospholipids has been significantly changed, and glycerophospholipids are closely related with cardiovascular diseases. However, we did not find obvious pattern or significant difference of underlying diseases, such as hypertension, cardiac disease, diabetes, cerebrovascular disease, chronic hepatitis, and cancer, in the medical records of all the patient groups involved in this study. Therefore, this finding suggests that the fatality caused by COVID-19 might be related with cardiac impairment. Interestingly, COVID-19 has been recently reported to probably cause the loss of the smell and taste sense (34), and the KEGG analysis also showed that the taste transduction pathway is affected.

The metabolomic and lipidomic analyses also show that, although the patients in both the severe and mild symptom groups had met the official hospital discharge criteria as their COVID-19 nucleic acid tests for turning out to be negative consecutively twice and major clinical signs disappeared, many of their fundamental metabolites and lipids failed to return to normal at the time when they were discharged. This finding suggests that these discharged patients, regardless of the severity of their previous symptoms, had not been fully recovered from the disease in the aspect of metabolism. Therefore, even after the clearance of SARS-CoV-2 from patient bodies, these convalescent COVID-19 patients still need better nutrition and care that would be very helpful for their faster and full recovery from the aftermath of the disease.

The metabolomic and lipidomic alterations in patient plasma mainly reflect the systematic responses of the metabolisms of diverse cell types and organ systems that were affected by SARS-CoV-2. Therefore, the interpretations of the datasets should be integrated with other types of system studies, such as the transcriptome and proteome of specific tissue and body fluid samples, as well as clinical observations and laboratory examinations, to have a clearer and more comprehensive picture of the development of this disease. Moreover, such an integration would help us to better understand the impacts of COVID-19 to specific cells and/or tissues infected by SARS-CoV-2.

In summary, the metabolomic and lipidomic datasets of the cohort of COVID-19 patients under different symptomatic conditions are highly valuable resources for a better understanding of the host metabolic responses associated with COVID-19, which expands our knowledge about the pathogenesis of COVID-19, accelerates identification of disease biomarkers and development of diagnostic assays, and provides hints of potential therapeutic strategies.

## Materials and Methods

Materials and methods are detailed described in Supplementary data.

## Supplementary data

Supplementary methods and materials

Supplementary Figure S1-S4

Supplementary Table S1-S15

## Author contributions

D.W., T.S., X.Y. and J.-X.S. performed experiments with the help of W.L., M.H.,

Y.Y., Q.Y., T.Z., J.X., Y.W., J.M., H.W., T.T., Y.R. Y.W.; M.Z., C.Y. and S.-H.L. analyzed the metabolomics and lipidomics data with the help of D.W. and Y.Q.; S.-H.L., Y.Q., D.-Y.Z., Y.S., and X.Z. performed the experimental design and data interpretation; X.Z, Y.Q., Y.S., D.-Y.Z. and S.-H.L. analyzed the data and wrote the paper; X.Z, Y.Q., Y.S. and D.-Y.Z. designed and supervised the overall study.

**Competing Interests statement**

The authors declare no conflicts of interest.

**Acknowledgments**

We thank the patients, and the nurses and clinical staffs who are providing care for these patients. We thank the helpful discussions with Drs. Yan Wang and Yong Liu at Wuhan University. We also thank many staff members at Wuhan Jinyintan Hospital and Wuhan Metware Biotechnology Co., Ltd. for their contributions and assistance in this study. We sincerely pay tribute to our colleagues who have strived in the forefront of taking care of COVID-19 patients and are studying this novel coronavirus in Wuhan and other places around the world.

This work was supported by the Strategic Priority Research Program of CAS (XDB29010300 to X.Z.), the National Science and Technology Major Project (2020ZX09201-001 to D.-Y.Z, and 2018ZX10101004 to X.Z.), National Natural Science Foundation of China (81971818 and 81772047 to Y.S., 81873964 to Y.Q., and 31670161 to X.Z.), the Fundamental Research Funds for the Central Universities (20720200013 to S-H.L.), and Grant from Clinical Research Center for

Anesthesiology of Hubei Province (No.2019ACA167).

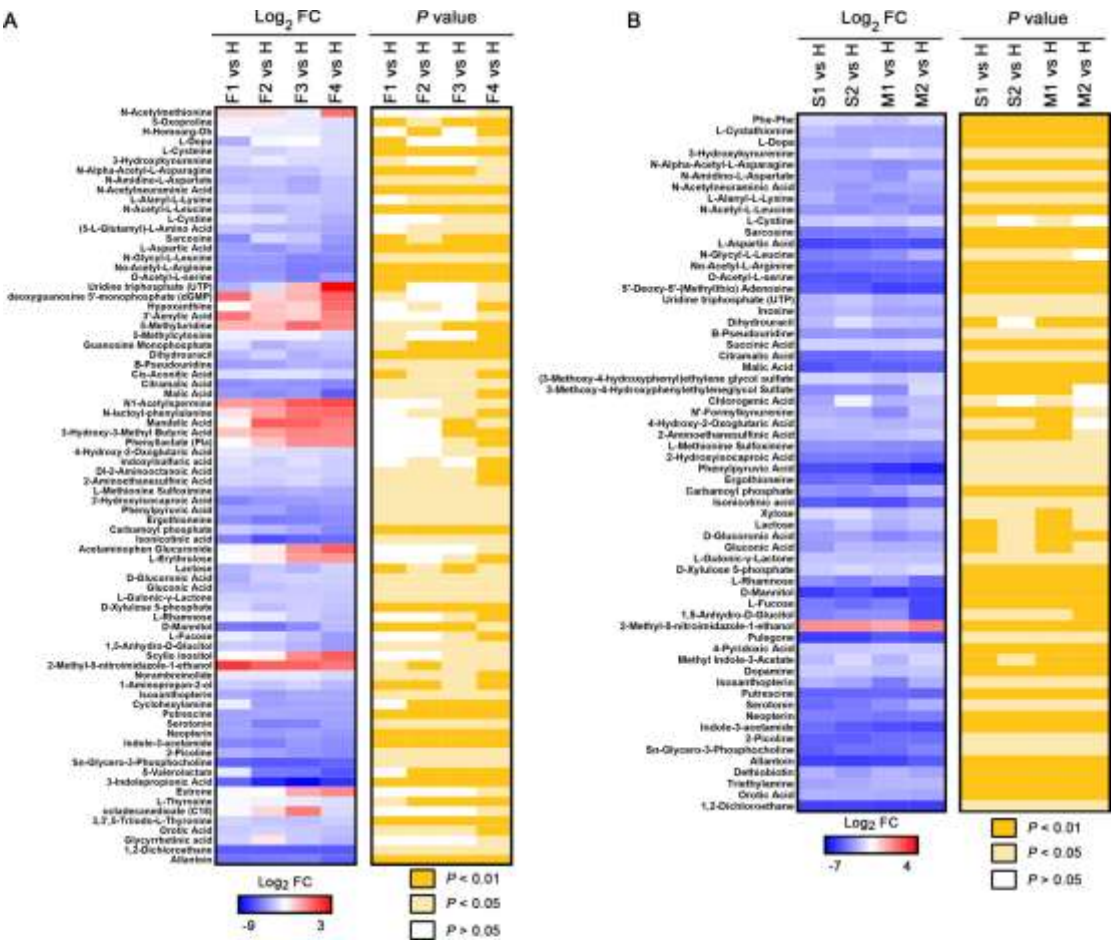

**Figure 1. COVID-19 signatures in the plasma metabolome.** Selected average plasma metabolite expression levels and associated *p* values for COVID-19 fatality patient group vs. healthy volunteer group (A), and severe or mild vs. healthy groups (B). F, fatalities, first, second, third and fourth samples, F1, F2, F3 and F4. S, severe patients, first and second samples, S1 and S2; M, mild patients, first and second samples, M1 and M2.

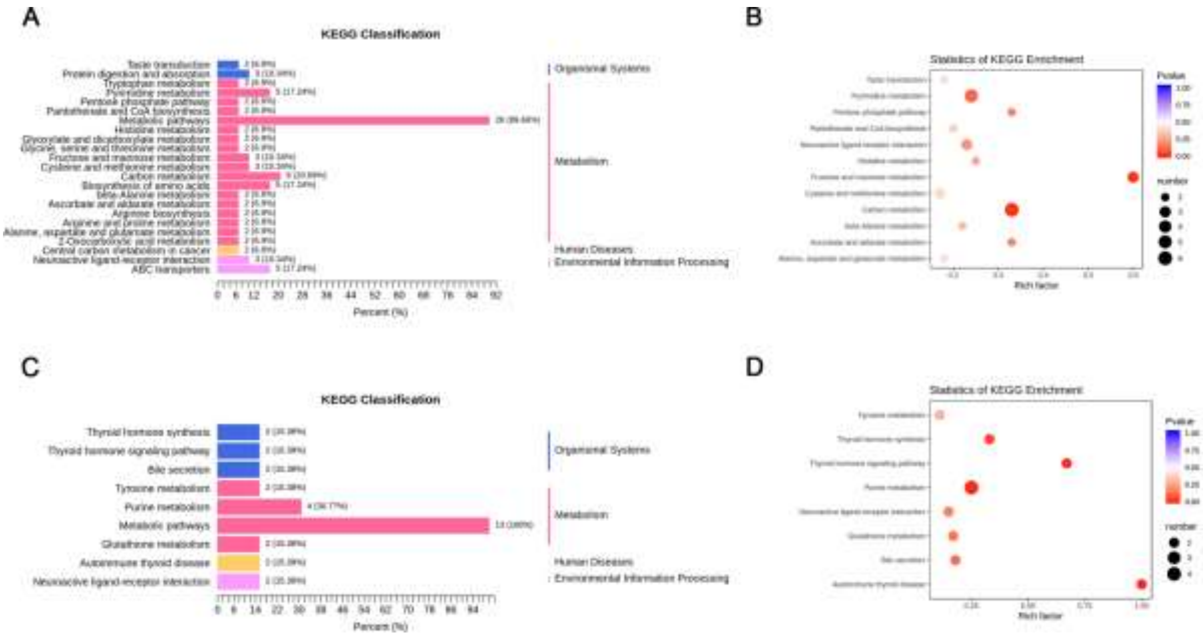

**Figure 2. The metabolome KEGG enrichment analysis of COVID-19 patient plasma.** (A-B) KEGG pathway analysis of DEMs shared in all the groups. The color of bubbles represents the value of adjusted *P* value, and the size of bubbles represents the number of counts (sorted by gene ratio). (C-D) KEGG pathway analysis of DEMs shared unique to the fatal groups.

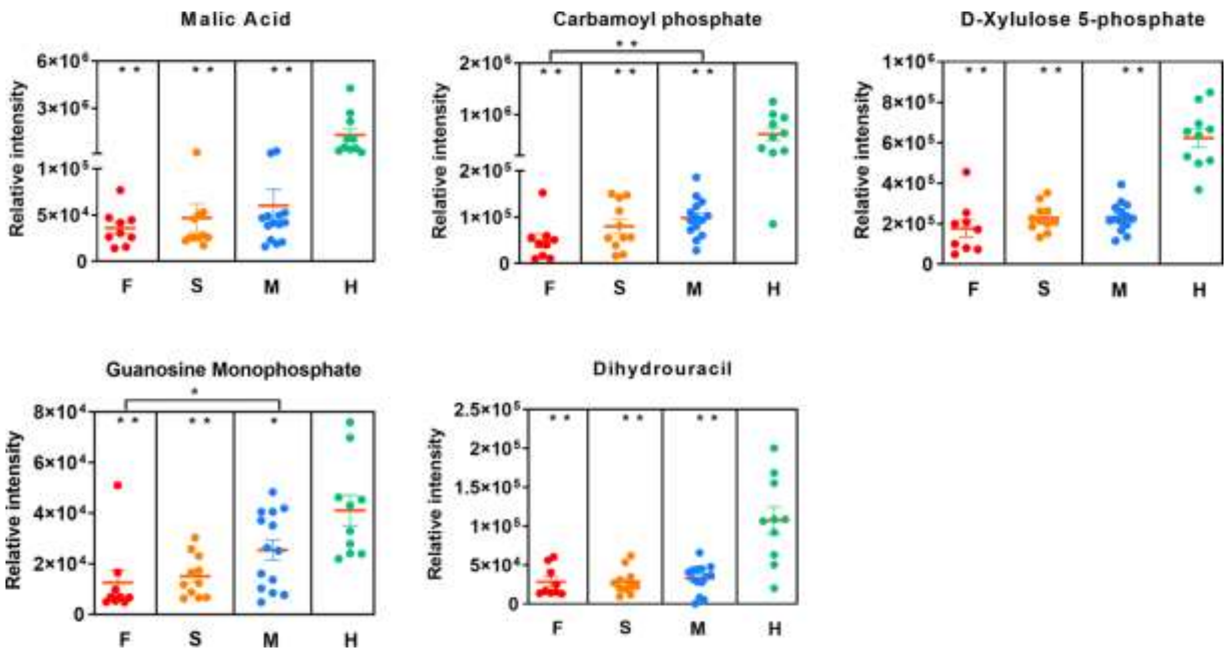

**Figure 3. The potential metabolomic biomarkers of COVID-19.** The relative intensity for each metabolite. Each dot represents a patient sample, and each patient group is differently colored as indicated. F, fatalities; S, the patients with severe symptom; M, the patients with mild symptom; H, healthy volunteers. **P*<0.05, ***P*<0.01.

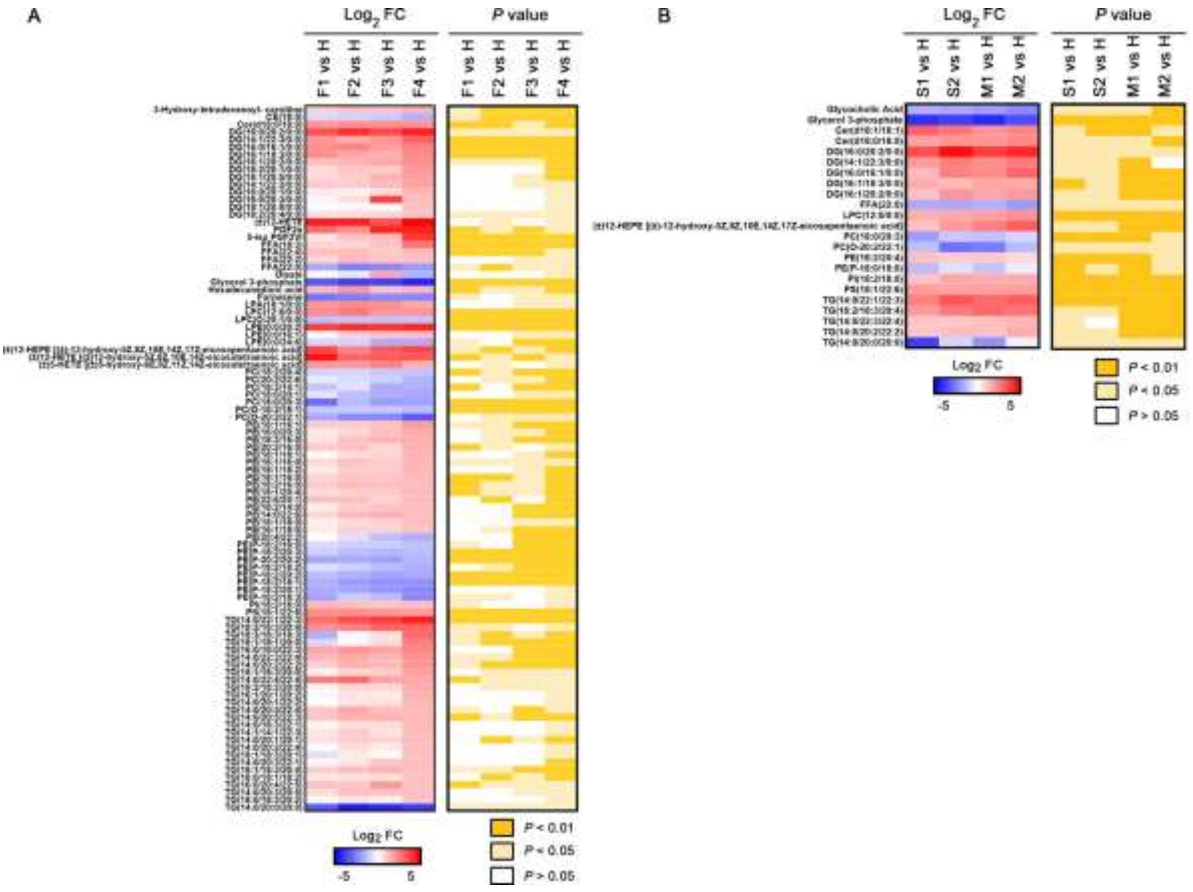

**Figure 4. COVID-19 signatures in the plasma lipidome.** Selected average plasma lipid expression levels and associated p values for COVID-19 fatality group vs. healthy volunteer group (A), and severe or mild vs. healthy groups (B). AA, arachidonic acid; BA, bile acid; CAR, carnitine; CE, cholesterol ester; Cer, ceramide; DG, diacylglycerides; TG, Triglycerides; FA, fatty acid; FFA, free fatty acids; LPA, lysophosphatidic acid; PC, phosphatidylcholine; LPC, lysophosphatidylcholine; PE, phosphatidylethanolamine; LPE, lysophosphatidyl ethanolamine; LPG, lysophosphatidylglycerol; PI, phosphatidylinositol; PS, phosphatidylserine; LPO, lipid peroxide.

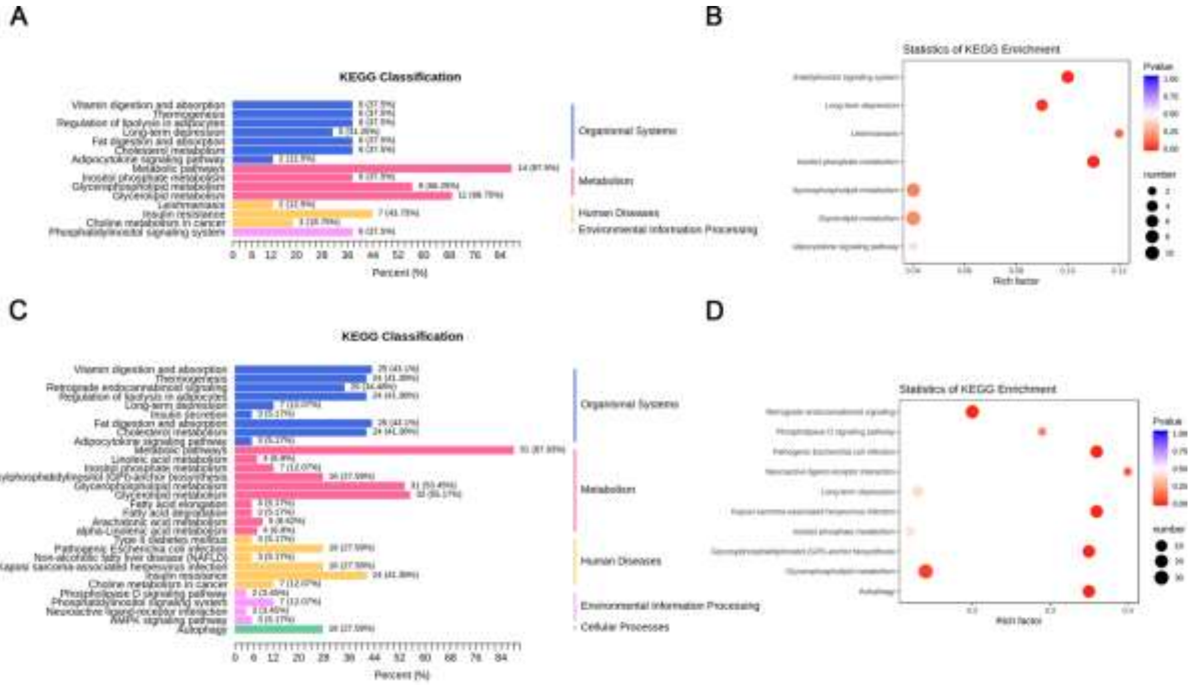

**Figure 5. The lipidome KEGG enrichment analysis of COVID-19 patients.** (A-B) KEGG pathway analysis of DEIs shared in all the groups. The color of bubbles represents the value of adjusted *P* value, and the size of bubbles represents the number of counts (sorted by gene ratio). (C-D) KEGG pathway analysis of DEIs shared unique to the fatal groups.

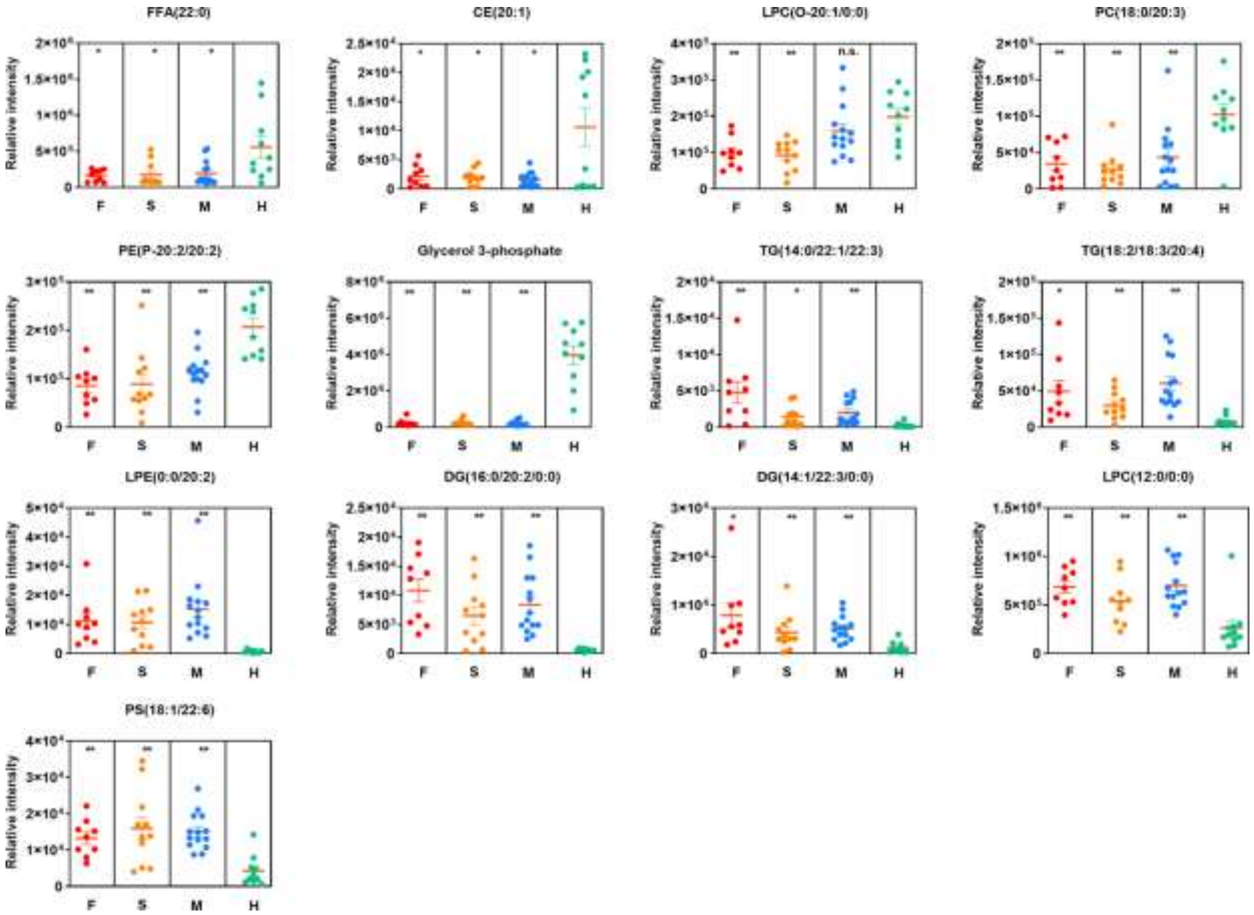

**Figure 6. The potential lipidomic biomarkers of COVID-19.** The relative intensity for each lipid. Each dot represents a patient sample, and each patient group is differently colored as indicated. F, fatalities; S, the patients with severe symptom; M, the patients with mild symptom; H, healthy volunteers. **P*<0.05, ***P*<0.01.

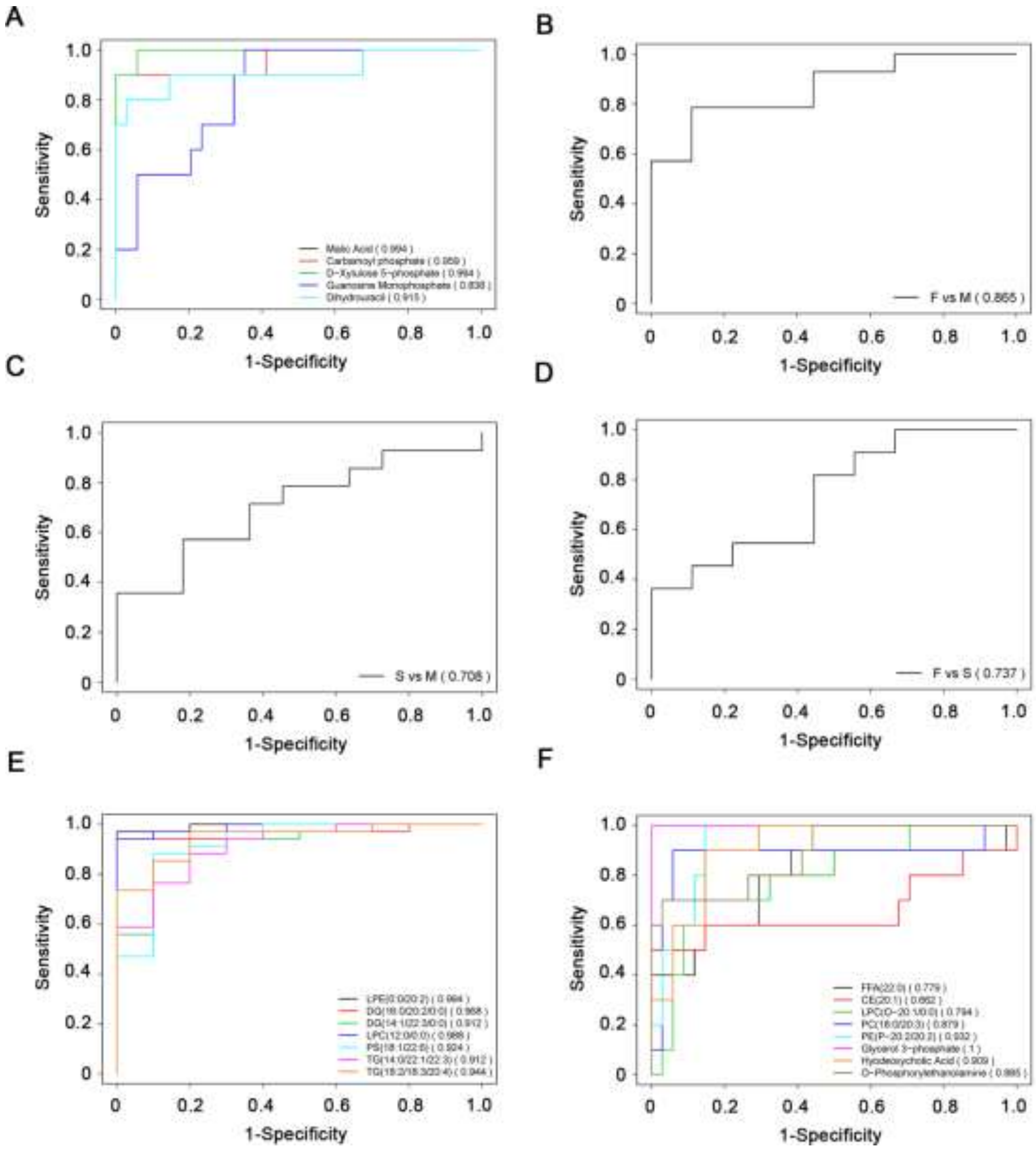

**Figure 7. ROC curve analysis for the predictive power of biomarkers for distinguishing COVID-19 patients and healthy controls.** (A) ROC curve analysis for the predictive power of each plasma metabolite for distinguishing COVID-19 groups from healthy controls; (B) ROC curve analysis for the predictive power of combined five plasma metabolites for distinguishing fatal group from mild group; (C) ROC curve analysis for the predictive power of combined five plasma metabolites for distinguishing severe group from mild group; (D) ROC curve analysis for the predictive power of combined five plasma metabolites for distinguishing fatal group from severe group; (E) ROC curve analysis for the predictive power of combined down-regulated lipids for distinguishing fatal group from healthy controls; (F) ROC curve analysis for the predictive power of combined up-regulated lipids for distinguishing fatal group from healthy controls.

**Table 1. Multivariable Analysis of the Associations of Age and Gender with COVID-19.**

| Variables | Odds Ratio | 95% CI | *P*-value [a] |
|---|---|---|---|
| **Age** | 0.95 | (0.89,1.01) | 0.14 |
| **Gender** | 3.67 | (0.80, 20.86) | 0.11 |

[a] *P* values were calculated using the 2-sided test.
