# Plasma metabolomic and lipidomic alterations associated with COVID-19

**DOI:** 10.1093/nsr/nwaa086

**Published:** 2020-04-28

**Authors:** Di Wu, Ting Shu, Xiaobo Yang, Jian-Xin Song, Mingliang Zhang, Chengye Yao, Wen Liu, Muhan Huang, Yuan Yu, Qingyu Yang, Tingju Zhu, Jiqian Xu, Jingfang Mu, Yaxin Wang, Hong Wang, Tang Tang, Yujie Ren, Yongran Wu, Shu-Hai Lin, Yang Qiu, Ding-Yu Zhang, You Shang, Xi Zhou

**Affiliations:** Joint Laboratory of Infectious Diseases and Health, Wuhan Institute of Virology & Wuhan Jinyintan Hospital, Wuhan Institute of Virology, Center for Biosafety Mega-Science, Chinese Academy of Sciences (CAS), Wuhan 430023, China; State Key Laboratory of Virology, Wuhan Institute of Virology, Center for Biosafety Mega-Science, CAS, Wuhan 430071, China; State Key Laboratory of Virology, Wuhan Institute of Virology, Center for Biosafety Mega-Science, CAS, Wuhan 430071, China; Center for Translational Medicine, Jinyintan Hospital, Wuhan 430023, China; Joint Laboratory of Infectious Diseases and Health, Wuhan Institute of Virology & Wuhan Jinyintan Hospital, Wuhan Jinyintan Hospital, Wuhan 430023, China; Department of Critical Care Medicine, Union Hospital, Tongji Medical College, Huazhong University of Science and Technology, Wuhan 430030, China; Department of Infectious Diseases, Tongji Hospital, Tongji Medical College, Huazhong University of Science and Technology, Wuhan 430030, China; Wuhan Metware Biotechnology Co., Ltd., Wuhan 430075, China; Department of Neurology, Union Hospital, Tongji Medical College, Huazhong University of Science and Technology, Wuhan 430030, China; Center for Translational Medicine, Jinyintan Hospital, Wuhan 430023, China; Joint Laboratory of Infectious Diseases and Health, Wuhan Institute of Virology & Wuhan Jinyintan Hospital, Wuhan Jinyintan Hospital, Wuhan 430023, China; Joint Laboratory of Infectious Diseases and Health, Wuhan Institute of Virology & Wuhan Jinyintan Hospital, Wuhan Institute of Virology, Center for Biosafety Mega-Science, Chinese Academy of Sciences (CAS), Wuhan 430023, China; State Key Laboratory of Virology, Wuhan Institute of Virology, Center for Biosafety Mega-Science, CAS, Wuhan 430071, China; Department of Critical Care Medicine, Union Hospital, Tongji Medical College, Huazhong University of Science and Technology, Wuhan 430030, China; State Key Laboratory of Virology, Wuhan Institute of Virology, Center for Biosafety Mega-Science, CAS, Wuhan 430071, China; Center for Translational Medicine, Jinyintan Hospital, Wuhan 430023, China; Joint Laboratory of Infectious Diseases and Health, Wuhan Institute of Virology & Wuhan Jinyintan Hospital, Wuhan Jinyintan Hospital, Wuhan 430023, China; Center for Translational Medicine, Jinyintan Hospital, Wuhan 430023, China; Joint Laboratory of Infectious Diseases and Health, Wuhan Institute of Virology & Wuhan Jinyintan Hospital, Wuhan Jinyintan Hospital, Wuhan 430023, China; Department of Critical Care Medicine, Union Hospital, Tongji Medical College, Huazhong University of Science and Technology, Wuhan 430030, China; Joint Laboratory of Infectious Diseases and Health, Wuhan Institute of Virology & Wuhan Jinyintan Hospital, Wuhan Institute of Virology, Center for Biosafety Mega-Science, Chinese Academy of Sciences (CAS), Wuhan 430023, China; State Key Laboratory of Virology, Wuhan Institute of Virology, Center for Biosafety Mega-Science, CAS, Wuhan 430071, China; Department of Critical Care Medicine, Union Hospital, Tongji Medical College, Huazhong University of Science and Technology, Wuhan 430030, China; Wuhan Metware Biotechnology Co., Ltd., Wuhan 430075, China; Wuhan Metware Biotechnology Co., Ltd., Wuhan 430075, China; Joint Laboratory of Infectious Diseases and Health, Wuhan Institute of Virology & Wuhan Jinyintan Hospital, Wuhan Institute of Virology, Center for Biosafety Mega-Science, Chinese Academy of Sciences (CAS), Wuhan 430023, China; State Key Laboratory of Virology, Wuhan Institute of Virology, Center for Biosafety Mega-Science, CAS, Wuhan 430071, China; Department of Critical Care Medicine, Union Hospital, Tongji Medical College, Huazhong University of Science and Technology, Wuhan 430030, China; State Key Laboratory of Cellular Stress Biology, Innovation Center for Cell Signaling Network, School of Life Sciences, Xiamen University, Xiamen 361005, China; Joint Laboratory of Infectious Diseases and Health, Wuhan Institute of Virology & Wuhan Jinyintan Hospital, Wuhan Institute of Virology, Center for Biosafety Mega-Science, Chinese Academy of Sciences (CAS), Wuhan 430023, China; State Key Laboratory of Virology, Wuhan Institute of Virology, Center for Biosafety Mega-Science, CAS, Wuhan 430071, China; Center for Translational Medicine, Jinyintan Hospital, Wuhan 430023, China; University of Chinese Academy of Sciences, Beijing 100049, China; Center for Translational Medicine, Jinyintan Hospital, Wuhan 430023, China; Joint Laboratory of Infectious Diseases and Health, Wuhan Institute of Virology & Wuhan Jinyintan Hospital, Wuhan Jinyintan Hospital, Wuhan 430023, China; Department of Critical Care Medicine, Union Hospital, Tongji Medical College, Huazhong University of Science and Technology, Wuhan 430030, China; Center for Translational Medicine, Jinyintan Hospital, Wuhan 430023, China; Joint Laboratory of Infectious Diseases and Health, Wuhan Institute of Virology & Wuhan Jinyintan Hospital, Wuhan Jinyintan Hospital, Wuhan 430023, China; Joint Laboratory of Infectious Diseases and Health, Wuhan Institute of Virology & Wuhan Jinyintan Hospital, Wuhan Institute of Virology, Center for Biosafety Mega-Science, Chinese Academy of Sciences (CAS), Wuhan 430023, China; State Key Laboratory of Virology, Wuhan Institute of Virology, Center for Biosafety Mega-Science, CAS, Wuhan 430071, China; Center for Translational Medicine, Jinyintan Hospital, Wuhan 430023, China; University of Chinese Academy of Sciences, Beijing 100049, China

**Keywords:** SARS-CoV-2, COVID-19, metabolome, lipidome

## Abstract

The pandemic of the coronavirus disease 2019 (COVID-19) has become a global public health crisis. The symptoms of COVID-19 range from mild to severe, but the physiological changes associated with COVID-19 are barely understood. In this study, we performed targeted metabolomic and lipidomic analyses of plasma from a cohort of patients with COVID-19 who had experienced different symptoms. We found that metabolite and lipid alterations exhibit apparent correlation with the course of disease in these patients, indicating that the development of COVID-19 affected their whole-body metabolism. In particular, malic acid of the TCA cycle and carbamoyl phosphate of the urea cycle result in altered energy metabolism and hepatic dysfunction, respectively. It should be noted that carbamoyl phosphate is profoundly down-regulated in patients who died compared with patients with mild symptoms. And, more importantly, guanosine monophosphate (GMP), which is mediated not only by GMP synthase but also by CD39 and CD73, is significantly changed between healthy subjects and patients with COVID-19, as well as between the mild and fatal cases. In addition, dyslipidemia was observed in patients with COVID-19. Overall, the disturbed metabolic patterns have been found to align with the progress and severity of COVID-19. This work provides valuable knowledge about plasma biomarkers associated with COVID-19 and potential therapeutic targets, as well as an important resource for further studies of the pathogenesis of COVID-19.

## INTRODUCTION

The outbreak of COVID-19, caused by severe acute respiratory syndrome coronavirus 2 (SARS-CoV-2), has been declared a pandemic by the World Health Organization (WHO). Up to the date of April 21, 2020, there are around 2.4 million confirmed COVID-19 cases and it has caused 162 956 deaths worldwide according to the WHO situation report. Based on a recent study of 44 672 confirmed COVID-19 cases up to February 11 by the Chinese Center for Disease Control and Prevention, over 19% of patients with COVID-19 developed severe or critical conditions [1]. The global fatality rate is around 4.8% in all the confirmed cases until March 31, and has even reached 10% in some developed countries, probably because of greater populations of elderly people [2].

The main organ attacked by SARS-CoV-2 is the lower respiratory tract, with some patients developing life-threatening acute respiratory distress syndrome (ARDS). Attacks have also been found or proposed on liver, muscle, gastrointestinal tract, lymph node, and heart by SARS-CoV-2 [3–6]. On the other hand, although more than 80% of patients with COVID-19 experienced only mild symptoms, it has been found that the conditions can rapidly progress from mild to severe, particularly in the absence of adequate medical care. Moreover, the mortality rate of COVID-19 in critically ill cases can be over 60%, posing great pressure on treatment [7]. However, the physiological changes associated with COVID-19 under different symptomatic conditions are barely understood.

Metabolites and lipids are major molecular constituents in human plasma. During critical illness, metabolic and lipid abnormalities are commonly observed, which are believed to contribute to physiology and pathology. Moreover, previous studies have demonstrated dramatic alterations of metabolome and lipidome in human plasma caused by various diseases including viral infections, such as Ebola virus disease [8,9]. Here, we performed targeted metabolomic and lipidomic profiles of plasma samples collected from a cohort of patients with COVID-19, including fatalities from COVID-19 and survivors recovered from mild or severe symptoms. Our findings here show that many of the metabolite and lipid alterations, particularly those associated with hepatic functions, align with the progress and severity of the disease, which could provide valuable knowledge about plasma biomarkers associated with COVID-19 as well as potential therapeutic targets, and shed light on the pathogenesis of COVID-19.

## RESULTS

### Study design and patients

Blood samples were harvested at Wuhan Jinyintan Hospital from patients with COVID-19, as confirmed by laboratory nucleic acid test of SARS-CoV-2 infection. Serial samples were collected over the course of disease from nine patients with fatal (F) outcome (F1–F4), 11 patients diagnosed as having severe (S) symptoms (S1–S2), and 14 patients diagnosed as having mild (M) symptoms (M1–M2) (Table S1). Of note, all the patients in the severe (S) and mild (M) groups had survived from COVID-19 and been discharged from the hospital. F1 represents the first samples collected from patients with COVID-19 who had a fatal outcome, while F4 represents the last samples before additional samples could be collected. S1 and M1 represent the samples during the disease peak of the patients in the severe or mild groups, respectively, as determined based on the *Diagnosis and Treatment Protocol for Novel Coronavirus Pneumonia* (6th edition) published by the National Health Commission of China [10], while S2 and M2 represent the last samples collected from patients in each group before the patients were discharged from the hospital. For comparison, the blood samples from 10 healthy volunteers, whose throat swabs and serological testing were negative for SARS-CoV-2, were collected. The hydrophilic and hydrophobic metabolites were extracted from each plasma sample, respectively, and measured using a liquid chromatography electrospray ionization tandem mass spectrometry (LC-ESI-MS/MS) system. Metabolite identification was conducted with use of a home-made database with retention time and ion pairs. For those metabolites without authentic standards in our database, we used MS/MS spectra to search against public databases to improve confidence in metabolite identification. The orthogonal partial least-squares discriminant analysis (OPLS-DA) was used to discriminate metabolomic profiles between the groups of patients with COVID-19 and healthy people (Figs S1–S4). In total, 431 metabolites and 698 lipids were identified and quantified, and both metabolome and lipidome showed dramatic alterations in the plasma of patients with COVID-19 (Tables S2 and S3).

### Plasma metabolomic alterations associated with clinical symptoms of COVID-19

**Figure 1.**
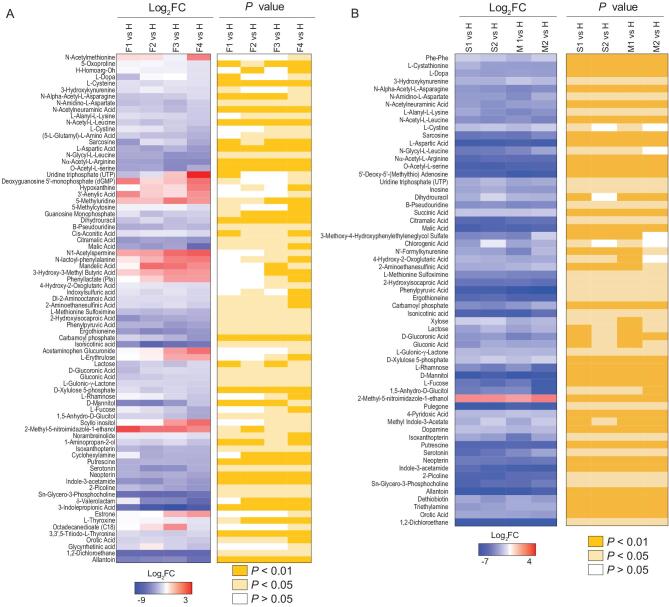
COVID-19 signatures in the plasma metabolome. Selected average plasma metabolite expression levels and associated *P* values for the COVID-19 fatality patient group vs. the healthy volunteer group (A), and severe or mild vs. healthy groups (B). F, fatalities; first, second, third and fourth samples, F1, F2, F3 and F4. S, severe patients; first and second samples, S1 and S2. M, mild patients; first and second samples, M1 and M2.

For different courses of fatalities from COVID-19 (F1–F4), we analyzed the metabolites that underwent significant change [F4 vs. H, $\mid$log_2_fold change (FC)$\mid$>1 typically *P* < 0.05]. For F vs. H, 87 of the total 431 metabolites were significantly different (*P* < 0.05) at F1, while the number of significantly altered metabolites increased to 162 at F4 in the fatalities; and most of the changes were down-regulated (Table S2). We found a positive correlation between the alteration of metabolites and the course of disease deterioration in patients with a fatal outcome (Fig. 1A and Table S4), indicating that the development of disease affects the metabolism of metabolites.

We also profiled the metabolites in the different courses of severe and mild COVID-19 (S1 and S2; M1 and M2), and analyzed those that underwent significant change [S1 vs. H, $\mid$log_2_FC$\mid$>1, typically *P* < 0.05; M1 vs. H, $\mid$log_2_FC$\mid$>1, typically *P* < 0.05] (Fig. 1B and Table S5). There are apparently fewer metabolites with significant changes ($\mid$log_2_FC$\mid$>1, typically *P* < 0.05) observed in patient groups with severe and mild symptoms when compared with those of patients that died, and almost all the significantly altered metabolites were down-regulated. These results indicate that the alterations of metabolic pathways were more extensive in fatal COVID-19 cases than in patients with severe and mild symptoms who survived.

In addition, it is noteworthy that although the patients in both the severe and mild groups had met the hospital discharge criteria at time points S2 and M2, in that their COVID-19 nucleic acid tests were negative twice consecutively, our metabolomic data clearly show that many of their metabolites had not returned to normal levels when compared with those in healthy volunteers (Fig. 1B), suggesting that these discharged patients had not fully recovered from the physiological impacts of COVID-19.

**Figure 2.**
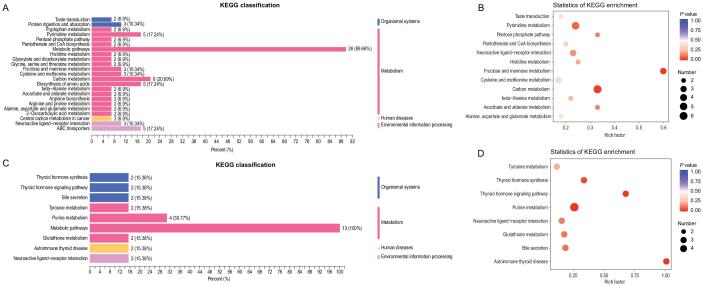
Metabolome KEGG enrichment analysis of plasma from patients with COVID-19. (A, B) KEGG pathway analysis of differentiating metabolites shared in all the groups. The color of bubbles represents the value of adjusted *P* value, and the size of bubbles represents the number of counts (sorted by gene ratio). (C, D) KEGG pathway analysis of differentiating metabolites shared unique to the fatal groups.

To further analyze the metabolomic data, the differentiating metabolites were divided into those shared by all groups (F vs. H, S vs. H, and M vs. H) or those unique to the fatal group (F vs. H). Then, we performed Kyoto Encyclopedia of Genes and Genomes (KEGG) functional enrichment analysis to annotate the potential functional implication of differentiating metabolites among these groups (Fig. 2). In all three symptomatic groups, the differentiating metabolites were enriched in 12 metabolic pathways and the most important three pathways were highlighted including pyrimidine metabolism, fructose and mannose metabolism, and carbon metabolism (Fig. 2A and B; Table S6).

In the fatality group, differentiating metabolites significantly enriched in four additional pathways: thyroid hormone synthesis, thyroid hormone signaling, purine metabolism, and autoimmune thyroid

(Fig. 2C and D; Table S7), suggesting that the alterations in these pathways are associated with the progress and deterioration of COVID-19.

**Figure 3.**
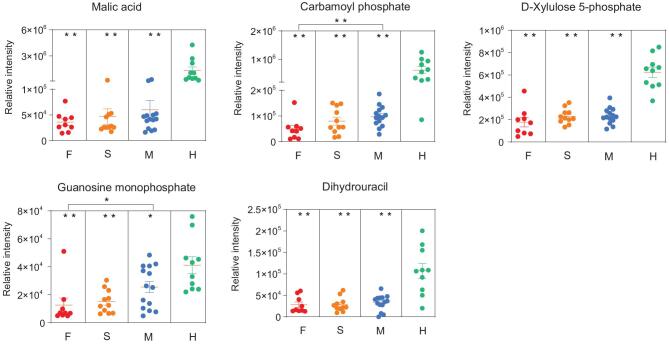
Potential metabolomic biomarkers of COVID-19, in terms of the relative intensity for each metabolite. Each dot represents a patient sample, and each patient group is differently colored as indicated. F, fatalities; S, the patients with severe symptom; M, the patients with mild symptom; H, healthy volunteers. ^*^P < 0.05, ^*^^*^P < 0.01.

A prominent signature observed among patients who died from COVID-19 was an acute reduction of metabolites in patient plasma with the aggravation of the course of COVID-19. By comparing healthy subjects and fatal cases, we highlighted the top five differentiating metabolites (Fig. 3). For instance, malic acid, an intermediate of the tricarboxylic acid (TCA) cycle, exhibited the greatest log_2_FC (–5.2) among all significantly altered metabolites in the fatalities. Similarly, aspartic acid was markedly down-regulated in the plasma of patients (Tables S4 and S5). Thereby, we postulate that deficiencies in malic acid and aspartate are caused, at least in part, by SARS-CoV-2 replication hijacking nucleic acids from host cells, because the TCA cycle and aspartate would be preferential for purine and pyrimidine nucleotide biosynthesis. Another differentiating metabolite guanosine monophosphate (GMP) was observed, important for metabolic reactions mediated not only by GMP synthase but also CD39 and CD73. Both CD39 and CD73 are immunomodulatory enzymes, suggesting that CD39/CD73 axis imbalance may

occur in patients with COVID-19. In addition, we observed that the level of carbamoyl phosphate was significantly and gradually reduced over the course of COVID-19 fatalities. Carbamoyl phosphate is synthesized from free amino donors by carbamoyl phosphate synthetase I (CPSI) in mitochondria of liver cells, and participates in the urea cycle to remove excess ammonia and produce urea [11–14]. Its reduction in fatal cases of COVID-19 suggests the possibility of liver damage, which could also impair amino acid and pyrimidine metabolisms as CPSI can also maintain the pyrimidine pool. Notably, both GMP and carbamoyl phosphate show significant changes between fatal and mild patients, indicating that the disease progression is associated with immune dysfunction and nucleotide metabolism.

### Plasma lipidomic alterations correspond to clinical symptoms of COVID-19

**Figure 4.**
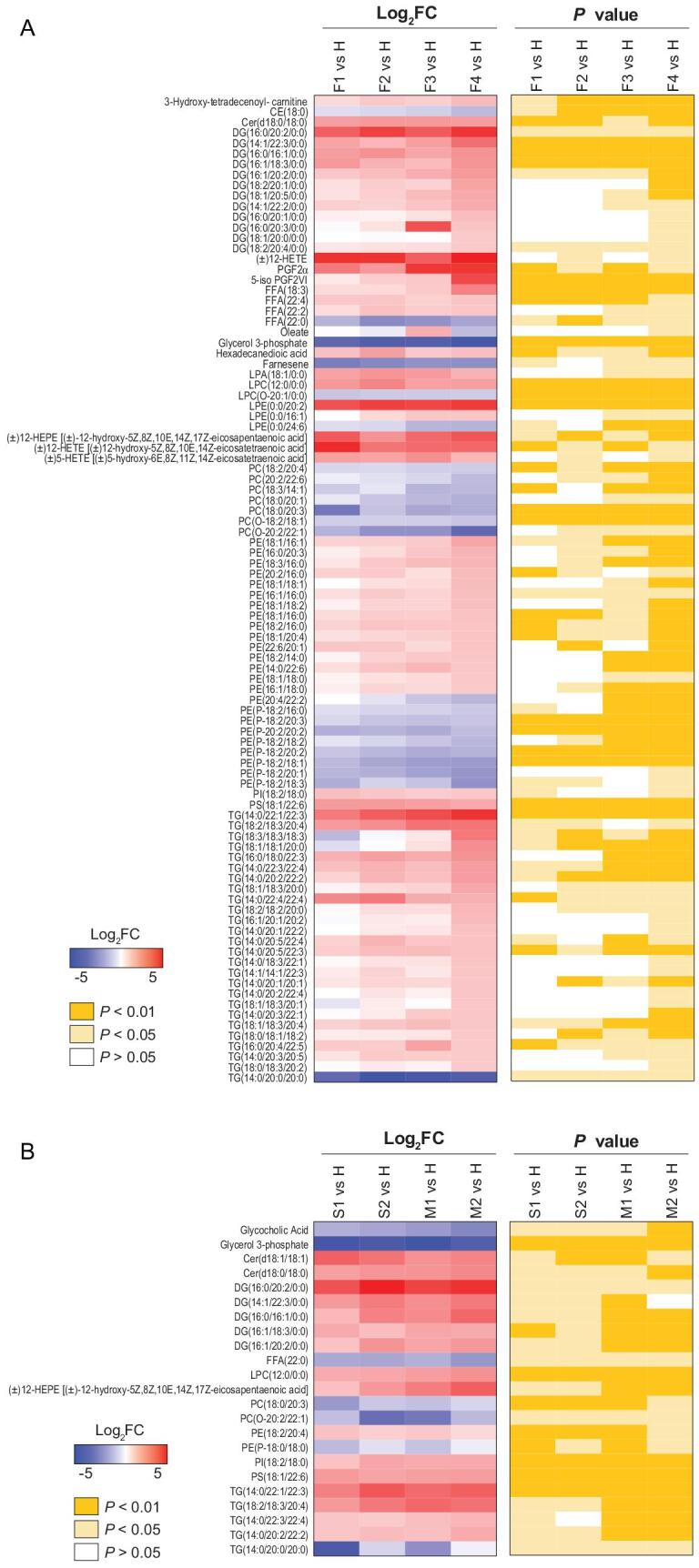
COVID-19 signatures in the plasma lipidome. Selected average plasma lipid expression levels and associated *P* values for the COVID-19 fatality group vs. the healthy volunteer group (A), and severe or mild vs. healthy groups (B). AA, arachidonic acid; BA, bile acid; CAR, carnitine; CE, cholesterol ester; Cer, ceramide; DG, diacylglycerides; TG, triglycerides; FA, fatty acid; FFA, free fatty acids; LPA, lysophosphatidic acid; PC, phosphatidylcholine; LPC, lysophosphatidylcholine; PE, phosphatidylethanolamine; LPE, lysophosphatidyl ethanolamine; LPG, lysophosphatidylglycerol; PI, phosphatidylinositol; PS, phosphatidylserine; LPO, lipid peroxide.

We analyzed the lipids in different courses of fatal COVID-19 cases (F1–F4) that underwent significant change [F4 vs. H, $\mid$log_2_FC$\mid$>1, typically *P* < 0.05]. Most of the significantly changed lipids are up-regulated and a positive correlation could be readily observed between the alteration of lipids and the course of disease deterioration in the fatal cases (Fig. 4A and Table S8). Lipid subclasses including diglycerides (DGs), free fatty acids (FAAs), and triglycerides (TGs), were identified in higher abundance in the fatality group (F vs. H), and the relative abundances of these lipids increased with deterioration of the disease. In particular, DG(16:0/20:2/0:0) exhibited the greatest log_2_FC (+4.15) in DGs, and TG(14:0/22:1/22:3) exhibited the greatest log_2_FC (+4.17) in all significantly altered TGs. Increases in DGs, FFAs, and TGs under pathological conditions have been previously reported. For instance, lipolysis of adipose tissue increases because of EBOV infection, which converts TG to FFA and DG, and also results in enhanced recycling of the fatty acids back into TGs [9].

We observed that phosphatidylcholines (PCs) were gradually reduced over the course of COVID-19 fatal cases. We also analyzed the lipids in different courses of severe and mild COVID-19 (S1 and S2; M1 and M2) that underwent significant change [S1 vs. H, $\mid$log_2_FC$\mid$>1, typically *P* < 0.05; M1 vs. H, $\mid$log_2_FC$\mid$>1, typically *P* < 0.05] (Fig. 4B and Table S9). As for metabolites, the total numbers of significantly altered lipids ($\mid$log_2_FC$\mid$>1, typically *P* < 0.05) in the severe and mild groups (S1 vs. H, S2 vs. H, M1 vs. H, and M2 vs. H) were similar, but these were significantly less than the number of altered lipids in the fatality group, indicating that alterations of lipid metabolism were much more dramatic in fatal COVID-19 cases than in survivors. For patients with either severe or mild symptoms, many of their lipids had not returned to normal before discharge from hospital (Fig. 4B, S2 vs. H and M2 vs. H), even though SARS-CoV-2 could not be detected and the major clinical signs had disappeared in these patients based on official discharge criteria. Clearly, as noted for observations of metabolomic alterations, these discharged patients, no matter if they had experienced severe or mild symptoms, had not fully recovered from the aftermath of COVID-19 in the aspects of both metabolite and lipid metabolisms.

**Figure 5.**
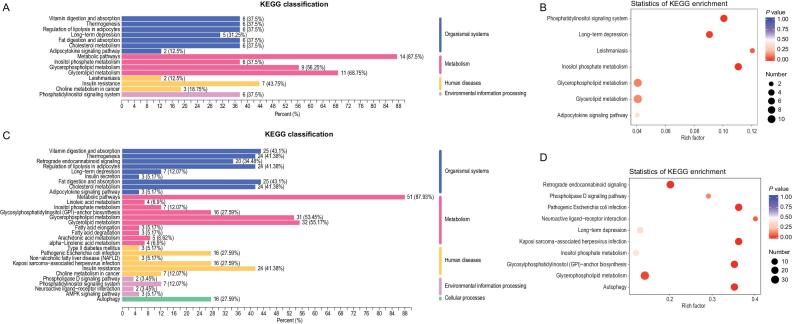
Lipidome KEGG enrichment analysis of patients with COVID-19. (A,B) KEGG pathway analysis of differentiating lipids shared in all the groups. The color of bubbles represents the value of adjusted *P* value, and the size of bubbles represents the number of counts (sorted by gene ratio). (C,D) KEGG pathway analysis of differentiating lipids shared unique to the fatal groups.

Furthermore, the differentiating lipids were divided into those shared by all groups (F vs. H and S vs. H plus M vs. H) or those unique to the fatality group (F vs. H), and subsequently subjected to KEGG functional enrichment analysis. As shared by all the three symptomatic groups, the differentiating lipids were enriched for highlighting seven metabolic pathways, in particular, phosphatidylinositol signaling system, long-term depression, leishmaniasis, and inositol phosphate metabolism (Fig. 5A and B; Table S10). In the case of the fatality group, differentiating lipids were significantly enriched in six pathways: retrograde endocannabinoid signaling, pathogenic *Escherichia coli* infection, Kaposi sarcoma-associated herpesvirus infection, glycosylphosphatidylinositol-anchor biosynthesis, glycerophospholipid metabolism, and autophagy (Fig. 5C and D; Table S11).

**Figure 6.**
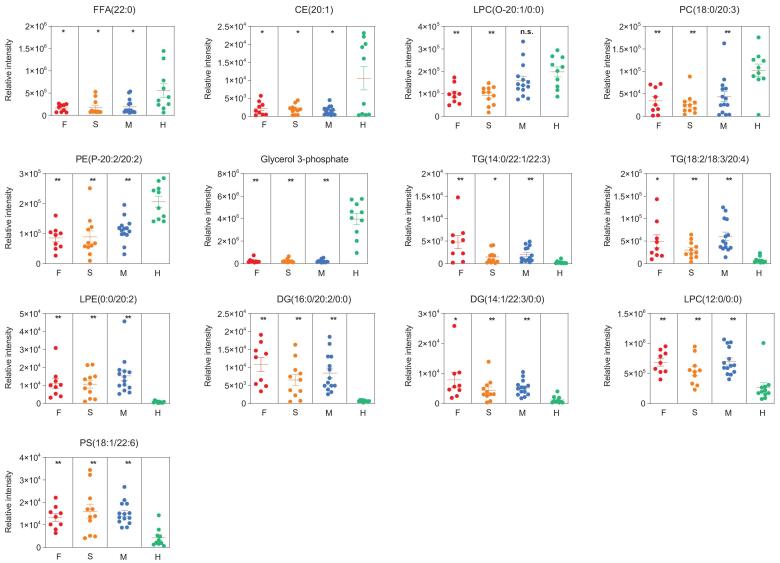
Potential lipidomic biomarkers of COVID-19, in terms of the relative intensity for each lipid. Each dot represents a patient sample, and each patient group is differently colored as indicated. F, fatalities; S, patients with severe symptoms; M, patients with mild symptoms; H, healthy volunteers. ^*^P < 0.05, ^*^^*^P < 0.01.

To highlight the top differentiating lipids, we detailed eight down-regulated lipids and seven up-regulated lipids in patients with COVID-19 compared with those in the healthy group (Fig. 6 and Table S12), suggesting dyslipidemia in patients with COVID-19.

### Logistic regression and receiver operating characteristic curve analysis

To rule out the possibility of potential biomarkers induced by age difference and/or gender disparity, we developed a logistic regression model for patients with COVID-19 and healthy controls. As shown in Table 1, the odds ratio (OR) for age is 0.95, indicating that age does not significantly affect the five key plasma metabolites (Fig. 3). Although OR for gender is 3.67, the *P* value is higher than 0.05, indicating that gender is not strongly associated with the metabolite panel. Therefore, neither age nor gender markedly contribute to the plasma metabolite panel highlighted by comparing COVID-19 with healthy controls.

**Table 1.** Multivariable analysis of the associations of age and gender with COVID-19.

Variables	Odds ratio	95% CI	*P* value^a^
Age	0.95	(0.89, 1.01)	0.14
Gender	3.67	(0.80, 20.86)	0.11

^a^ *P* values were calculated using the 2-sided test.

**Figure 7.**
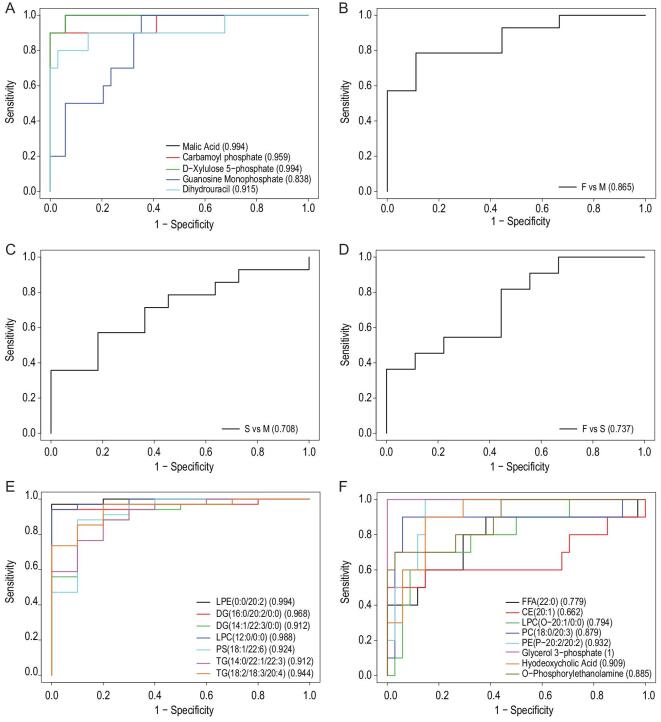
ROC curve analysis for the predictive power of biomarkers for distinguishing between patients with COVID-19 and healthy controls. (A) ROC curve analysis for the predictive power of each plasma metabolite for distinguishing COVID-19 groups from healthy controls. (B) ROC curve analysis for the predictive power of combined five plasma metabolites for distinguishing fatal group from mild group. (C) ROC curve analysis for the predictive power of combined five plasma metabolites for distinguishing severe group from mild group. (D) ROC curve analysis for the predictive power of combined five plasma metabolites for distinguishing fatal group from severe group. (E) ROC curve analysis for the predictive power of combined down-regulated lipids for distinguishing fatal group from healthy controls. (F) ROC curve analysis for the predictive power of combined up-regulated lipids for distinguishing fatal group from healthy controls.

Next, we generated ROC curves to assess the potential usefulness of plasma metabolite signatures for diagnosis of COVID-19. Our ROC analyses revealed that the combined five plasma metabolites were effective in discriminating COVID-19 patients from controls, with an area under the curve (AUC) value of 1.00 (data not shown). Then we analyzed the ROC curve of each metabolite between COVID-19 and healthy subjects, revealing that malic acid and D-xylulose 5-phosphate (Xu-5-P) show the best AUC values of 0.994 and 0.959, respectively (Fig. 7A). Furthermore, the combined five plasma metabolite panel discriminating the fatal group from mild group (Fig. 7B), severe group from the mild group (Fig. 7C), and fatal group from the severe group (Fig. 7D) in ROC analyses, show AUC values of 0.865, 0.708, and 0.737, respectively. Therefore, the combined five plasma metabolites could be a useful panel for COVID-19 diagnosis. Moreover, we further generated ROC curves of down-regulated lipids (Fig. 7E) and up-regulated lipids (Fig. 7F) for discriminating between patients with COVID-19 and healthy controls, respectively. Intriguingly, ROC curve analysis of glycerol 3-phosphate with an AUC value of 1.00 was observed, suggesting that circulating glycerol 3-phosphate would be a good biomarker for COVID-19. These obtained results suggest that metabolomics and lipidomics provide a potential tool for disease diagnosis and drug targets in the current pandemic.

## DISCUSSION

The main purposes of this study were to generate a high-quality resource of metabolomic and lipidomic datasets associated with COVID-19 and identify potential biomarkers for disease diagnosis for a better understanding of the pathogenesis of COVID-19.

Among the highlighted biomarkers, malic acid and glycerol 3-phosphate showed the greatest reduction when comparing patients who died with healthy volunteers, but also showed dramatic reduction in groups with severe and mild symptoms. Malic acid has important physiological functions, as it can directly enter the TCA cycle to participate in human energy metabolism. It can also accelerate ammonia transformation to lower the concentration of ammonia in the liver and, therefore, protect it [15,16]. The dramatic reduction of malic acid is consistent with the hepatic impairment associated with COVID-19. Moreover, malic acid has been found to protect endothelial cells of human blood vessels and prevent damage to endothelial cells.

Xu-5-P is a metabolite of the pentose phosphate pathway that mediates the effects of carbohydrate feeding on the glycolytic pathway, as well as fatty acid and triglyceride synthesis. Xu-5-P is the coordinating signal that activates phosphofructokinase in glycolysis and promotes transcription of the genes for lipogenesis, the hexose monophosphate shunt, and glycolysis, and is required for *de novo* synthesis of fat and hepatic energy use [17–20]. The reduction of Xu-5-P suggests that altered glucose and lipid metabolisms are also a reflection of hepatic impairment.

Carbamoyl phosphate is an important intermediate metabolite involved in removing excess ammonia in the urea cycle [11,^12^]. This metabolite is the downstream product of CPSI in mitochondria of liver cells. The observed down-regulation of carbamoyl phosphate levels is associated with the severity of COVID-19, as its level in the mild patients was affected to the least extent. Importantly, dramatic reduction in carbamoyl phosphate is usually associated with urea cycle disorder, raising concern about the possibility of hyperammonemia and liver failure in patients with COVID-19. This postulation seems to be consistent with deficiency of malic acid, as mentioned above. In addition, the metabolisms of purine and thyroid hormones were significantly altered in the fatality group. Purine metabolism mainly occurs in human liver, and the thyroid hormone can affect hepatic protein synthesis and glycogen decomposition. Therefore, our findings imply that development of COVID-19 may cause hepatic impairment in these patients, which is consistent with the observations that a large number of patients with COVID-19 patients showed liver function abnormalities (Table S13) [6].

Reduction in dihydrouracil, an intermediate breakdown product of uracil and guanosine monophosphate (GMP) [21], is proposed to be caused by defects of human metabolism. It should be noted that GMP production is not only mediated by GMP synthase but also by CD39 and CD73. Indeed, the CD39/CD73 axis plays a crucial role in immunity and inflammation [22]. Another metabolite, glycerol 3-phosphate, is a conserved three-carbon sugar and an obligatory component of energy-producing reactions including glycolysis and glycerolipid biosynthesis [23]. Glycerol 3-phosphate is an important mobile regulator of systemic acquired resistance, which provides broad spectrum systemic immunity in response to pathogenic infections [24]. These metabolites show good correlation with the progress and severity of COVID-19, and could, therefore, serve as biochemical indicators for immune dysfunction of this disease. SARS-CoV-2 replication uses nucleic acids from host cells, probably causing the metabolic perturbation, including depletion of malic acid, GMP, and carbamoyl phosphate.

The lipids involved in the glycerol metabolism pathway, which maintains the balance of the energy metabolites in the body, were up-regulated, suggesting that SARS-CoV-2, like many other viruses, probably hijacks cellular metabolism [25]. Our data show that the metabolic pathway of glycerophospholipids, closely related with cardiovascular diseases, was significantly changed in patients with COVID-19. We did not find any obvious pattern or significant difference in underlying diseases, such as hypertension, cardiac disease, diabetes, cerebrovascular disease, chronic hepatitis, and cancer, taken from the patient’ medical records of patients, but fatality from COVID-19 could be related to cardiac impairment. Interestingly, it has been recently reported that COVID-19 can cause loss of the senses of smell and taste [26], and our KEGG analysis also showed that the taste transduction pathway is affected.

The metabolomic and lipidomic analyses also show that, although patients in both the severe and mild symptom groups had met the official hospital discharge criteria in that their COVID-19 nucleic acid tests for were negative twice consecutively, and major clinical signs had disappeared, many of their fundamental metabolites and lipids had not returned to normal by the time they were discharged. It is suggested that the discharged patients, regardless of the severity of their previous symptoms, had not fully recovered from the disease in the aspect of metabolism. Therefore, even after the clearance of SARS-CoV-2 from patients’ bodies, patients in convalescence could benefit from better nutrition and care for faster and full recovery from the disease.

The metabolomic and lipidomic alterations in patient plasma mainly reflect the systematic responses of the metabolisms of diverse cell types and organ systems affected by SARS-CoV-2. Therefore, the interpretations of the datasets should be integrated with other types of system studies, such as the transcriptome and proteome of specific tissue and body fluid samples, as well as clinical observations and laboratory examinations, to provide a clearer and more comprehensive picture of the development of this disease. Moreover, such integration would help us to better understand the impacts of COVID-19 on specific cells and/or tissues infected by SARS-CoV-2.

In summary, the metabolomic and lipidomic datasets of cohorts of patients with COVID-19 under different symptomatic conditions are highly valuable resources for a better understanding of the host metabolic responses associated with the disease, to expand our knowledge about the pathogenesis, accelerate identification of disease biomarkers and development of diagnostic assays, and provide hints for potential therapeutic strategies.

## MATERIALS AND METHODS

Materials and methods are detailed described in Supplementary data.

## Supplementary Material

nwaa086_Supplemental_File
