# Supplementary material for: Plasma metabolomic and lipidomic alterations associated with COVID-19
Source: Natl Sci Rev. 2020 Apr 28;7(7):1157–68. doi: 10.1093/nsr/nwaa086 (PMC7197563; doi:10.1093/nsr/nwaa086)
Supplement: nwaa086_Supplemental_File [file nwaa086_supplemental_file.zip › Wu-Tables S1-S15.pdf]

Table S1. Study design and patients

	Fatal (F)				Severe (S)		Mild (M)		Healthy (H)
	T1	T2	T3	T4	T1	T2	T1	T2	H
**Onset to hospitalization**	1. 8	5.1	10.1	14.8	5. 4	15.4	5.2	13	NA
Days (SD)	(0.4)	(0.3)	(0.3)	(1.2)	(3.1)	(4.8)	(0.6)	(0)	NA
**Sex**									
Female	5				8		9		5
Male	4				3		5		5
**Age**	64.6				57.4		45.9		48.7
Mean (SD)	(8.5)				(12.5)		(11.8)		(9.6)
**Patients**	9				11		14		10
Sample number	(36)				(22)		(28)		(10)

Table S2. Overview of total changed metaboites

Total metaboites (431)	Metabolites（p <0.05）	Up-regulated	wn-regula	ax (Log$_2$F	Min (Log$_2$FC)
**F1 vs. H**	87	4	83	2.17	-5.19
**F2 vs. H**	164	19	145	1.95	-8.98
**F3 vs. H**	172	45	127	2.6	-8.66
**F4 vs. H**	162	51	111	2.87	-6.67
**S1 vs. H**	142	23	119	1.73	-6.79
**S2 vs. H**	154	24	130	1.68	-7.03
**M1 vs. H**	190	28	162	1.36	-5.58
**M2 vs. H**	203	49	154	1.97	-8.29

Table S2. Overview of total changed lipids

Total lipid (698)	Lipids （p <0.05）	Up-regulated	Down-regulated	Max (Log_2FC)	Min (Log_2FC)
**F1 vs. H**	255	111	144	4.54	-3.02
**F2 vs. H**	203	134	69	4.2	-3.6
**F3 vs. H**	221	135	86	4.23	-3.6
**F4 vs. H**	248	152	96	2.29	-4.1
**S1 vs. H**	157	57	100	5.37	-4.08
**S2 vs. H**	158	104	54	4.76	-3.78
**M1 vs. H**	120	82	38	4.44	-4.23
**M2 vs. H**	127	93	34	5.61	-3.47

Table S4. Metabolomics data of F vs H

		Log_2 FC				***P*** **value**				**FDR**			
Compounds	Class	F1/H	F2/H	F3/H	F4/H	F1/H-P	F2/H-P	F3/H-P	F4/H-P	F1/H-FDR	F2/H-FDR	F3/H-FDR	F4/H-FDR
N-Acetylmethionine	Amino acid metabolome	0.415994917	0.384180519	-0.520902749	1.472418955	0.2871537	0.34377689	0.17238011	0.02486072	0.357697	0.396171	0.253569	0.098111
5-Oxoproline	Amino acid metabolome	-0.447918969	-0.371686393	-0.602655136	-1.001115079	0.00443867	0.01061575	0.00112247	3.2844E-05	0.037522	0.051512	0.015572	0.002827
H-Homoarg-Oh	Amino acid metabolome	-0.280135404	-0.584682436	-0.563126941	-1.052391897	0.11015728	0.00797714	0.05488502	0.00111958	0.203767	0.045225	0.132896	0.014459
L-Dopa	Amino acid metabolome	-2.441226333	-0.235731174	0.0001691	-1.054779217	0.00191993	0.38296416	0.49991836	0.03253759	0.021776	0.419807	0.499918	0.108711
L-Cysteine	Amino acid metabolome	-1.071199628	-1.439037259	-0.972533476	-1.063727456	0.0008726	0.00014244	0.00500835	0.00082683	0.014238	0.004093	0.040728	0.013199
3-Hydroxykynurenine	Amino acid metabolome	-1.177065918	-0.575286888	-1.122272826	-1.250957275	0.04570095	0.16365476	0.05331719	0.04320105	0.126145	0.249241	0.130567	0.122452
N-Alpha-Acetyl-L-Asparagine	Amino acid metabolome	-2.106915553	-1.969521205	-1.868188579	-1.28968066	0.00110143	0.00137539	0.00201758	0.02126469	0.014485	0.013556	0.022147	0.088981
N-Amidino-L-Aspartate	Amino acid metabolome	-2.260634457	-2.01719017	-2.629166494	-1.793526857	0.02105796	0.0263016	0.01654673	0.03175842	0.086907	0.092163	0.074333	0.106937
N-Acetylneuraminic Acid	Amino acid metabolome	-1.660029412	-1.93638719	-2.437826541	-1.863045695	0.00357033	0.00223934	0.00122678	0.0025072	0.033452	0.019303	0.016523	0.027015
L-Alanyl-L-Lysine	Amino acid metabolome	-1.140709361	-1.428848574	-1.516403339	-1.875582527	0.07409744	0.04420242	0.03999528	0.02602591	0.168974	0.11907	0.109104	0.099267
N-Acetyl-L-Leucine	Amino acid metabolome	-1.762353717	-2.309423849	-1.782895081	-1.949248479	0.00015516	3.5937E-05	0.00010697	0.00010429	0.007268	0.002581	0.005123	0.005619
L-Cystine	Amino acid metabolome	-0.806890274	-0.976201639	-1.795300507	-2.469899382	0.13856428	0.10713298	0.03547641	0.02068592	0.234201	0.194828	0.103146	0.088319
(5-L-Glutamyl)-L-Amino Acid	Amino acid metabolome	-1.715117389	-2.255227468	-2.261791293	-2.71761163	0.06058035	0.04127386	0.0413158	0.03293674	0.149986	0.113306	0.110603	0.109198
Sarcosine	Amino acid metabolome	-3.612929377	-1.113695858	-1.641170407	-3.051463298	5.9217E-05	0.02179128	0.00293875	7.778E-05	0.005301	0.082296	0.029315	0.00479
L-Aspartic Acid	Amino acid metabolome	-2.916577992	-3.291196182	-2.606249992	-3.466167213	0.00105208	0.00086573	0.00132824	0.00080486	0.014485	0.010403	0.017348	0.013199
N-Glycyl-L-Leucine	Amino acid metabolome	-3.008074611	-3.067149388	-3.778841704	-3.725162489	0.03744406	0.0367998	0.03074046	0.03108619	0.114457	0.107896	0.096709	0.106937
Nα-Acetyl-L-Arginine	Amino acid metabolome	-3.292069569	-3.325872506	-4.003237843	-4.044157808	0.00139963	0.00135444	0.00110141	0.00108834	0.017235	0.013556	0.015572	0.014459
O-Acetyl-L-serine	Amino acid metabolome	-3.234102794	-3.124768019	-4.045101323	-2.95324936	0.00025287	0.00024919	0.0001859	0.00029571	0.007268	0.004882	0.006677	0.007966
Uridine triphosphate (UTP)	Nucleotide metabolome	-2.352669171	-1.108219394	0.698174055	2.870307136	0.00881366	0.06646519	0.22913253	0.03822944	0.056697	0.151735	0.310554	0.116857
deoxyguanosine 5'-monophospha	Nucleotide metabolome	1.506002537	0.465777066	0.749780452	1.643067895	0.0430234	0.17435193	0.06058304	0.02288414	0.123621	0.258233	0.139633	0.094837
Hypoxanthine	Nucleotide metabolome	-0.037891104	0.596263602	0.444552613	1.519397387	0.47215743	0.01246902	0.08535843	0.00591071	0.488009	0.057172	0.163331	0.044693
3'-Aenylic Acid	Nucleotide metabolome	1.392182494	0.599545275	0.44272121	1.267302783	0.0756399	0.1565078	0.16931641	0.03812101	0.169796	0.243914	0.251796	0.116857
5-Methyluridine	Nucleotide metabolome	0.772244296	0.763794199	1.568552482	1.231203344	0.01544519	0.03261419	0.00108558	0.0002229	0.070818	0.100405	0.015572	0.00739
5-Methylcytosine	Nucleotide metabolome	-0.522802704	-0.525288643	-0.436557512	-1.247869653	0.03693815	0.14560266	0.09640043	0.00117853	0.113717	0.235036	0.173843	0.014513
Guanosine Monophosphate	Nucleotide metabolome	-1.01085111	-2.375810208	-1.823182945	-1.709805764	0.01087734	0.00013579	0.00038042	0.00100876	0.060875	0.004093	0.007808	0.014459
Dihydrouracil	Nucleotide metabolome	-2.140363657	-1.374596288	-2.358028205	-1.926207632	0.00048212	0.00417724	0.00035052	0.0006581	0.01148	0.029515	0.007733	0.012332
B-Pseudouridine	Nucleotide metabolome	-2.895100616	-2.810705713	-2.23918617	-2.517156789	0.02662211	0.02760949	0.03716515	0.0315478	0.095923	0.094442	0.105511	0.106937
Cis-Aconitic Acid	Organic Acid And Its Derivatives	-1.120802486	-0.857782801	-0.875442375	-1.447042979	0.00407701	0.0154246	0.01187099	0.00130409	0.035352	0.065176	0.068219	0.015613
Citramalic Acid	Organic Acid And Its Derivatives	-3.548147156	-3.183793648	-3.326909404	-3.715308677	0.02789946	0.03079973	0.02950419	0.02689142	0.097342	0.099065	0.095782	0.100784
Malic Acid	Organic Acid And Its Derivatives	-3.206838386	-3.563824852	-3.227371977	-5.178816299	0.01140767	0.01007657	0.01143676	0.00755024	0.061459	0.0505	0.067342	0.052433
N1-Acetylspermine	Organic Acid And Its Derivatives	1.018200609	1.116510939	1.753830238	1.935242615	0.08698112	0.09454163	0.03883918	0.04390691	0.183606	0.185151	0.107306	0.122882
N-lactoyl-phenylalanine	Organic Acid And Its Derivatives	0.284114294	0.903020696	1.471939376	1.557708606	0.18099055	0.02899227	0.01866529	0.00114064	0.270857	0.095447	0.078104	0.014459
Mandelic Acid	Organic Acid And Its Derivatives	0.097443856	1.720011942	1.64699748	1.321684741	0.43315083	0.15277905	0.00444279	0.02881815	0.466875	0.242087	0.038018	0.101808
3-Hydroxy-3-Methyl Butyric Aci	Organic Acid And Its Derivatives	0.577803772	0.939790526	1.253056418	1.166968551	0.06686681	0.05853304	0.00717393	0.00034364	0.159224	0.144987	0.050091	0.008712
Phenyllactate (Pla)	Organic Acid And Its Derivatives	-0.069363859	0.558788547	1.136634978	1.141881586	0.37732298	0.07221966	0.01498654	0.00016516	0.423506	0.158914	0.072153	0.006596
4-Hydroxy-2-Oxoglutaric Acid	Organic Acid And Its Derivatives	-0.68396317	-0.910530846	-1.11941989	-1.305520833	0.15069856	0.07249327	0.04718325	0.02597414	0.245526	0.158914	0.119399	0.099267
Indoxylsulfuric acid	Organic Acid And Its Derivatives	-0.920873052	-1.457753371	-0.981937431	-1.483662536	0.0922302	0.03763704	0.14010809	0.00887305	0.187506	0.10887	0.224485	0.057079
Dl-2-Aminooctanoic Acid	Organic Acid And Its Derivatives	-1.244598672	-1.255631851	-1.282523616	-1.497514137	0.01349284	0.01360307	0.01267284	0.00732938	0.067488	0.059826	0.06962	0.052433
2-Aminoethanesulfinic Acid	Organic Acid And Its Derivatives	-1.280009315	-1.496471445	-1.144544201	-1.758349791	0.01853787	0.01176923	0.02491116	0.00773289	0.081247	0.055742	0.08729	0.052433
L-Methionine Sulfoximine	Organic Acid And Its Derivatives	-2.307100912	-2.509294467	-2.362623912	-2.665328985	0.02915224	0.02594397	0.02826969	0.0238819	0.098161	0.092163	0.093835	0.096197
2-Hydroxyisocaproic Acid	Organic Acid And Its Derivatives	-3.790464382	-3.166002691	-3.029558807	-2.805247046	0.03351073	0.03866299	0.04024942	0.04346848	0.108595	0.110356	0.109104	0.122452
Phenylpyruvic Acid	Organic Acid And Its Derivatives	-2.737370388	-2.69576858	-3.528189451	-3.247680964	0.04058986	0.04101646	0.03129708	0.03379101	0.119823	0.113306	0.097747	0.110333
Ergothioneine	Organic Acid And Its Derivatives	-3.448930308	-4.152856758	-3.89268884	-3.512533296	0.02541384	0.02195826	0.02297446	0.02503998	0.093766	0.082296	0.085196	0.098111
Carbamoyl phosphate	Organic Acid And Its Derivatives	-1.526632777	-2.042064967	-2.666074024	-3.666841326	0.00593383	0.00334522	0.00085863	0.00049831	0.042625	0.025746	0.013217	0.010227
Isonicotinic acid	Organic Acid And Its Derivatives	-3.879547296	-5.648610448	-4.955527493	-4.637430056	0.01503823	0.01206104	0.01276095	0.01323711	0.070451	0.055896	0.06962	0.070913
Acetaminophen Glucuronide	Carbohydrate metabolome	-0.230924481	0.196090345	1.149390556	1.676663834	0.30909692	0.3277224	0.06419961	0.03924157	0.37075	0.386982	0.141634	0.118274
L-Erythrulose	Carbohydrate metabolome	-0.076489985	0.154721829	0.988749157	1.020589037	0.39538228	0.39519967	0.02133869	0.00235425	0.437049	0.427967	0.081959	0.026017
Lactose	Carbohydrate metabolome	-2.23635777	-1.623656778	-2.314813049	-1.395327023	0.00765052	0.0158764	0.00717217	0.02311053	0.051521	0.066203	0.050091	0.094863
D-Glucoronic Acid	Carbohydrate metabolome	-2.443511932	-1.447618479	-1.312332069	-1.503869652	0.01093825	0.03814313	0.04009403	0.02970504	0.060875	0.109598	0.109104	0.104078
Gluconic Acid	Carbohydrate metabolome	-2.016004597	-1.887481838	-1.60319055	-1.532762172	0.01115806	0.01294232	0.02054185	0.02074689	0.060875	0.058717	0.080933	0.088319
L-Gulonic-γ-Lactone	Carbohydrate metabolome	-1.393766355	-1.511403277	-1.550709624	-1.700103805	0.02379292	0.01921342	0.01821003	0.01475217	0.09156	0.075972	0.077626	0.070913
D-Xylulose 5-phosphate	Carbohydrate metabolome	-1.110634663	-1.931070057	-1.509001431	-1.81924148	0.00058612	9.4071E-07	1.3602E-06	9.2166E-07	0.01148	0.000135	0.000195	0.000188
L-Rhamnose	Carbohydrate metabolome	-0.402516994	-0.619246806	-1.4398494	-2.016649444	0.2735613	0.19124387	0.02474441	0.0087337	0.344751	0.276598	0.08729	0.057034
D-Mannitol	Carbohydrate metabolome	-4.200787431	-4.48231841	-3.579845329	-2.116327027	0.00154245	0.00145919	0.00185159	0.01029908	0.018467	0.013976	0.021001	0.063413
L-Fucose	Carbohydrate metabolome	-0.630581712	-1.155422959	-1.929328553	-3.013742894	0.19591725	0.07263608	0.01785314	0.00778594	0.283357	0.158914	0.076947	0.052433
1,5-Anhydro-D-Glucitol	Carbohydrate metabolome	-1.526155949	-1.569348282	-2.257159908	-3.086229903	0.04243338	0.04086474	0.0207695	0.01268936	0.122744	0.113306	0.080933	0.070913
Scyllo inositol	Alcohol	-0.05009625	0.079143822	1.155304021	1.706850263	0.46760834	0.44392587	0.0438122	0.03673148	0.485528	0.461041	0.113753	0.116212
2-Methyl-5-nitroimidazole-1-eth	Alcohol	2.169949698	1.669196068	1.664315059	1.410794066	0.03545064	0.00860949	0.02647556	0.01455014	0.111527	0.046384	0.090064	0.070913
Norambreinolide	Lactone	-0.714199887	-0.744741143	-0.701920366	-1.166068593	0.02549419	0.0363593	0.03197217	0.00287439	0.093766	0.107335	0.098959	0.028871
1-Aminopropan-2-ol	Alcohol	-1.491288496	-1.788290069	-1.299935025	-2.151934585	0.00719931	0.00434183	0.0326038	0.00070028	0.05058	0.030183	0.098959	0.012576
Isoxanthopterin	Pteridines and derivatives	-2.531334118	-3.01890695	-2.600038606	-2.676187792	0.02866832	0.02297173	0.02753016	0.02651252	0.097995	0.085352	0.092699	0.100236
Cyclohexylamine	Amines	-0.553781165	-2.934826851	-2.727100227	-2.743376041	0.22517876	0.00034456	0.00041044	0.00039795	0.311064	0.00594	0.008041	0.009529
Putrescine	Polyamine	-2.844967201	-2.659468757	-2.703403291	-2.852560167	4.0929E-08	4.4946E-08	4.0817E-08	5.0504E-08	1.76E-05	9.68E-06	8.79E-06	2.18E-05
Serotonin	Indole And Its Derivatives	-2.822462604	-3.883906002	-3.800300115	-2.874339687	0.02887563	0.02061719	0.02084342	0.02812473	0.097995	0.078637	0.080933	0.101808
Neopterin	Pteridines and derivatives	-2.787512544	-3.036663962	-2.816184942	-3.22401401	0.00018005	0.00017464	0.00016523	0.00015409	0.007268	0.004377	0.006474	0.006596
Indole-3-acetamide	Indole And Its Derivatives	-3.731376522	-3.921986967	-3.106105716	-3.517754908	0.00506415	0.00480614	0.00641108	0.00541906	0.038595	0.032063	0.049438	0.042466
2-Picoline	Pyridine And Pyridine Derivatives	-2.362221327	-2.820975029	-3.261610435	-3.574343308	0.02721545	0.02003866	0.01667657	0.01515249	0.096941	0.077183	0.074333	0.07168
Sn-Glycero-3-Phosphocholine	Cholines	-4.973711277	-5.332667589	-5.307573787	-4.824307576	0.03674479	0.03587996	0.03593561	0.03718428	0.113717	0.10665	0.103255	0.116212
δ-Valerolactam	Pyridine And Pyridine Derivatives	-0.639148423	-4.157411758	-4.239463719	-5.334100861	0.22300092	0.00054739	0.00054706	0.00046065	0.310781	0.007864	0.009824	0.009927
3-Indolepropionic Acid	Indole And Its Derivatives	-4.929557423	-7.226453584	-8.662062132	-6.674774772	0.00032426	0.00028214	0.00027481	0.00028835	0.008735	0.005287	0.007652	0.007966
Estrone	Hormones	-0.145457338	0.019546309	0.857122614	1.206205783	0.35634342	0.48458731	0.10557413	0.04553762	0.406307	0.490275	0.185875	0.12422
L-Thyroxine	Hormones	-0.188117556	-0.40077217	-0.64945803	-1.007003814	0.02772902	0.00356096	1.992E-05	1.6393E-05	0.097342	0.026305	0.001429	0.001767
octadecanedioate (C18)	Hydrocarbon derivative	-0.118078167	0.393535189	1.354421113	-1.109906788	0.40389557	0.29718643	0.17598932	0.01295613	0.440837	0.361829	0.256255	0.070913
3,3',5-Triiodo-L-Thyronine	Hormones	-1.279338406	-1.679313098	-1.845531732	-1.492671405	9.3271E-08	3.319E-09	1.6692E-10	1.309E-06	2.01E-05	1.43E-06	7.2E-08	0.000188
Orotic Acid	CoOthersEnzyme Factor & vitamin	-1.610007577	-1.353042967	-1.629173256	-1.908832552	0.01182114	0.01812697	0.01141437	0.00736645	0.062208	0.073016	0.067342	0.052433
Glycyrrhetinic acid	Terpenoid	-1.349908869	0.243520646	-1.613806534	-2.207272021	0.08882127	0.42117632	0.07064852	0.04458258	0.184048	0.443396	0.147099	0.123174
1,2-Dichloroethane	Hydrocarbon derivative	-5.187816921	-5.223361669	-5.246237585	-5.295740786	0.04374182	0.04364953	0.0435911	0.04346904	0.124031	0.119069	0.113753	0.122452
Allantoin	others	-4.395176642	-4.186285446	-4.03701915	-4.969576518	0.00022164	0.00023191	0.00024009	0.00020487	0.007268	0.00476	0.007427	0.007358

Table S5. Metabolomics data of S vs H and M vs H

		**Log_2 FC**				***P* value**				**FDR**			
Compounds	Class	ST1/H	ST2/H	MT1/H	MT2/H	ST1/H-P	ST2/H-P	MT1/H-P	MT2/H-P	ST1/H-FDR	ST2/H-FDR	MT1/H-FDR	MT2/H-FDR
Phe-Phe	Amino Acid metabolome	-1.05906834	-1.22583751	-1.68646136	-1.11816052	0.004679	0.0014214	0.00019426	0.00181709	0.04358824	0.0139233	0.00272404	0.01118808
L-Cystathionine	Amino Acid metabolome	-1.75346445	-2.40892785	-2.40260195	-2.43224694	0.00173196	0.00069417	0.00065813	0.0006843	0.02132785	0.0085482	0.00578884	0.00508508
L-Dopa	Amino Acid metabolome	-2.29427823	-2.44310536	-2.31547106	-2.39848049	0.00220982	0.00190194	0.00214842	0.00199468	0.02645649	0.01821635	0.01341986	0.01206046
3-Hydroxykynurenine	Amino Acid metabolome	-1.79186548	-1.81585898	-1.22318025	-1.53950307	0.01956506	0.0188518	0.0442881	0.02732111	0.09849111	0.08149999	0.10458166	0.06967693
N-Alpha-Acetyl-L-Asparagine	Amino Acid metabolome	-2.55805256	-2.84708047	-3.13324874	-2.90126155	0.00064116	0.00053991	0.00045615	0.0004967	0.013159	0.00810579	0.00436889	0.00428153
N-Amidino-L-Aspartate	Amino Acid metabolome	-2.42070182	-2.44171792	-2.93976997	-1.89162518	0.01865745	0.01838336	0.01402385	0.02852099	0.09688387	0.08149999	0.04835422	0.07146829
N-Acetylneuraminic Acid	Amino Acid metabolome	-2.39427202	-2.81504141	-2.41293577	-2.3685697	0.00131501	0.00091455	0.0012841	0.00134177	0.01771153	0.01065329	0.00864764	0.00876215
L-Alanyl-L-Lysine	Amino Acid metabolome	-2.10859967	-2.80481191	-3.13307108	-2.75343848	0.02138691	0.01346366	0.01162127	0.01382661	0.10241953	0.06826868	0.04252109	0.04584052
N-Acetyl-L-Leucine	Amino Acid metabolome	-2.84733916	-3.00382764	-2.74393994	-3.22375948	3.0537E-05	2.8179E-05	3.2505E-05	2.6904E-05	0.00162131	0.00151813	0.00093397	0.00063699
L-Cystine	Amino Acid metabolome	-1.45739381	-1.2128957	-1.54212771	-1.09266731	0.04918087	0.06831089	0.04440474	0.08064969	0.15015933	0.16200898	0.10458166	0.1455814
Sarcosine	Amino Acid metabolome	-3.71742281	-3.61034573	-3.68508642	-3.08786364	5.7408E-05	6.0223E-05	5.8428E-05	6.106E-05	0.00218058	0.00196619	0.0013254	0.00119622
L-Aspartic Acid	Amino Acid metabolome	-5.09457755	-4.99466674	-4.64914552	-4.92532183	0.00053763	0.0005454	0.00057725	0.00055033	0.0115859	0.00810579	0.00529347	0.00440516
N-Glycyl-L-Leucine	Amino Acid metabolome	-3.58519868	-3.45770462	-3.09782118	-2.14804422	0.03196754	0.03288801	0.03624452	0.0547302	0.12517447	0.11074011	0.09029704	0.11167288
Nα-Acetyl-L-Arginine	Amino Acid metabolome	-4.14902549	-3.92033453	-3.95763022	-3.96718522	0.00105855	0.0011271	0.00111502	0.0011132	0.01573226	0.01180027	0.00809517	0.00773856
O-Acetyl-L-serine	Amino Acid metabolome	-4.64275323	-4.77679881	-4.43182731	-4.37768611	0.00017333	0.00017055	0.00018254	0.00018418	0.00462904	0.00411844	0.00272404	0.00212358
5'-Deoxy-5'-(Methylthio) Adeno	Nucleotide metabolome	-4.33756112	-4.51862491	-5.26895372	-5.09975755	6.4947E-05	6.3867E-05	5.6128E-05	5.7486E-05	0.00218058	0.00196619	0.0013254	0.00117983
Uridine triphosphate (UTP)	Nucleotide metabolome	-2.04084714	-1.57538364	-2.17406863	-1.99955254	0.01340991	0.02928856	0.01021172	0.01255587	0.07888482	0.10412017	0.03860747	0.04364176
Inosine	Nucleotide metabolome	-2.15733591	-1.86936199	-2.55037762	-2.57257078	0.03076683	0.03871004	0.0241337	0.02381901	0.12345615	0.11936908	0.07027749	0.06303615
Dihydrouracil	Nucleotide metabolome	-1.9166068	-0.85149498	-1.71069707	-1.80430668	0.00067919	0.08039193	0.00100123	0.00085537	0.01330601	0.1756395	0.00799132	0.00604369
B-Pseudouridine	Nucleotide metabolome	-2.87588338	-2.74390295	-3.10336524	-2.96865984	0.0268563	0.02840292	0.02464983	0.02586435	0.11459531	0.10412017	0.07027749	0.0667517
Succinic Acid	Organic Acid And Its Derivatives	-1.26588835	-1.31492814	-1.30385224	-1.1569683	3.3856E-05	0.00033037	1.0842E-05	3.4707E-05	0.00162131	0.00593295	0.00046729	0.00074794
Citramalic Acid	Organic Acid And Its Derivatives	-4.12579939	-4.24022408	-3.82168539	-3.73400652	0.02497068	0.02454446	0.02632056	0.02678008	0.11095221	0.09361649	0.07271898	0.06870365
Malic Acid	Organic Acid And Its Derivatives	-4.79582888	-4.29492432	-4.43537353	-4.21496517	0.00787589	0.00849239	0.00829052	0.00861254	0.05815933	0.05642869	0.03403061	0.03392794
(3-Methoxy-4-hydroxyphenyl)et	Organic Acid And Its Derivatives	-1.18864341	-1.22015497	-1.69220885	-1.2681763	0.00085867	0.00057151	3.029E-05	0.00226143	0.01382479	0.00821069	0.00093249	0.01277197
3-Methoxy-4-Hydroxyphenyleth	Organic Acid And Its Derivatives	-3.1816549	-3.04456093	-3.22145343	-1.01644672	2.6286E-08	7.498E-08	2.6824E-09	0.05291625	1.1329E-05	3.2316E-05	6.9822E-07	0.10964858
Chlorogenic Acid	Organic Acid And Its Derivatives	-2.45077631	-0.3370378	-2.15271448	-1.80268912	0.03345197	0.37568243	0.04019139	0.05151056	0.12627251	0.4294937	0.09898564	0.10777209
N'-Formylkynurenine	Organic Acid And Its Derivatives	-2.15871708	-2.34291063	-3.20625358	-1.60315939	0.00447591	0.00406984	0.00189247	0.01466406	0.04358824	0.03077373	0.01235616	0.04600378
4-Hydroxy-2-Oxoglutaric Acid	Organic Acid And Its Derivatives	-1.72476775	-1.87048899	-2.129502	-1.98912786	0.01353819	0.01173491	0.00823174	0.00997846	0.07888482	0.06702049	0.03403061	0.03786242
2-Aminoethanesulfinic Acid	Organic Acid And Its Derivatives	-2.01329404	-1.95467249	-1.74297146	-1.40494235	0.00542378	0.00585967	0.00766507	0.01345374	0.04770712	0.04140194	0.03270937	0.04565798
L-Methionine Sulfoximine	Organic Acid And Its Derivatives	-2.99568536	-3.04153693	-3.20484907	-3.25765697	0.02064383	0.0202872	0.01913397	0.01880763	0.09997181	0.08327411	0.05932907	0.05440329
2-Hydroxyisocaproic Acid	Organic Acid And Its Derivatives	-3.65182989	-3.68695653	-3.65881873	-3.90715702	0.03440263	0.0341794	0.0343559	0.03281746	0.12673106	0.11196622	0.08659295	0.07901857
Phenylpyruvic Acid	Organic Acid And Its Derivatives	-4.88666167	-4.97877429	-5.57541519	-6.02015642	0.02556932	0.02536695	0.02434985	0.02383966	0.11245282	0.09507092	0.07027749	0.06303615
Ergothioneine	Organic Acid And Its Derivatives	-4.14382146	-3.93661367	-4.47122515	-4.52810803	0.02196949	0.02277318	0.02096201	0.02081401	0.10405328	0.08911009	0.06362413	0.0579658
Carbamoyl phosphate	Organic Acid And Its Derivatives	-2.95772748	-3.14692934	-2.6558513	-1.92832878	0.00072114	0.00065168	0.00091588	0.00204818	0.0135135	0.00851135	0.00744804	0.01206046
Isonicotinic acid	Organic Acid And Its Derivatives	-4.46445661	-4.67356028	-4.85806258	-3.88968644	0.01354403	0.01316956	0.01288277	0.01502184	0.07888482	0.06757238	0.04514206	0.04657852
Xylose	Carbohydrate metabolome	-1.0535778	-0.93966636	-1.90435299	-1.5999677	0.04947245	0.0443139	0.00695623	0.0107245	0.15015933	0.12675593	0.03090861	0.03984708
Lactose	Carbohydrate metabolome	-2.33864995	-1.59596119	-2.22554951	-1.74121794	0.00698241	0.01700581	0.00799764	0.01725555	0.05373962	0.07881189	0.03346584	0.05200797
D-Glucoronic Acid	Carbohydrate metabolome	-2.6799046	-2.50784307	-3.24887496	-2.81334557	0.0093604	0.01045886	0.00705644	0.0086591	0.06613662	0.06533001	0.03103394	0.03392794
Gluconic Acid	Carbohydrate metabolome	-2.85259884	-1.95587227	-2.1851394	-2.15167591	0.0057068	0.01211333	0.00940973	0.01006128	0.04861878	0.06705363	0.03653687	0.03786242
L-Gulonic-γ-Lactone	Carbohydrate metabolome	-1.59401273	-1.74174734	-1.67023036	-1.71872069	0.01692661	0.01388157	0.01524971	0.01430647	0.09254256	0.06956925	0.05123981	0.04600378
D-Xylulose 5-phosphate	Carbohydrate metabolome	-1.444644	-1.05160009	-1.42366898	-0.96677257	2.329E-06	4.0508E-05	2.6262E-06	2.6107E-05	0.00025096	0.00174591	0.00018865	0.00063699
L-Rhamnose	Carbohydrate metabolome	-2.93019633	-3.48254758	-2.80808161	-4.14458569	0.00404843	0.00312847	0.00436184	0.00256686	0.04358824	0.02544098	0.02332911	0.01400401
D-Mannitol	Carbohydrate metabolome	-5.40153652	-4.78256707	-5.5438931	-5.13857399	0.00128126	0.00138758	0.00126378	0.00132013	0.01771153	0.01390805	0.00864585	0.00875348
L-Fucose	Carbohydrate metabolome	-3.59766842	-4.05648469	-3.53633348	-5.07375555	0.00615817	0.00543627	0.00628495	0.00460585	0.0491513	0.03905057	0.02943371	0.02194888
1,5-Anhydro-D-Glucitol	Carbohydrate metabolome	-3.4542073	-3.12839242	-3.06713628	-5.04604577	0.01113901	0.0126342	0.01279778	0.00814965	0.0750143	0.06705363	0.04514206	0.03252316
2-Methyl-5-nitroimidazole-1-eth	Alcohol	1.733934443	1.678609625	1.360681696	1.968402856	0.00463457	0.00195199	4.4946E-05	4.6805E-06	0.04358824	0.01827065	0.00113835	0.0002951
Pulegone	Ketones	-5.30640569	-5.3068221	-4.5834145	-4.92088401	0.01725032	0.01724931	0.0184869	0.01782403	0.09293609	0.07908994	0.0581595	0.05212427
4-Pyridoxic Acid	Pyridine And Pyridine Derivatives	-1.59548	-1.5691076	-1.34550914	-1.4972066	0.00015571	0.00018943	0.00031723	0.00016468	0.00447415	0.00420137	0.00359807	0.00202794
Methyl Indole-3-Acetate	Indole And Its Derivatives	-1.92095722	-1.0571598	-2.07051003	-1.14229015	0.00135916	0.011818	0.00112108	0.00745495	0.01775148	0.06702049	0.00809517	0.0306008
Dopamine	Polyamine	-1.42453376	-1.50217662	-1.72466544	-1.53733559	0.00082577	0.00067939	0.00042525	0.00063806	0.01382479	0.0085482	0.00421046	0.00482464
Isoxanthopterin	Pteridines and derivatives	-2.08614335	-2.46212989	-3.61471926	-2.21400031	0.0407124	0.02995569	0.01909292	0.03573903	0.13708629	0.10412017	0.05932907	0.08417225
Putrescine	Polyamine	-4.27094928	-4.24835776	-4.16225395	-4.25549149	2.1818E-07	2.1832E-07	2.1041E-07	2.0299E-07	4.6979E-05	4.6979E-05	0.00003017	4.3747E-05
Serotonin	Indole And Its Derivatives	-4.08890129	-3.67607239	-3.02019645	-2.17332745	0.01969912	0.02137845	0.02596244	0.03900307	0.09849111	0.08531585	0.07219232	0.08941662
Neopterin	Pteridines and derivatives	-3.48082292	-3.56946561	-3.72712733	-3.57962737	0.00013736	0.00013101	0.00012246	0.00013141	0.00422888	0.00352895	0.00219921	0.00168278
Indole-3-acetamide	Indole And Its Derivatives	-3.94544463	-4.48006986	-4.8249362	-4.98632791	0.004777	0.00426964	0.00404978	0.00396659	0.04358824	0.03172783	0.02270503	0.02011294
2-Picoline	Pyridine And Pyridine Derivatives	-4.34103671	-4.4063533	-3.9880455	-3.95488989	0.01288622	0.0127573	0.01372313	0.01381893	0.07888482	0.06705363	0.04769893	0.04584052
Sn-Glycero-3-Phosphocholine	Cholines	-4.43189705	-3.90612053	-3.98442995	-3.41890967	0.03858826	0.04133331	0.04084202	0.0452143	0.13305231	0.12371288	0.10001653	0.09823542
Allantoin	others	-5.26088907	-5.36381033	-5.19390846	-4.53725796	0.00019772	0.00019496	0.00019908	0.00022095	0.0047342	0.00420137	0.00272404	0.00244177
Dethiobiotin	Others	-2.09310064	-2.67871259	-2.02838744	-2.49109972	4.2278E-05	6.1391E-06	2.2188E-05	5.4774E-06	0.00182219	0.00052919	0.00073563	0.0002951
Triethylamine	Hydrocarbon derivative	-2.53705973	-2.34902806	-2.01939208	-1.99688631	0.00259984	0.00307956	0.00438436	0.00451277	0.03028461	0.02544098	0.02332911	0.02185397
Orotic Acid	CoOthersEnzyme Factor & vitamin	-2.09136889	-2.18933543	-2.27894077	-2.42635822	0.00589772	0.00532798	0.00487922	0.00427696	0.04861878	0.03892137	0.02564567	0.02101238
1,2-Dichloroethane	Hydrocarbon derivative	-5.25167899	-5.21395328	-5.30243024	-5.33580582	0.04357734	0.04367309	0.04345266	0.04337323	0.14337277	0.12632956	0.10454795	0.09636011

Table S6. KEGG enrichment analysis of DEMs shared by F vs H, S vs H and M vs H

#KEGG n	29	KEGG N	231				
#Pathway	ko ID	Unique co	compound	Uni all	compound	all	
Metabolic pathways	ko01100	26	202	29	231	MEDN240:L-Gulonic-Γ-La	C01040+C00169+C00507+C05570+C00355+C06752+C00243+C00429+C00149+C00392+C00166+C00491+C02693+C00295+C00231+C00075+C00979+C01127+C00257+C01019+C00213+C00049+C00134+C02067+C00780+C00
Cysteine and methionine metabolisn	ko00270	3	18	29	231	MEDN616:O-Acetyl-L-seri	C00979+C00049+C00491
ABC transporters	ko02010	5	43	29	231	MEDP127:Putrescine;MEL	C00134+C00243+C00049+C00392+C00491
Protein digestion and absorption	ko04974	3	26	29	231	MEDP127:Putrescine;MEL	C00134+C00049+C00491
Glycine, serine and threonine metab	ko00260	2	19	29	231	MEDN009:L-Aspartic Acid	C00049+C00213
Arginine and proline metabolism	ko00330	2	18	29	231	MEDP404:Sarcosine;MEDI	C00213+C00134
Arginine biosynthesis	ko00220	2	12	29	231	MEDN615:Carbamoyl pho:	C00169+C00049
Alanine, aspartate and glutamate me	ko00250	2	11	29	231	MEDN009:L-Aspartic Acid	C00049+C00169
Histidine metabolism	ko00340	2	8	29	231	MEDP437:Ergothioneine;M	C05570+C00049
beta-Alanine metabolism	ko00410	2	9	29	231	MEDN009:L-Aspartic Acid	C00049+C00429
Pantothenate and CoA biosynthesis	ko00770	2	10	29	231	MEDN009:L-Aspartic Acid	C00049+C00429
Carbon metabolism	ko01200	6	18	29	231	MEDN009:L-Aspartic Acid	C00049+C00149+C00231+C00979+C00257+C00169
2-Oxocarboxylic acid metabolism	ko01210	2	23	29	231	MEDN339:Phenylpyruvic A	C00166+C00049
Biosynthesis of amino acids	ko01230	5	49	29	231	MEDN615:Carbamoyl pho:	C00169+C00049+C00231+C00979+C00166
Neuroactive ligand-receptor interact	ko04080	3	13	29	231	MEDN009:L-Aspartic Acid	C00049+C00075+C00780
Central carbon metabolism in cance	ko05230	2	25	29	231	MEDN009:L-Aspartic Acid	C00049+C00149
Glyoxylate and dicarboxylate metab	ko00630	2	12	29	231	MEDN200:L-Malic Acid;M	C00149+C01127
Taste transduction	ko04742	2	11	29	231	MEDP081:Serotonin;MED	C00780+C00149
Pyrimidine metabolism	ko00240	5	21	29	231	MEDN244:Orotic Acid;ME	C00295+C00169+C02067+C00075+C00429
Pentose phosphate pathway	ko00030	2	6	29	231	MEDP236:Gluconic Acid;M	C00257+C00231
Ascorbate and aldarate metabolism	ko00053	2	6	29	231	MEDN240:L-Gulonic-Γ-La	C01040+C00231
Fructose and mannose metabolism	ko00051	3	5	29	231	MEDN232:L-Rhamnose;M	C00507+C01019+C00392
Tryptophan metabolism	ko00380	2	17	29	231	MEDP081:Serotonin;MED	C00780+C02693

Table S7. KEGG enrichment analysis of DEMs unique to F vs H

#KEGG_n	13	KEGG_N	231			
#Pathway	ko_ID	Unique cor	compound	Uni_all	compound_all	
Metabolic	ko01100	13	202	13	231	MEDP036: C02237+C00144+C02376+C01829+C00468+C00262+C03740+C06153+C00362+C01367+C00417+C00097+C02465
Glutathione	ko00480	2	12	13	231	MEDP028: C03740+C00097
Purine meta	ko00230	4	16	13	231	MEDN163 C00262+C01367+C00362+C00144
Tyrosine m	ko00350	2	19	13	231	MEDN179 C01829+C02465
Neuroactiv	ko04080	2	13	13	231	MEDN179 C01829+C02465
Thyroid ho	ko04918	2	6	13	231	MEDN179 C01829+C02465
Thyroid ho	ko04919	2	3	13	231	MEDN179 C01829+C02465
Bile secreti	ko04976	2	11	13	231	MEDP184: C02465+C01829
Autoimmu	ko05320	2	2	13	231	MEDN179 C01829+C02465

Table S8. Lipidomic data of F vs H

		Log_2 FC				P value				FDR			
Compounds	Class	F1/H	F2/H	F3/H	F4/H	F1/H-P	F2/H-P	F3/H-P	F4/H-P	F1/H-FDR	F2/H-FDR	F3/H-FDR	F4/H-FDR
3-Hydroxy-tetradecenoyl- carnit	Fatty Acyls	0.732891	1.058621	0.936478	1.322227	0.035259	0.006774	0.003624	0.003958	0.114833	0.078384	0.043978	0.038373
CE(18:0)	Steroids and steroid derivatives	-0.65621	-0.78053	-0.9131	-1.32363	0.017791	0.008787	0.003213	0.000636	0.085116	0.085183	0.043978	0.0148
Cer(d18:0/18:0)	Sphingolipids	1.837063	1.945024	1.959347	1.863655	0.000374	0.001599	0.011023	0.002918	0.012851	0.035426	0.074975	0.0343
DG(16:0/20:2/0:0)	Glycerolipids	3.156343	3.856241	3.285843	4.154154	0.02941	0.024028	0.01167	0.014896	0.026971	0.042183	0.030161	0.012065
DG(14:1/22:3/0:0)	Glycerolipids	1.757707	1.36161	1.832577	2.79635	0.001871	0.000903	0.001274	0.004844	0.043743	0.108438	0.043978	0.069247
DG(16:0/16:1/0:0)	Glycerolipids	1.881072	2.126148	1.704713	2.106992	0.005756	0.005341	0.005894	0.000544	0.049522	0.073197	0.053874	0.014093
DG(16:1/18:3/0:0)	Glycerolipids	1.941991	1.465783	1.300218	2.034405	0.00183	0.002259	0.001397	0.000398	0.142565	0.125179	0.197948	0.07012
DG(16:1/20:2/0:0)	Glycerolipids	1.072046	1.264273	1.538108	1.928656	0.034651	0.017008	0.014229	0.0011	0.114833	0.11711	0.084167	0.01919
DG(18:2/20:1/0:0)	Glycerolipids	0.607559	0.974156	0.852753	1.760549	0.165856	0.057757	0.087492	0.009319	0.265098	0.182381	0.201721	0.057566
DG(18:1/20:5/0:0)	Glycerolipids	0.5807	0.903268	1.303825	1.638523	0.168058	0.056314	0.039592	0.007025	0.266602	0.18114	0.143187	0.050553
DG(14:1/22:2/0:0)	Glycerolipids	0.932721	0.848304	1.141428	1.60097	0.071348	0.066491	0.022099	0.010441	0.161692	0.192576	0.110178	0.062994
DG(16:0/20:1/0:0)	Glycerolipids	0.315536	0.27446	0.584002	1.26688	0.325896	0.341448	0.185707	0.036971	0.392623	0.424325	0.295454	0.120123
DG(16:0/20:3/0:0)	Glycerolipids	-0.11785	0.567547	3.479046	1.129547	0.427346	0.170898	0.148393	0.045957	0.464622	0.296733	0.27014	0.133658
DG(18:1/20:0/0:0)	Glycerolipids	-0.0374	-0.09887	0.028185	1.027809	0.453178	0.371021	0.470814	0.041666	0.475494	0.439493	0.483276	0.126946
DG(18:2/20:4/0:0)	Glycerolipids	0.460721	0.585942	0.673665	1.017436	0.046836	0.035854	0.011214	0.029214	0.132893	0.143006	0.075262	0.107649
(±)12-HETE	Eicosanoid	4.253631	4.206264	3.131	4.833536	0.057189	0.020265	0.08366	0.013177	0.046413	0.135231	0.074975	0.155493
PGF2α	Eicosanoid	2.559641	1.964423	4.061743	4.120179	0.004386	0.014321	0.003287	0.012599	0.274034	0.271702	0.283387	0.203016
5-iso PGF2VI	Eicosanoid	0.441651	0.942004	1.046965	3.611075	0.000185	0.00407	0.00081	0.002911	0.341491	0.226624	0.191782	0.163774
FFA(18:3)	Fatty acids	0.725108	0.713111	1.025713	2.444395	0.000574	0.000413	0.000196	0.000122	0.221987	0.198316	0.123757	0.153594
FFA(22:4)	Fatty acids	1.052038	1.109519	0.899904	1.148244	0.000766	0.002296	0.000304	0.030227	0.016211	0.042183	0.011776	0.107649
FFA(22:2)	Fatty acids	0.677078	1.178435	0.971017	1.096585	0.051758	0.056992	0.024603	0.015305	0.136633	0.181646	0.114488	0.073169
FFA(22:0)	Fatty acids	-1.4544	-2.23349	-2.06996	-1.76132	0.02937	0.008699	0.010351	0.014545	0.112175	0.085183	0.074384	0.073169
Oleate	Fatty acids	0.062306	-0.39327	1.544085	-1.27721	0.468427	0.298216	0.179695	0.013633	0.481535	0.397425	0.290341	0.070392
Hexadecanedioic acid	Fatty acids	1.204368	1.825559	1.045968	1.241864	0.00445	0.022625	0.014219	0.00439	0.043743	0.129106	0.084167	0.040859
Farnesene	Fatty acids	-2.45454	-2.26485	-2.09316	-2.11914	0.021206	0.099354	0.040713	0.01101	0.093595	0.130692	0.119472	0.103014
LPA(18:1/0:0)	Glycerophospholipids	1.772428	2.03575	1.915876	1.460178	0.090353	0.063735	0.029694	0.022971	0.180189	0.19093	0.125801	0.09322
LPC(12:0/0:0)	Glycerophospholipids	1.988682	2.371117	1.801665	1.842264	0.000744	0.003459	5.63E-06	1.13E-05	0.016211	0.054888	0.000627	0.001443
LPC(O-20:1/0:0)	Glycerophospholipids	-1.13589	-1.03422	-1.02353	-1.00529	0.001511	0.001595	0.000814	0.0009	0.023444	0.035426	0.022724	0.017457
LPE(0:0/20:2)	Glycerophospholipids	3.445042	3.841904	3.703691	3.978356	0.006102	0.003271	0.004961	0.000115	0.008841	0.062912	0.022724	0.0343
LPE(0:0/16:1)	Glycerophospholipids	-0.07068	0.757932	1.073369	1.106595	0.367639	0.097695	0.012512	0.022061	0.419987	0.228065	0.081588	0.091327
LPE(0:0/24:6)	Glycerophospholipids	-0.66718	-0.76482	-1.42669	-1.45359	0.02307	0.064546	0.000168	0.000182	0.09819	0.192533	0.008401	0.007048
(±)12-HEPE [(±)-12-hydroxy-5Z	Oxidized lipid	3.282307	2.116852	2.978332	3.340256	0.012684	0.00107	0.012782	0.008109	0.112175	0.143055	0.181135	0.130979
(±)12-HETE [(±)12-hydroxy-5Z	Oxidized lipid	4.427983	2.696736	2.907706	2.973186	0.000387	0.035031	0.004601	0.01084	0.171824	0.179255	0.225155	0.125467
(±)5-HETE [(±)5-hydroxy-6E,8Z	Oxidized lipid	1.822193	1.731564	2.142326	1.354389	0.053806	0.026535	0.092794	0.03204	0.138395	0.134432	0.207302	0.110711
PC(18:2/20:4)	Glycerophospholipids	-0.78523	-0.74641	-0.95072	-1.00767	0.000684	0.027987	0.00076	5.01E-06	0.016211	0.135231	0.022724	0.001443
PC(20:2/22:6)	Glycerophospholipids	-0.34713	-0.60314	-0.78538	-1.31232	0.109504	0.015342	0.035223	0.000113	0.207701	0.110402	0.139395	0.005671
PC(18:3/14:1)	Glycerophospholipids	-1.00139	-0.50561	-1.45072	-1.34596	0.005548	0.084355	0.000103	0.000236	0.049522	0.212563	0.00599	0.008666
PC(18:0/20:1)	Glycerophospholipids	-0.4436	-1.12383	-1.36652	-1.51313	0.22801	0.058529	0.039548	0.032651	0.318005	0.182381	0.143187	0.112139
PC(18:0/20:3)	Glycerophospholipids	-2.57687	-1.18011	-1.71535	-1.57176	6.98E-05	0.00714	0.000415	0.000609	0.006092	0.078384	0.015228	0.014662
PC(O-18:2/18:1)	Glycerophospholipids	-0.9777	-0.95595	-0.96387	-1.13392	0.00296	0.006123	0.003426	0.00221	0.033323	0.076855	0.043978	0.029102
PC(O-20:2/22:1)	Glycerophospholipids	-1.47469	-2.09499	-2.08116	-2.99846	0.058423	0.049075	0.035413	0.037923	0.326708	0.321543	0.309631	0.254751
PE(18:1/16:1)	Glycerophospholipids	0.85907	0.896259	1.022551	1.713802	0.029884	0.022072	0.009866	0.001353	0.112654	0.129106	0.072492	0.022486
PE(16:0/20:3)	Glycerophospholipids	0.345407	0.691279	1.102432	1.447521	0.125932	0.030966	0.01512	0.007657	0.223097	0.140033	0.086508	0.051893
PE(18:3/16:0)	Glycerophospholipids	0.481505	1.048007	1.280195	1.403102	0.05584	0.028092	0.00054	0.001459	0.141219	0.135231	0.018857	0.023678
PE(20:2/16:0)	Glycerophospholipids	0.886649	1.029766	0.646767	1.328299	0.00926	0.039204	0.05387	0.025245	0.065953	0.148714	0.162177	0.09931
PE(18:1/18:1)	Glycerophospholipids	0.051414	0.595945	0.706182	1.274643	0.432166	0.130439	0.020859	0.002655	0.466953	0.256277	0.106275	0.033094
PE(16:1/16:0)	Glycerophospholipids	0.646717	1.001911	0.841791	1.254601	0.034365	0.014165	0.024995	0.020742	0.114833	0.108438	0.114662	0.089406
PE(18:1/18:2)	Glycerophospholipids	0.308513	0.834889	0.842207	1.216899	0.118317	0.134253	0.010444	0.002766	0.217254	0.260302	0.074384	0.033871
PE(18:1/16:0)	Glycerophospholipids	0.702429	0.973992	0.941321	1.174923	0.008155	0.005795	0.014999	0.001881	0.059916	0.074905	0.086508	0.027942
PE(18:2/16:0)	Glycerophospholipids	0.856155	1.135298	1.083812	1.155865	0.002932	0.034633	0.011029	0.002504	0.033323	0.142199	0.074975	0.03237
PE(18:1/20:4)	Glycerophospholipids	0.602911	0.785835	0.915018	1.144188	0.00199	0.031528	0.020497	0.005813	0.026971	0.140033	0.105465	0.046728
PE(22:6/20:1)	Glycerophospholipids	1.104173	1.071248	0.733964	1.13255	0.009958	0.00137	0.052794	0.005557	0.069508	0.034544	0.162121	0.046176
PE(18:2/14:0)	Glycerophospholipids	0.22172	0.855868	1.058296	1.1133	0.314543	0.116606	0.003648	0.003145	0.387215	0.241517	0.043978	0.0343
PE(14:0/22:6)	Glycerophospholipids	0.684661	1.148661	1.379301	1.071759	0.055355	0.05452	0.007193	0.00542	0.140888	0.179255	0.061226	0.045583
PE(18:1/18:0)	Glycerophospholipids	0.247405	0.584922	0.709438	1.052673	0.192325	0.12131	0.035315	0.014822	0.290858	0.247587	0.139395	0.073169
PE(16:1/18:0)	Glycerophospholipids	0.340382	0.808883	0.727173	1.021532	0.07113	0.010672	0.005481	0.002107	0.161692	0.095704	0.05365	0.028279
PE(20:4/22:2)	Glycerophospholipids	0.036104	-0.55366	-1.12848	-1.40203	0.461089	0.153713	0.001719	0.000171	0.476822	0.281606	0.031574	0.007048
PE(P-18:2/16:0)	Glycerophospholipids	-0.65967	-0.82465	-0.86965	-1.0651	0.048543	0.074287	0.005285	4.47E-05	0.135045	0.200655	0.05365	0.003119
PE(P-18:2/20:3)	Glycerophospholipids	-0.79807	-1.1557	-1.2334	-1.11779	0.001253	0.000122	3.73E-06	1.24E-05	0.020774	0.007128	0.000627	0.001443
PE(P-20:2/20:2)	Glycerophospholipids	-1.56728	-1.43213	-1.64054	-1.27486	7.03E-06	3.69E-05	5.37E-06	3.28E-05	0.001635	0.002859	0.000627	0.002546
PE(P-18:2/18:2)	Glycerophospholipids	-0.52204	-0.80443	-1.12382	-1.40226	0.052181	0.017829	0.000576	0.000172	0.136633	0.11711	0.019159	0.007048
PE(P-18:2/20:2)	Glycerophospholipids	-1.12596	-1.38181	-1.62322	-1.46111	0.000141	2E-05	6.23E-07	1.71E-06	0.008202	0.002327	0.000435	0.001194
PE(P-18:2/18:1)	Glycerophospholipids	-1.34364	-1.71902	-1.88436	-1.91779	0.00019	1.29E-05	6.29E-06	6.41E-06	0.008841	0.002251	0.000627	0.001443
PE(P-18:2/20:1)	Glycerophospholipids	-1.3963	-1.54926	-1.80592	-2.00009	0.080513	0.054312	0.107416	0.039186	0.015422	0.016427	0.008633	0.005671
PE(P-18:2/18:3)	Glycerophospholipids	-1.59694	-0.87394	-1.16568	-2.07055	0.034617	0.032079	0.072561	0.022068	0.093683	0.229632	0.144252	0.062994
PI(18:2/18:0)	Glycerophospholipids	1.24128	1.100703	0.976307	1.097714	0.010891	0.067275	0.053573	0.030691	0.071179	0.192596	0.162177	0.107649
PS(18:1/22:6)	Glycerophospholipids	1.929859	1.939216	1.816299	1.643889	0.000168	0.001636	0.008221	0.00035	0.008841	0.035426	0.065341	0.011098
TG(14:0/22:1/22:3)	Glycerolipids	2.530847	3.163348	3.558783	4.170396	1.71E-05	1.86E-05	1.97E-05	1.88E-05	0.077097	0.029454	0.081588	0.054107
TG(18:2/18:3/20:4)	Glycerolipids	1.907355	2.187941	2.712237	2.674074	0.029254	0.036686	0.064876	0.044103	0.012851	0.142277	0.050105	0.062994
TG(18:3/18:3/18:3)	Glycerolipids	-1.35806	-0.1598	0.454331	2.591791	0.030745	0.008457	0.010362	0.009289	0.071179	0.465283	0.354009	0.240122
TG(18:1/18:1/20:0)	Glycerolipids	-0.67098	0.062852	0.606658	2.179717	0.013116	0.00902	0.007992	0.00748	0.312787	0.478637	0.274283	0.170494
TG(16:0/18:0/22:3)	Glycerolipids	1.474583	1.821977	1.586085	2.106836	0.123512	0.469439	0.002025	0.001996	0.049522	0.05436	0.051679	0.005671
TG(14:0/22:3/22:4)	Glycerolipids	1.224878	1.453811	1.261318	1.878186	0.015747	0.011317	0.003613	0.006113	0.08153	0.096324	0.043978	0.046728
TG(14:0/20:2/22:2)	Glycerolipids	0.828815	1.400443	1.212017	1.86619	0.014444	0.005438	0.008918	0.003713	0.080657	0.073197	0.069162	0.03668
TG(18:1/18:3/20:0)	Glycerolipids	0.217134	0.744181	0.861473	1.664225	0.261735	0.033303	0.024577	0.01095	0.342118	0.140033	0.114488	0.062994
TG(14:0/22:4/22:4)	Glycerolipids	2.284213	2.586025	1.604119	1.476575	0.002087	0.047008	0.012708	0.034105	0.026971	0.165822	0.081588	0.113902
TG(18:2/18:2/20:0)	Glycerolipids	0.136132	0.657789	0.66104	1.35712	0.323551	0.03156	0.043491	0.01094	0.392081	0.140033	0.148081	0.062994
TG(16:1/20:1/20:2)	Glycerolipids	-0.12746	0.431547	0.363936	1.317693	0.328337	0.083451	0.153521	0.030472	0.393778	0.212478	0.272666	0.107649
TG(14:0/20:1/22:2)	Glycerolipids	-0.01997	0.435417	0.498753	1.179537	0.473213	0.087141	0.078368	0.025326	0.485026	0.218767	0.193046	0.09931
TG(14:0/20:5/22:4)	Glycerolipids	0.862815	1.448211	1.096723	1.171199	0.01581	0.052076	0.005474	0.026708	0.08153	0.175163	0.05365	0.103566
TG(14:0/20:5/22:3)	Glycerolipids	0.958314	1.25658	1.289513	1.164696	0.007246	0.022542	0.003835	0.00589	0.056262	0.129106	0.045365	0.046728
TG(14:0/18:3/22:1)	Glycerolipids	0.143305	0.530674	0.574017	1.14321	0.296111	0.071882	0.065143	0.039756	0.370786	0.198316	0.181154	0.126712
TG(14:1/14:1/22:3)	Glycerolipids	0.411214	0.858473	0.458976	1.110463	0.058527	0.078951	0.062302	0.043672	0.144279	0.205626	0.177496	0.130827
TG(14:0/20:1/20:1)	Glycerolipids	0.509187	0.821977	0.776724	1.10684	0.051154	0.004146	0.01739	0.008423	0.136281	0.062912	0.094828	0.054972
TG(14:0/20:2/22:4)	Glycerolipids	-0.01118	0.553877	0.307815	1.106744	0.488277	0.149325	0.185823	0.013816	0.491985	0.277943	0.295454	0.070392
TG(18:1/18:3/20:1)	Glycerolipids	-0.50991	0.373252	0.070203	1.096099	0.102354	0.162646	0.426741	0.021715	0.199773	0.291966	0.46226	0.091307
TG(14:0/20:3/22:1)	Glycerolipids	0.084783	0.4021	1.001136	1.078581	0.421262	0.126518	0.068758	0.003347	0.460158	0.253132	0.185316	0.035401
TG(18:1/18:3/20:4)	Glycerolipids	0.852752	1.108224	1.292052	1.077181	0.017804	0.017154	0.005894	0.009172	0.085116	0.11711	0.053874	0.057566
TG(18:0/18:1/18:2)	Glycerolipids	0.384664	0.433441	0.528006	1.040391	0.083429	0.008009	0.04538	0.0036	0.173313	0.081021	0.15282	0.036542
TG(16:0/20:4/22:5)	Glycerolipids	1.020815	0.976493	1.772852	1.035576	0.00601	0.017401	0.021907	0.048099	0.049522	0.11711	0.110178	0.138381
TG(14:0/20:3/20:5)	Glycerolipids	0.554584	1.000365	0.992563	1.035273	0.115594	0.081054	0.038692	0.041673	0.214662	0.208686	0.142895	0.126946
TG(18:0/18:3/20:2)	Glycerolipids	-0.07914	0.430927	0.267478	1.001518	0.411076	0.128127	0.222412	0.014944	0.452573	0.254774	0.323424	0.073169
TG(14:0/20:0/20:0)	Glycerolipids	-2.94121	-4.26743	-3.90332	-3.53402	0.021052	0.023798	0.026928	0.026418	0.170367	0.189304	0.180125	0.160488

Table S9. Lipidomic data of S vs H and M vs H

		Log_2 FC				*P* value				FDR			
Compounds	Class	ST1/H	ST2/H	MT1/H	MT2/H	ST1/H-P	ST2/H-P	MT1/H-P	MT2/H-P	ST1/H-FDR	ST2/H-FDR	MT1/H-FDR	MT2/H-FDR
Glycocholic Acid	Bile Acids	-1.562566307	-1.734053726	-1.902328797	-2.278651031	0.02306349	0.015077963	0.0122502	0.008120827	0.15938927	0.15098673	0.04621967	0.02391703
Cer(d18:1/18:1)	Sphingolipids	3.103373474	2.694176315	2.087149306	2.386808302	0.035759384	0.000440324	0.000512193	0.012090853	0.12561027	0.21183703	0.01461782	0.09740866
Cer(d18:0/18:0)	Sphingolipids	1.806648121	2.023396674	2.062599441	2.280526728	0.016516581	0.015809763	0.017251447	0.00675199	0.13251234	0.15116732	0.05817155	0.02138939
DG(16:0/20:2/0:0)	Glycerolipids	3.419566256	4.760460977	3.790298648	4.230188035	0.012879856	0.022808216	0.010695387	0.014146693	0.05161576	0.15668322	0.00210474	0.00098919
DG(14:1/22:3/0:0)	Glycerolipids	1.946986599	2.52513302	2.184108717	2.562694047	0.011953867	0.040682557	0.001654452	0.055821581	0.12460037	0.15568394	0.00179685	0.0014716
DG(16:0/16:1/0:0)	Glycerolipids	1.343366759	2.446915514	2.235581794	2.947646712	0.025046109	0.040006045	0.001941206	0.000638302	0.16619923	0.21183703	0.01566089	0.00441933
DG(16:1/18:3/0:0)	Glycerolipids	1.615029147	1.258379545	1.712432931	1.621531685	0.001638189	0.017733488	3.79113E-05	4.53497E-05	0.23014562	0.19501475	0.03205295	0.0029447
DG(16:1/20:2/0:0)	Glycerolipids	1.158272128	2.067739658	1.716402514	1.976972407	0.045185513	0.020005295	0.001901897	0.001195212	0.209088	0.17434653	0.01566089	0.00641737
FFA(22:0)	Fatty acids	-1.680426826	-1.715484992	-1.526469728	-1.970871915	0.017237641	0.01695126	0.020915218	0.011453033	0.13518959	0.15568394	0.06605802	0.03028113
LPC(12:0/0:0)	Glycerophospholipids	1.592738534	1.677657267	1.879575129	2.131799559	0.000213342	0.000792147	2.63225E-07	1.0296E-07	0.01861409	0.02764593	9.7622E-05	4.6645E-05
(±)12-HEPE [(±)-12-hydro:	Oxidized lipid	1.203063199	1.994467481	2.472382374	3.143034687	0.01085773	0.022254719	0.000927184	0.004009076	0.12561027	0.13828206	0.00414862	0.00021677
PC(18:0/20:3)	Glycerophospholipids	-1.938769454	-1.093608196	-1.223701153	-0.811688441	0.000176746	0.003882864	0.002374377	0.020975714	0.01831602	0.0774354	0.017558	0.04707733
PC(O-20:2/22:1)	Glycerophospholipids	-1.244412763	-2.774862923	-2.579115731	-1.341969722	0.029745143	0.027803613	0.02724922	0.027952109	0.40841061	0.28920277	0.2649314	0.31493431
PE(18:2/20:4)	Glycerophospholipids	1.194094437	0.937574948	1.057427307	0.734281086	0.002428119	0.008602052	0.001564624	0.030090036	0.06277137	0.12050477	0.01461782	0.06070456
PE(P-18:0/18:0)	Glycerophospholipids	-1.330736065	-0.612572106	-1.173211316	-0.352033952	7.82174E-06	0.015574562	1.12867E-05	0.024522692	0.00295969	0.15098673	0.0015093	0.05220853
PI(18:2/18:0)	Glycerophospholipids	1.166854807	1.717360487	1.600095702	1.643987518	0.000275676	0.000773877	0.006566721	0.00147547	0.01879801	0.02764593	0.03205295	0.00740919
PS(18:1/22:6)	Glycerophospholipids	1.91441964	1.740121496	1.830156627	1.866477265	0.001905388	0.000790976	5.03158E-06	6.39596E-06	0.05330562	0.02764593	0.00087801	0.00027902
TG(14:0/22:1/22:3)	Glycerolipids	2.45514207	3.332584857	2.912408929	3.142038268	1.99341E-05	2.08032E-05	1.94126E-05	2.25595E-05	0.12561027	0.18275052	0.01155669	0.01472808
TG(18:2/18:3/20:4)	Glycerolipids	1.956422308	2.626290364	2.971456773	2.741472617	0.012330324	0.010373693	0.000184251	3.41608E-06	0.05008103	0.0869969	0.00210474	0.00021677
TG(14:0/22:3/22:4)	Glycerolipids	1.110069554	1.001875522	1.249508459	1.388617688	0.027841387	0.065652631	0.001623118	0.008041958	0.1705897	0.22161326	0.01461782	0.02384662
TG(14:0/20:2/22:2)	Glycerolipids	1.104910867	1.287741589	1.210856405	1.593205222	0.028105753	0.024176167	0.001506904	0.002423129	0.1705897	0.18543917	0.0146086	0.0105709
TG(14:0/20:0/20:0)	Glycerolipids	-3.784389555	-0.839636612	-2.122050268	-0.297337049	0.02549273	0.032673181	0.028730109	0.035735915	0.23014562	0.35172628	0.18322975	0.4216428

Table S10. KEGG enrichment analysis of DEIs shared by F vs H, S vs H and M vs H

#KEGG n	16	KEGG N	524				
#Pathway	ko ID	Unique con	compound	Uni all	compound all		
Metabolic p	ko01100	14	418	16	524	LIPID-N-0	C01194+C00641+C02737+C00157+C00422+C00195+C00641+C00422+C00422+C00422+C00641+C00641+C00641+C00422
Adipocytok	ko04920	2	46	16	524	LIPID-N-0	C00162+C00195
Insulin resi	ko04931	7	237	16	524	LIPID-P-1	C00422+C00422+C00422+C00195+C00422+C00422+C00162
Leishmania	ko05140	2	17	16	524	LIPID-P-0	C00195+C02737
Glycerolipi	ko00561	11	287	16	524	LIPID-P-0	C00641+C00641+C00422+C00641+C00641+C00422+C00422+C00422+C00422+C00162+C00641
Inositol ph	ko00562	6	56	16	524	LIPID-P-0	C00641+C00641+C00641+C00641+C01194+C00641
Glyceropho	ko00564	9	217	16	524	LIPID-P-0	C00641+C00641+C01194+C00641+C00641+C02737+C00641+C00157+C04230
Phosphatid	ko04070	6	59	16	524	LIPID-P-0	C00641+C00641+C00641+C01194+C00641+C00641
Long-term	ko04730	5	55	16	524	LIPID-P-0	C00641+C00641+C00641+C00641+C00641
Thermogen	ko04714	6	227	16	524	LIPID-P-1	C00422+C00162+C00422+C00422+C00422+C00422
Regulation	ko04923	6	227	16	524	LIPID-P-1	C00422+C00422+C00422+C00162+C00422+C00422
Fat digestic	ko04975	6	234	16	524	LIPID-P-0	C00422+C00422+C00422+C00162+C00422+C00422
Vitamin dig	ko04977	6	231	16	524	LIPID-P-1	C00422+C00422+C00162+C00422+C00422+C00422
Cholesterol	ko04979	6	229	16	524	LIPID-P-0	C00422+C00422+C00422+C00422+C00422+C00162
Choline me	ko05231	3	123	16	524	LIPID-P-0	C04230+C00157+C00162

Table S11. KEGG enrichment analysis of DEIs unique to F vs H

#KEGG r	58	KEGG N	524				
#Pathway	ko ID	Unique co	compounc	Uni all	compound	all	
Glycerolir	ko00561	32	287	58	524	LIPID-P-(	C00422+C00422+C00422+C00641+C00422+C00681+C00422+C00422+C00422+C00641+C00162+C00162+C00422+C00422+C00641+C00422+C00422+C00641+C00422+C00422+C00641+C00422+C00422+C00641+C00422+C00162+C00422+C00422+C00422+C00422+C00641+C00422
Inositol pł	ko00562	7	56	58	524	LIPID-P-(	C00641+C00641+C00641+C00641+C00641+C00641+C00641
Glyceropł	ko00564	31	217	58	524	LIPID-P-(	C00350+C04438+C00350+C00641+C00641+C00641+C00350+C00641+C00350+C00157+C00350+C00350+C04438+C00350+C00350+C00350+C00681+C00350+C00350+C00641+C00157+C04438+C00157+C00350+C00350+C00350+C00350+C00641+C00157+C00350+C00641
Metabolic	ko01100	51	418	58	524	LIPID-P-(	C00350+C00422+C00422+C00422+C00422+C00422+C00641+C00422+C00639+C00350+C19615+C00350+C00350+C00422+C00422+C00350+C00422+C00157+C00641+C00422+C00350+C00350+C00350+C00350+C00641+C00350+C00422+C00350+C00641+C00422+C00641+C00422+C00641+C00350+C00422+C00350+C00157+C00422+C00422+C00350+C00350+C00350+C00681+C00422+C00422+C00422+C00157+C00422+C00422+C00157+C
Phosphati	ko04070	7	59	58	524	LIPID-P-(	C00641+C00641+C00641+C00641+C00641+C00641+C00641
Long-terr	ko04730	7	55	58	524	LIPID-P-(	C00641+C00641+C00641+C00641+C00641+C00641+C00641
Arachidon	ko00590	5	59	58	524	LIPID-N-	C00639+C00157+C00157+C00157+C00157
Phospholi	ko04072	2	7	58	524	LIPID-P-(	C00681+C00639
Neuroacti	ko04080	2	5	58	524	LIPID-P-(	C00681+C00639
Fatty acid	ko00062	3	34	58	524	LIPID-N-	C00162+C00162+C00162
Fatty acid	ko00071	3	35	58	524	LIPID-N-	C00162+C00162+C00162
AMPK si	ko04152	3	33	58	524	LIPID-N-	C00162+C00162+C00162
Thermoge	ko04714	24	227	58	524	LIPID-P-	C00422+C00422+C00422+C00422+C00422+C00422+C00422+C00422+C00422+C00162+C00422+C00422+C00422+C00422+C00422+C00422+C00422+C00422+C00162+C00422+C00422+C00422+C00422+C00162
Insulin sec	ko04911	3	33	58	524	LIPID-N-	C00162+C00162+C00162
Adipocyto	ko04920	3	46	58	524	LIPID-N-	C00162+C00162+C00162
Regulatior	ko04923	24	227	58	524	LIPID-N-	C00162+C00162+C00422+C00422+C00422+C00422+C00422+C00422+C00422+C00422+C00422+C00422+C00422+C00422+C00422+C00422+C00162+C00422+C00422+C00422+C00422+C00422+C00422
Type II di	ko04930	3	33	58	524	LIPID-N-	C00162+C00162+C00162
Insulin res	ko04931	24	237	58	524	LIPID-P-	C00422+C00422+C00422+C00422+C00422+C00422+C00422+C00422+C00162+C00422+C00422+C00422+C00422+C00422+C00422+C00422+C00422+C00422+C00422+C00422+C00422+C00422+C00162+C00162
Non-alcoł	ko04932	3	33	58	524	LIPID-N-	C00162+C00162+C00162
Fat digesti	ko04975	25	234	58	524	LIPID-P-	C00422+C00422+C00422+C00422+C00422+C00422+C00422+C00422+C00422+C00162+C00422+C00162+C00422+C00422+C00162+C00422+C00422+C00422+C00681+C00422+C00422+C00422+C00422+C00422+C00422
Vitamin di	ko04977	25	231	58	524	LIPID-P-	C00422+C00422+C00422+C00422+C00422+C00422+C00162+C00422+C00422+C00422+C00422+C00422+C00422+C00422+C00422+C00681+C00422+C00422+C00422+C00162+C00162+C00422+C00422+C00422+C00422
Cholestero	ko04979	24	229	58	524	LIPID-P-(	C00422+C00422+C00162+C00422+C00422+C00422+C00162+C00422+C00422+C00422+C00162+C00422+C00422+C00422+C00422+C00422+C00422+C00422+C00422+C00422+C00422+C00422+C00422+C00422
Choline m	ko05231	7	123	58	524	LIPID-N-	C00162+C00157+C00162+C00162+C00157+C00157+C00157
Linoleic a	ko00591	4	63	58	524	LIPID-P-(	C00157+C00157+C00157+C00157
alpha-Linc	ko00592	4	57	58	524	LIPID-P-(	C00157+C00157+C00157+C00157
Retrograd	ko04723	20	99	58	524	LIPID-N-	C00350+C00350+C00157+C00157+C00350+C00350+C00350+C00350+C00157+C00350+C00350+C00157+C00350+C00350+C00350+C00350+C00350+C00350+C00350+C00350
Glycosylp	ko00563	16	46	58	524	LIPID-N-	C00350+C00350+C00350+C00350+C00350+C00350+C00350+C00350+C00350+C00350+C00350+C00350+C00350+C00350+C00350+C00350
Autophag	ko04136	16	46	58	524	LIPID-N-	C00350+C00350+C00350+C00350+C00350+C00350+C00350+C00350+C00350+C00350+C00350+C00350+C00350+C00350+C00350+C00350
Autophag	ko04140	16	46	58	524	LIPID-N-	C00350+C00350+C00350+C00350+C00350+C00350+C00350+C00350+C00350+C00350+C00350+C00350+C00350+C00350+C00350+C00350
Pathogeni	ko05130	16	44	58	524	LIPID-N-	C00350+C00350+C00350+C00350+C00350+C00350+C00350+C00350+C00350+C00350+C00350+C00350+C00350+C00350+C00350+C00350
Kaposi sa	ko05167	16	44	58	524	LIPID-P-(	C00350+C00350+C00350+C00350+C00350+C00350+C00350+C00350+C00350+C00350+C00350+C00350+C00350+C00350+C00350+C00350

Table S12. Normalized expression levels of potential biomarkers

Compounds	Q1 (Da)	Q3 (Da)	RT (min)	Ionization mode	Method	Class	F1T4	F2T4	F3T4	F4T4	F5T4	F6T4	F7T4	F8T4	F9T4
L-Malic Acid	133.0	115.0	1.23	Negative	hydrophilic	Hydroxy acids and derivatives	47176	30332	26412	41652	26018	76817	44656	15549	14220
Carbamoyl phosphate	140.0	79.0	0.74	Negative	hydrophilic	Organic phosphoric acids and de	59483	9952	56363	16897	40263	10722	41912	152420	50902
D-Xylulose 5-phosphat	229.0	97.0	0.80	Negative	hydrophilic	Organooxygen compounds	171080	73049	206880	48742	199830	79214	100230	254050	455140
Guanosine Monophosp	364.0	156.7	1.10	Positive	hydrophilic	Purine nucleotides	51051	4954.7	16586	6964.3	6729	6868.6	5359.8	4826	9827.8
Dihydrouracil	115.0	56.0	1.10	Positive	hydrophilic	Diazines	16370	13595	40482	13987	13085	55976	25089	60296	15101
FFA(22:0)	339.3	339.3	6.32	negative	hydrophobic	fatty acids	231930	72724	64112	224330	115360	183530	67835	264130	252650
CE(20:1)	696.7	369.4	14.80	positive	hydrophobic	Steroids and steroid derivatives	2665.5	1529.8	4146.1	1030.2	3175.7	288.76	457.99	457.56	5703.6
LPC(O-20:1/0:0)	536.4	184.0	3.17	positive	hydrophobic	Glycerophospholipids	66253	55294	98328	48869	86851	173070	99356	104900	154160
PC(18:0/20:3)	812.6	524.6	7.31	positive	hydrophobic	Glycerophospholipids	25464	15915	43066	13901	64679	1469.7	72155	2130.1	70690
PE(P-20:2/20:2)	780.6	639.6	7.53	positive	hydrophobic	Glycerophospholipids	56996	26492	100890	160390	49123	95423	109900	104430	65404
Glycerol 3-phosphate	171.0	79.0	0.90	Negative	hydrophilic	Lipids Fatty Acids	171900	115590	208950	90439	148960	191470	306170	125690	727790
Hyodeoxycholic Acid	391.3	391.0	6.80	Negative	hydrophilic	Bile Acids	11259	9.6893	8.1249	11.655	10.078	39858	4417.6	7813.9	576.95
O-Phosphorylethanola	140.0	79.0	0.74	Negative	hydrophilic	Glycerophospholipids	44638	2833.1	21814	12861	28299	44501	35211	46579	22247
LPE(0:0/20:2)	506.3	365.3	2.86	positive	hydrophobic	Glycerophospholipids	14951	3165.2	3900	5320.8	12396	10268	10012	9067	30871
DG(16:0/20:2/0:0)	638.6	365.5	9.18	positive	hydrophobic	Glycerolipids	13831	4797.9	3350.6	19104	14702	5377.8	12834	6520	17112
DG(14:1/22:3/0:0)	634.6	391.5	6.99	positive	hydrophobic	Glycerolipids	4448.3	5901.1	2429.9	25876	4595.4	5553.6	10322	1822.2	9922
LPC(12:0/0:0)	440.3	184.0	0.79	positive	hydrophobic	Glycerophospholipids	533930	524510	676430	398410	576970	832630	898030	787060	953480
PS(18:1/22:6)	834.5	649.5	5.56	positive	hydrophobic	Glycerophospholipids	15191	15648	10171	6340.1	7973.6	10260	17998	22123	13589
TG(14:0/22:1/22:3)	956.9	601.8	12.98	positive	hydrophobic	Glycerolipids	14734	5232.4	369.02	2257.8	2258	6788.5	4805.4	190.24	6310.9
TG(18:2/18:3/20:4)	918.8	623.7	10.94	positive	hydrophobic	Glycerolipids	24164	9420.8	17332	143380	93903	55883	18734	47921	32765

Compounds	S1T1	S2T1	S3T1	S4T1	S5T1	S6T1	S7T1	S8T1	S9T1	S10T1	S11T1	M1T1	M2T1	M3T1	M4T1	M5T1	M6T1	M7T1
L-Malic Acid	53091	46098	51020	22305	25629	25500	25690	28114	17275	25686	194130	49712	15970	23101	52440	20737	38379	40052
Carbamoyl phosphate	55099	56038	113290	39204	81407	19372	56351	150440	147670	141750	16368	28265	49481	60299	133510	80155	102990	123170
D-Xylulose 5-phosphat	152090	212720	199680	185700	261010	132690	261910	207660	352330	324540	226330	135260	166960	224820	221750	279580	256750	236850
Guanosine Monophosp	11746	17387	30354	6313.8	23073	8821.1	6596.5	25740	6767.4	16594	12378	4851.8	10302	8525.1	26282	16077	25167	40546
Dihydrouracil	20254	26867	34815	18769	30813	11779	21987	9393.9	53484	61933	22399	28129	8087	5101.4	31517	36729	40488	34815
FFA(22:0)	87084	106140	79672	73800	84810	74028	74832	71497	295170	526820	434960	77366	57674	69831	82116	112480	89856	74417
CE(20:1)	2232.4	1972.6	1426.8	2444.1	4442	1591.2	2066.1	3833.3	273.82	328.41	335.91	1266.8	2094.7	676.62	1786.6	4485.8	2730.4	1518.6
LPC(O-20:1/0:0)	40864	106770	77475	50666	123370	17146	124790	107280	147680	129820	91985	118170	74701	122960	131590	137950	89784	78947
PC(18:0/20:3)	12874	23946	24659	32582	30773	16966	88624	38765	12945	7467.2	3682.3	60343	26236	24804	81991	39560	43510	25218
PE(P-20:2/20:2)	31175	67571	59775	113630	142940	9212.3	251540	121970	54697	68930	57631	97846	30996	99490	107200	196040	133310	53892
Glycerol 3-phosphate	98015	193520	160640	204280	186740	169060	204940	110580	637320	417070	193240	80553	148700	157170	133310	150370	191740	150140
Hyodeoxycholic Acid	11.008	10.381	10.286	9.4448	9.6626	11932	5244.2	8.7901	17931	72258	3931.6	13.936	8.8006	7.8141	30224	32729	7.8397	70758
O-Phosphorylethanola	39627	23683	79875	17663	81584	17957	43212	200380	91247	100780	51079	37704	48929	47579	118350	64932	114150	130300
LPE(0:0/20:2)	2246.1	10962	2463.9	6375.8	13412	986.74	8351.8	15014	21301	21606	14324	10368	5225.4	17303	18658	9839.6	6026.1	7152.2
DG(16:0/20:2/0:0)	457.82	3656.8	2226.5	16336	3425.4	705.99	8292	6554.2	7452.8	9293.4	13312	4887.4	2531.5	3853.6	18549	4936.8	10402	3130.8
DG(14:1/22:3/0:0)	282.99	2963.3	3765.2	6035.3	3068.7	726.58	3032.9	6907	2485.4	4901	13908	5208.1	1690.9	3907.7	10497	2987.7	5331.8	2879.7
LPC(12:0/0:0)	328760	552470	298900	525450	557450	228730	463020	628140	877880	951020	943330	526140	589730	592630	479730	636430	675860	401840
PS(18:1/22:6)	4847.7	16964	4057.1	13884	17005	5121.8	13518	21818	34485	11883	32292	15251	8878	12814	8743.3	13098	13359	10757
TG(14:0/22:1/22:3)	214.9	801.77	107.5	4090.8	1919.4	413.63	718.9	1776.5	224.94	1736	3981.4	817.43	276.38	3416.9	1807	1248.5	1159.6	695.68
TG(18:2/18:3/20:4)	12241	37259	14758	30514	54908	3257.8	20721	21305	64519	24459	45677	36998	14054	35062	125300	30975	33051	36635

Compounds	M8T1	M9T1	M10T1	M11T1	M12T1	M13T1	M14T1	H1	H2	H3	H4	H5	H6	H7	H8	H9	H10
L-Malic Acid	46765	41558	132180	41861	48789	270300	18894	1036800	1058500	283650	4261400	2153400	435280	381770	510830	187480	2684000
Carbamoyl phosphate	92955	93483	110180	185810	145270	72272	98112	1253700	1004600	296350	939920	816400	557790	637860	85128	344130	258050
D-Xylulose 5-phosphat	292070	215820	115170	308280	393380	209830	193410	511860	666320	693750	848930	815030	638000	531890	367610	498610	655490
Guanosine Monophosp	13733	35162	48338	40539	7669.3	37132	41968	27895	75990	45363	43012	32968	23892	21975	69866	46231	24129
Dihydrouracil	43746	27643	44913	18.624	65827	44179	47542	50260	106380	168130	200290	92012	155010	108580	108750	63013	20094
FFA(22:0)	113070	106910	263590	510740	355990	253140	535810	333890	155150	251740	410430	604060	782860	1445600	1279300	62322	236630
CE(20:1)	2160.1	2765.4	585.43	381.02	396.25	304.59	445.47	16078	20145	3444.3	19263	23204	22188	641.91	341.91	468.01	272.3
LPC(O-20:1/0:0)	143310	162370	333230	227500	178310	152740	275360	263210	294540	229830	116920	87362	164160	202240	267640	132730	219900
PC(18:0/20:3)	61672	69088	2840.3	8406.4	3098.2	3458.4	162520	123600	108400	82136	3004	84263	133210	89416	175800	96430	125910
PE(P-20:2/20:2)	117110	118040	127150	118900	163630	95682	110620	141110	250700	147800	158240	244080	238040	276320	140750	185330	285300
Glycerol 3-phosphate	215210	144500	104590	515290	99096	444470	429710	4569700	5710700	3843200	5761000	5291100	4621000	2019600	2827000	4054800	946440
Hyodeoxycholic Acid	6471.1	8040.6	11228	15703	8726.4	8163.1	5029.1	26278	11153	176360	43926	18658	42218	48699	118690	20941	208320
O-Phosphorylethanola	65735	127020	37617	70326	67561	70432	58592	1061700	1184200	421050	387180	464850	222260	197980	59752	71590	53852
LPE(0:0/20:2)	8755.9	14813	45670	16437	12434	17902	23091	796.65	462.55	929.53	423.49	297.54	370.96	134.68	1026.8	1718.9	884.86
DG(16:0/20:2/0:0)	7039.1	9357.1	5766.3	16583	13010	4910.6	13057	582.67	918.01	933.59	191.97	608.71	802.86	580.31	506.05	358.62	609.95
DG(14:1/22:3/0:0)	7595.9	9104.2	6157.7	5920.5	3848.4	4876.5	2112.6	235.31	276.34	564.43	794.55	3960.3	1860	2114.5	488.54	261.42	780.03
LPC(12:0/0:0)	487780	626580	1068400	1010300	1020500	743390	1008200	194820	170180	265860	217720	172050	87845	307780	71856	149020	278320
PS(18:1/22:6)	11453	14852	26910	15131	19429	21062	19405	7838.5	1183.6	1955	1875.8	3167.6	1505.5	4838.4	597.68	14234	5218.5
TG(14:0/22:1/22:3)	875.73	563.48	358.8	4096.5	4409.4	3309.3	4898.4	266.74	203.59	79.879	145.2	63.609	123.88	80.145	1132.8	69.136	485.16
TG(18:2/18:3/20:4)	100670	98695	49596	117940	63639	44740	60464	17263	2378.6	2992.1	3842.7	5575.7	2001	6228.3	23197	8204.7	5527.6

Table S13. Clinical characteristics of COVID-19 patients in this study

	Fatal (n=9)				Severe (n=11)		Mild (n=14)	
	F1	F2	F3	F4	S1	S2	M1	M2
**Characteristic**								
ALT (normal range 9-50 U/L)	46(22-66)	31(22-49)	39(27-59)	40(29-109)	39(24-56.5)	47(32.5-78.5)	29(13.8-37.5)	33(25.5-65)
AST (normal range 15-40 U/L)	38(34-74)	44(25-53)	32(27-42)	56(41-228)	29(24-43)	33(21.5-36.5)	26.5(20.3-30.3)	22.5(14.3-27.8)
Total bilirubin (normal range 0-21 µmol/L)	15(12-21.2)	25.6(12.2-35.1)	17.5(16.1-23.5)	18.3(16.2-19.7)	10.5(10-13.6)	10.1(7.55-13.1)	13.3(11-16.8)	9.3(7.4-11.4)
**Patients with pre-existing liver conditions**	1 (11.1%)				0 (0%)		2 (14.3%)	

Data are median (IQR)

Table S14. Metabolite identification methods and datasets

Compounds	Identification method	H1	H10	S1T1	[illegible]	[illegible]	[illegible]	[illegible]	[illegible]	[illegible]	[illegible]	[illegible]	[illegible]	[illegible]	[illegible]	[illegible]	[illegible]	[illegible]	[illegible]
Allantoin	Reference standar	40423000	13312000	468180	298090	424980	1500500	1509600	587140	405930	2026500	423420	529050	541230	73455	405820	511610	445900	391640
Pyrroloquinoline Quinone	Reference standar	494400	707470	885570	342920	1326300	842520	1620900	474310	647960	253880	620140	793930	373750	216610	1172300	371310	295780	576940
Citric Acid	Reference standar	2344600	5562600	1980200	2789700	2730800	4122000	11490000	1982800	3807100	1595100	1934400	3958800	1551000	1783400	2105300	4015300	626540	9015300
L-Malic Acid	Reference standar	1036800	2684000	34823	17083	48965	34592	45767	25083	47176	14220	53091	194130	24078	203760	49712	18894	45942	74533
Succinic Acid	Reference standar	190300	272420	43094	231070	45051	436140	383610	359340	68453	521840	33169	78875	41785	78040	35824	69151	63076	71099
Cis-Aconitic Acid	Reference standar	3981.4	5352.9	3092.1	2053	1976.4	2120	910.29	4733.3	1095	5439.3	4182.7	12273	4642.7	2223.1	2220.2	2818.8	10534	11065
Pyruvic Acid	Reference standar	3810.3	32771	5728.5	2879.2	13483	8226.2	6599.3	2831.4	10382	2224.8	11673	6886.5	11620	21624	10909	20859	7852.4	31440
Citramalic Acid	Pulic databases	1332900	141380	63100	76716	65091	178970	59966	75135	89479	71218	13566	58907	25109	30135	23188	35164	53971	26421
DL-o-Tyrosine	Pulic databases	1145500	922060	1651300	1095200	730840	1771300	1530800	1197600	2758100	1248600	1322800	1088300	1217500	1471200	1261000	1757800	901180	1930200
D-(+)-Malic acid	Pulic databases	483070	486720	449050	214210	484920	390480	1029000	281200	881050	359670	300950	242530	286800	299830	255190	312210	222450	337620
O-Acetyl-L-serine	Pulic databases	15501000	3941300	277050	1884300	274060	1758100	253550	54070	225710	2264900	331790	4024.7	261360	585090	220980	387550	187190	357740
Formononetin	Pulic databases	4425900	4429600	1122200	4011500	877620	2870200	1079100	4342500	5385600	3895000	4309100	3329900	3982500	1442600	8311900	7886800	9926900	6231700
Ellagic acid	Pulic databases	9487.6	4747.5	1847.2	53271	3.7179	5583.2	6029.7	21706	11331	7294.2	2140.2	2013.1	2314.7	6608.3	2565.5	885.86	3866.9	1484.9
N-Oleoyl Glycine	Pulic databases	4286.3	2725.4	2784.4	2982.6	2670.5	3553.9	2919.7	3671.3	2917.2	3949.1	2717	4466.7	2465.6	2564.1	3510.8	3927.5	3218.3	2683.9
Gamma-Glu-Leu	Pulic databases	402530	180290	497450	483330	170440	1128300	610630	92738	616600	304170	286610	378340	228100	404270	311550	154230	261660	437530
octadecanedioate (C18)	Reference standar	18617	67446	17191	80580	15501	16439	24553	526020	13892	5995.3	12871	14404	19342	12837	23469	657.97	17040	26104
N-Acetylhistamine	Pulic databases	157260	31620	29833	63155	123900	64158	167280	86312	105550	122680	168970	41409	50240	155250	74829	72573	94422	51771
Terpinolene	Reference standar	643630	15971	96259	30270	110790	23984	105670	27061	98219	94710	94878	171910	84712	162410	95012	93865	91755	72355
gamma-Terpinene	Reference standar	1208300	3379900	2108600	1982500	2010700	1049300	2253100	2808800	2343300	2235200	2487400	1020900	1384400	918250	1751800	616350	2281500	604620
alpha-Terpinene	Reference standar	29033000	35430	401340	312540	395730	129690	397680	80551	387700	103760	374660	194580	356220	231800	370710	728450	384040	848880
(+)-borneol	Reference standar	158860	170610	115000	71302	191320	145330	156620	139940	269470	139920	155090	125410	130300	169590	209050	227270	136170	157630
p-Menth-1-en-4-ol	Reference standar	197050	200610	140230	138220	278230	220620	180040	149130	335020	241620	240490	226010	175930	105570	233820	318700	159270	268020
Norambreinolide	Reference standar	1177.7	763.77	891.26	1455	1717.8	836.11	1099.8	1481.2	625.6	697.06	953.77	2216.8	795.57	1020.2	884.73	3400.4	1024.5	2625.5
2-Undecanone	Reference standar	375390	274990	342880	359780	319220	369940	327070	372310	357100	427330	346160	322420	393810	315340	302260	380090	325710	344820
Undecanal	Reference standar	212120	217040	226090	252000	243060	271790	208700	263720	223050	274520	253160	212120	284470	221140	253080	217550	221090	226500
Pyrene	Reference standar	52125	52582	53652	47205	48855	45070	58647	43510	51344	43992	38250	49020	47569	60799	52738	46799	54461	43630
Bisphenol propane	Reference standar	6488100	5523500	7791600	4611000	5518000	3701800	9868600	5378500	12764000	5250000	4074800	6344600	5242600	6294700	5271800	6006200	4613900	6511700
1,3-diisopropylbenzene	Reference standar	241110	242850	256050	235040	282150	238260	260990	244100	246180	256840	246370	261230	236820	279040	256350	263010	228090	274470
p-Cymene	Reference standar	204460000	79818000	6611900	23457000	7164700	4897400	4816900	11040000	4293900	11170000	7026500	3075300	5089700	3110000	3861500	2842700	5034400	14719000
Glycyrrhetinic acid	Reference standar	103490	168890	106600	4639.2	105860	385.66	63666	1204.3	17667	1097.6	130200	57499	165680	1041.8	170840	1891.5	181580	18305
L-Cystine	Reference standar	67299	22285	12100	36155	1673.2	37106	6963.6	3344.6	2938.9	17129	6911.2	913.22	8949	668.96	7918.4	1268.4	17649	8813.7
L-Threonine	Reference standar	361530	566650	701820	421640	359910	678950	556010	660070	1015700	678540	317940	470590	303780	218270	616250	366800	570020	745060
L-Arginine	Reference standar	443330	542830	691660	279390	208880	387140	502410	637950	467570	513410	186650	148080	411310	525130	581150	768260	618740	323620
L-Aspartic Acid	Reference standar	3188100	1914800	129350	511290	194280	450310	251590	445450	105620	585530	132410	68790	151830	56036	149230	53952	107670	38957
L-Citrulline	Reference standar	889830	1482900	1057500	848860	455090	1144100	1206100	1309500	1184100	1144400	919530	623400	1018900	668670	1638300	1910800	1849800	1822600
L-Glutamic Acid	Reference standar	4412000	1123100	4694200	3031900	1610200	3132700	1598300	6126800	1738700	5295000	1640200	1728900	1336700	2227800	1816200	1951300	3450400	1853800
L-Isoleucine	Reference standar	215750	164750	162100	166510	104460	242650	339230	137240	267600	232720	181760	165000	138830	249700	180450	138040	149240	201690
L-Pyroglutamic Acid	Reference standar	135100	85715	141580	115420	127660	240510	153080	126050	241450	105960	158860	132270	131500	113770	104160	123600	147950	156940
L-Serine	Reference standar	128360	197390	217480	150630	120950	213590	108180	143810	213470	134380	98346	219590	95894	123880	181040	104340	222620	174500
Dopamine	Reference standar	162070	57303	38903	26089	38174	35498	31338	40288	36682	36617	20828	30007	29175	16611	25443	20107	32925	14564
Hexanoyl Glycine	Reference standar	10285	15403	8217.8	13857	6722.1	13881	16480	13127	21991	15210	9318.3	10044	9585.4	12562	15013	3088	9317.1	4822.9
L-Asparagine Anhydrous	Reference standar	95702	121190	141670	87021	67217	139300	156790	189780	248760	149910	69720	77912	95513	84798	123560	75738	128930	104750
L-Carnosine	Reference standar	19469	14919	24326	15718	25016	28398	45352	14212	15777	15894	17995	27841	6301.9	15901	19333	18156	6878.7	19599
L-Cysteine	Reference standar	625110	843680	267820	322420	235540	546870	259260	358580	255610	584450	261340	962540	233870	561110	268230	367230	310850	603390
L-Glutamine	Reference standar	1115600	1608900	1264900	338250	538980	745720	2022000	108690	1921000	283790	668210	711980	1001400	430470	1552300	1065600	1275400	925560
L-Homocitrulline	Reference standar	31042	59283	32126	45790	9322.5	99133	41843	110960	25290	72380	39427	13963	55447	11640	44171	9382.9	36795	27818
N-Acetyl-L-Glutamic Acid	Reference standar	106160	64223	65152	79301	87121	139040	100840	73396	155330	102910	83650	103570	74415	87124	60807	90275	92309	85165
N-Acetyl-L-Leucine	Reference standar	168800	125360	19020	97900	28013	30070	92982	14251	50057	14406	30628	28043	15987	6924.9	21095	24592	21108	5930.6
N-Acetylneuraminic Acid	Reference standar	31588	5504.7	2366.5	5931.1	3139.3	2240.5	2300.6	3126.5	2107.7	5716.1	2395	2724.7	3445.8	1585.2	2376.1	2469.6	2055.8	1745.4
N-Isovaleroylglycine	Reference standar	66109	4245.3	4081.5	2912.3	2200.9	10675	11242	44604	16637	10685	17004	862	6071.5	2461.5	12657	3219.5	3061.2	2858.3
N-Propionylglycine	Reference standar	24449	9843	6554.5	17392	7949.1	7277.5	7988.9	17881	7313.4	11426	32346	4693.9	12706	33090	19900	3546.3	9342.3	9458.8
O-Phospho-L-Serine	Reference standar	56105	25884	77137	81676	16986	38840	39497	50998	40646	90957	29764	30090	51029	46866	59993	34627	101140	58284
S-Sulfo-L-Cysteine	Reference standar	317520	416580	186460	166450	133380	254800	69721	185900	136300	115970	226660	56165	54586	23980	85717	21130	54453	39131
Trans-4-Hydroxy-L-Proline	Reference standar	513880	361170	496050	404820	277910	675090	773950	382610	681810	561060	408440	438920	281690	650600	380380	333810	361520	500480
Benzoic Acid	Reference standar	154360	1268100	244680	232090	742360	174890	1792200	176660	213020	137170	365930	109420	286530	119960	193700	145270	128800	148000
Terephthalic Acid	Reference standar	1011800	1341700	1191600	1230300	1204700	1106900	1114500	1090600	842850	1304800	1124000	1379600	1188400	1155900	1232500	1155600	1383300	969590
(3,4-Dimethoxyphenyl) Acetic	Reference standar	54863	28020	65844	47003	57530	58769	46680	25352	63856	75216	30735	29587	14917	55575	14527	14238	15652	23947
2-Methoxybenzoic Acid	Reference standar	3981.9	1296.8	0.70591	1545.7	303.14	1620.3	380.55	1238.7	7661.8	123.75	356.03	6664.8	0.57648	12095	363.73	573.27	0.5809	785.37
4-Hydroxybenzeneacetic A	Reference standar	471570	334270	492920	429500	180540	397870	451350	545980	333420	624670	325830	347790	411120	408070	392490	404640	518600	580890
3-Hydroxyanthranilic Acid	Reference standar	7873.3	2151.6	14747	13070	5.824	41204	22381	71065	32354	18382	10357	3922.3	8676.9	8823.7	12656	6262.2	21574	6123.1
P-Hydroxyphenyl Acetic Acid	Reference standar	43057	37786	91195	57933	20222	191130	141060	333670	218900	80214	76753	35666	46112	78747	83462	36236	101020	60406
Phenethylamine	Reference standar	123620	1018700	135460	144640	524160	155450	1327500	136880	94947	110950	343250	103320	281910	95779	95478	109210	94839	115830
Dulcitol	Reference standar	457240	555420	374670	573250	233.36	568120	264390	691370	826400	615350	135.94	468860	111090	591130	100870	557750	170690	438840
1-Methylxanthine	Reference standar	303.98	19734	5065.1	9312.4	9883.4	12149	18806	8673.7	5110.9	9702.4	9254.8	6822.7	7333.1	8966.9	6856.7	7450.2	5951.5	5828.4
2,6-Dihydroxypurine	Reference standar	520050	339460	222670	381890	583670	984440	661270	695090	1019300	969240	1162100	615190	586730	683120	219250	320690	452390	559290
5-Methylcytosine	Reference standar	8158.5	6062.5	7333.5	7942.7	2338	14903	13923	13081	3189.4	9213.8	7267.9	9246.6	5546.9	10820	3107.6	8354.1	4089.5	11180
Adenosine 5'-Monophosphate	Reference standar	246270	33847	38224	14314	36936	10896	36524	39741	119310	21338	89841	11085	104210	40008	19523	40888	92224	19057
Hypoxanthine	Reference standar	377340	142550	6459.7	183090	501750	234560	555510	221340	1608400	244840	602530	56918	465540	330640	210430	127640	198640	249600
Thymidine	Reference standar	31570	11131	27861	22601	10994	14588	7543.1	14498	5400.8	14460	25713	6099.6	15591	2503.7	20659	30754	26686	37236
B-Nicotinamide Mononucleoti	Reference standar	10235	8294.1	4574	8937.3	6830.4	10303	9091.4	9436.5	5293.3	10936	13061	8976.9	9375.6	9727.9	14849	9437.6	12705	8010.4
Epinephrine	Reference standar	27405000	85185	194010	168620	219880	198750	190730	186740	214090	187800	174770	167080	181340	172310	177470	185630	184810	189780
L-Thyroxine	Reference standar	14356	17023	13049	15465	13096	15727	10706	11196	5484.8	6292.5	14005	13881	17859	12106	11602	15876	13930	16656
Tryptamine	Reference standar	212690	218570	173310	207350	178820	207600	125150	222090	183050	283910	209430	208590	211640	326850	189200	298570	255910	308330
1,5-Anhydro-D-Glucitol	Reference standar	17377	21278	3299.3	9050.8	1.7252	6768.2	1.7156	816.61	5403.4	338.17	1.4891	7136.6	1.5376	10163	2.0588	2238.8	1.3678	1189.7
D-Trehalose	Reference standar	128260	102530	86750	162130	118060	105420	59876	118880	130740	142890	116290	105960	111550	139920	99744	125750	127150	108610
D-Erythronolactone	Reference standar	1684400	1477500	1440100	1571600	1531900	1563700	1311800	1638500	1374100	1782400	1476400	1553600	1649200	1655700	1541300	1637900	1556300	1560100
D-Arabinose	Reference standar	55636	73569	45171	117630	47835	105130	225350	152110	352040	85400	116680	64949	181620	28105	72423	57913	97948	80676
L-Fucose	Reference standar	337650	468370	24703	231170	525.57	155790	4741.2	26730	86763	59826	1560.3	41401	7731.1	39840	3928.9	628.85	597.26	3543.4
L-Rhamnose	Reference standar	216740	313470	18959	218220	3950.1	163840	8997.4	30119	77471	77344	7103.9	38930	4177	37120	6084.7	2311.1	6227	1113.6
D-Glucoronic Acid	Reference standar	910300	51605	100700	55148	156880	113850	168850	104390	271500	81560	52229	54302	65852	89652	52471	42550	61819	66929
D-Glyceric Acid	Reference standar	31996	7254.8	36596	34123	26955	61975	26320	12949	46191	46032	17372	18587	11614	46588	15200	8431.7	10265	21552
L-Gulonic-Γ-Lactone	Reference standar	1381200	116510	167710	113010	173430	107070	172660	116390	156540	109160	151210	179270	152290	169840	177780	152380	150480	185950
Orotic Acid	Reference standar	79586	268700	48609	54901	49588	112880	70197	74246	43543	63683	69178	35928	52628	43161	69337	51057	50242	30361
Pantothenate	Pulic databases	283720	220130	201860	297590	107650	441940	209000	373810	157380	209530	271300	269330	296850	283320	300640	276510	356310	408200
3-Indolebutyric Acid	Reference standar	22522	19832	21845	24236	21191	34330	17691	21336	30035	28985	19218	16710	15329	19674	16070	19287	23306	17010
Indole-3-Carboxaldehyde	Reference standar	15141	17895	3655.6	16520	10925	16631	21120	24502	19447	16701	16289	17980	9489.5	26752	17863	31437	27153	58282
2,6-Diaminopimelic Acid	Reference standar	256950000	74769	624400	624840	547340	323060	657360	553920	658060	506920	508260	738170	401100	845790	610140	911890	571730	726950
2-Aminoethanesulfinic Acid	Reference standar	134190	2458.5	25023	5783.3	24601	16145	35917	20218	12317	12762	27940	9675.8	14580	35759	6664.3	15724	20255	13998
2-Aminoethanesulfonic Acid	Reference standar	3481200	2657100	2080600	2240200	1545200	4177000	1931000	2724000	1102900	1557100	2291200	1529400	4561100	2914600	2067500	5213600	7045600	4783200
2-Hydroxybutanoic Acid	Reference standar	16317000	8963800	24020000	14121000	16921000	21608000	19667000	7644900	25637000	22809000	8311400	9934700	4771600	23000000	5001700	4808200	4866600	7562200
2-Hydroxyisocaproic Acid	Reference standar	2867300	59787	75709	25083	78993	88673	148420	109560	127220	90851	44865	36662	37528	100300	49707	31597	41746	37536
3-Hydroxy-3-Methyl Butyric A	Reference standar	1759500	856300	1246000	2475300	888020	4569700	2016800	2606400	2173300	2660400	1879000	652580	1041100	1611000	930240	634450	457850	660630
3-Hydroxybutyrate	Reference standar	2949000	5730000	2314600	9971200	322880	7470300	576110	799130	8527500	2541700	377610	5113000	296640	5760300	283180	232710	236160	423570
3-Methylcrotonyl Glycine	Reference standar	4249	1390.7	0.55726	6643.3	0.51442	4780.2	1863.1	7004.8	5426	1655.4	2614	3413.5	686.85	1817	0.64033	6001.6	0.42907	31643
4-Hydroxy-2-Oxoglutaric Acid	Reference standar	233090	158610	36560	18687	49145	37641	15245	38474	50818	40487	34658	79531	15918	113320	19446	52287	17330	124030
Adipic Acid	Reference standar	21680	31737	39235	85286	15335	193660	81135	48408	132410	102750	25231	34661	28664	21430	12747	13215	15383	8478.1
Creatine	Reference standar	236480	229840	176490	45353	92519	25614	646870	52646	719520	79474	120600	43315	57402	371030	59994	195780	76825	192370
Dodecanedioic Acid	Reference standar	6099.1	7301.2	6243.2	7668.5	3088	7039.4	5785.2	7582	5683.7	6817.4	9567	4531.6	5439.9	6517	6751.2	5375.8	6428	5639.7
Kinic Acid	Reference standar	681690	849140	500570	789500	538890	759790	2228200	502190	794060	414170	340480	904910	207680	576610	426540	1201600	195080	1610200
L-3-Phenyllactic Acid	Reference standar	114820	74028	192280	87022	183900	161770	414300	219860	265530	114900	200120	178530	60570	141200	74751	61130	52031	71989
L-Lactic Acid	Reference standar	1170000	445130	666890	535710	1358800	729900	686270	882890	596720	674840	1113400	426950	753710	605240	500500	539290	700110	875870
L-Dihydroorotic Acid	Reference standar	95277	96025	114830	63919	134750	79609	134880	88214	112090	75950	90259	105960	78331	66755	83798	83235	84418	89031
Malonicacid	Reference standar	3003700	6096800	2611100	10950000	346570	8125500	606640	926390	9719400	2787300	421870	5741900	327930	6465900	303420	286510	236140	438540
Mandelic Acid	Reference standar	65618	62041	155930	99036	30570	323770	228960	539240	357400	134620	133220	53157	79293	133220	140460	66394	161930	103570
Methylmalonic Acid	Reference standar	1344700	848930	657300	567800	646770	1089200	5307000	1370500	894510	1737700	434610	596720	381820	558800	423780	488510	816960	793470
N-(-Acetyl-N-2-Formyl-5-M	Reference standar	148.79	246.8	0.36597	95.599	152.89	92.958	0.3393	350.93	1557.8	174.59	282.95	127.71	0.31346	127.22	0.42826	146.05	0.28493	134.38
Phenyllactate (Pla)	Reference standar	101300	63280	114920	67142	131110	107100	276720	153040	177250	85127	126380	111160	38607	98725	55955	43654	34568	53911
Phenylpyruvic Acid	Reference standar	8500800	40907	83090	117140	144370	185850	140150	124510	8.6112	106420	140440	67112	92916	47562	70405	66936	61736	57686
Pyrrole-2-Carboxylic Acid	Reference standar	4455.4	3910.3	7923.5	18979	6855.1	17787	3742.7	8413	8587.1	19505	18235	7046.7	9380.1	10738	8.2638	7918.9	6122.4	10917
Shikimic Acid	Reference standar	459580	1460500	9395.9	33778	105090	25966	177290	28301	413310	6093.3	122960	14541	50916	171520	65136	4653.3	427900	17667
Subericacid	Reference standar	133240	123950	143800	145810	135760	200880	130920	134610	178150	143090	129270	131920	132720	137710	137090	118090	137840	125680
Neopterin	Reference standar	1817900	1045500	123650	374020	114370	202720	112440	15035	115970	280080	130050	14805	141130	140950	136380	31903	134480	123610
4-(aminomethyl)-5-(hydroxy	Reference standar	21247	12537	9309.2	10029	7596.2	17117	12596	22912	1701.8	26808	14536	10189	15877	13467	16589	16648	21722	20279
Ureidoisobutyric Acid	Reference standar	35143	10872	70908	13081	26415	34645	13782	33471	8649.2	23156	25544	21895	18644	21279	5940.4	16678	9947.6	15471
N-Acetylglycine	Reference standar	64137	98990	43602	67100	18423	89872	30292	52364	56766	89301	43611	64535	49050	124960	45323	46613	76288	50025
Thiamine Triphosphate	Reference standar	10541	5624.6	16207	5523.6	14993	5137.7	11482	4213.8	10423	6438.4	10179	12344	8431	15681	9661.1	9397.4	13706	11630
N-Acetylthreonine	Reference standar	22918	18623	30649	41215	36426	53107	75035	46485	81906	38715	13057	24838	17010	43429	19813	21996	26222	13767
Hypoxanthine-9-β-D-Arabinc	Reference standar	12209	413.59	10921	26797	38276	49849	42437	14393	117110	32036	39989	7192.7	14442	16624	13028	12033	12949	15313
[illegible]	Reference standar	18418	3079	18370	10535	4948	15803	4759.8	28192	7024.4	33757	1994.5	6763.4	2468.2	14658	7441.2	7337	11215	13850
Oxoadipic Acid	Reference standar	147940	308840	191980	430140	117820	403370	238590	192570	406070	230790	140600	209790	331920	200180	113010	135950	137390	282420
3-Methoxy-4-Hydroxyphenyle	Reference standar	20205	24053	2616.4	108410	2990.2	178570	94579	112660	118050	105460	5085.2	1265.8	7355.7	2802.1	3671.6	2043.4	4669	56844
1-Methyluric Acid	Reference standar	5687.6	26275	49468	18274	8010.8	34856	74093	23967	10880	12374	9178.4	18731	10855	11877	11851	12462	9075.5	13206
Aminomalonic Acid	Reference standar	46745	57599	17467	717.93	15797	345.89	135240	15513	31233	23262	17514	345800	22365	426890	20844	42964	25838	333420
3-Methyl-2-Oxobutanoic Acid	Reference standar	23511	16377	28663	20554	24641	24478	32218	15947	30039	23053	11591	24303	16253	40698	18456	13322	22331	26030
Allysine	Reference standar	27863	22593	30234	29462	29350	47390	109770	46020	67240	41435	21616	40623	32434	29878	28851	27593	22892	50531
N-Acetylmethionine	Pulic databases	1661.7	1865.5	6.1748	12441	1377.6	877.73	5649.6	1726.5	13418	1982.1	1503.5	2177.9	1577.2	1025.5	2094.6	854.09	1621.1	1810.5
Ribulose-5-Phosphate	Pulic databases	1818200	11186	25274	19989	33942	16403	49035	19989	19.357	19600	13798	25368	21526	22510	25767	21292	18493	20906
Argininosuccinic acid	Pulic databases	52558	59792	73813	56647	63784	63128	108350	47517	40220	52545	65042	64508	68456	55714	80863	40178	47834	55210
2-Deoxyribose 1-Phosphate	Pulic databases	124720	135390	153720	120700	112610	139740	102190	159070	51560	155580	74365	109350	105080	148990	175900	100300	127350	112810
N-Acetylglucosamine 1-Phos	Pulic databases	110130	184640	116900	156490	80560	218660	264690	210790	232270	222120	307420	107450	146670	85159	143700	62435	175960	149010
Estrone	Pulic databases	270910	154970	86553	463450	64441	621820	226030	382730	1192500	250820	57344	158710	195470	152580	204000	257790	240420	255180
(3-Methoxy-4-hydroxyphenyl	Pulic databases	34493	42778	6886	120300	11561	182080	361540	136450	401270	91029	18648	25785	24650	61818	9567.6	24607	13470	50388
Xanthosine	Pulic databases	84411	47384	109900	86991	71740	127140	169610	98217	251030	98241	67850	83088	52270	90497	58848	59403	165160	65597
estrone 3-sulfate	Pulic databases	0.30614	0.3598	8.4406	1152.9	10582	1.1144	3703.1	64963	19017	7360.3	55806	9052	36562	7165.6	5540.5	79731	59550	19807
UDP-glucose	Pulic databases	130340	2117.1	13155	4813.8	3885.1	11055	5497.4	16294	15757	4920.5	7598.5	7955.5	11555	12609	3891.1	16660	24098	9033.5
3'-Sialyllactose	Pulic databases	26779	23301	8927.9	15100	12072	28887	21926	15501	87278	10040	14898	15718	13676	7944.5	4150.8	8617	1196.9	11537
1-Methylguanine	Pulic databases	9288.2	4506.3	10424	16003	11055	17889	24654	11871	13696	8469.9	7014.8	5692	13901	8917.5	3786.7	6156.5	7621.6	8982.7
DL-3,4-Dihydroxyphenyl glyco	Pulic databases	505620	24996	70491	88659	47615	15405	51290	5200.6	56423	50155	47205	18098	46082	27365	43444	1193.6	46891	1194.9
dihydrotachysterol	Pulic databases	928740	749940	117490	579770	845870	308680	1134200	103670	120080	277370	380890	9834900	660920	4617700	458720	2247400	732150	3668100
Indoleacrylic acid	Pulic databases	66059	72527	98451	52113	59111	32059	72730	17845	73668	15203	435980	71453	180050	19501	78474	26317	77996	15092
2-Methylbenzoic acid	Pulic databases	69805	33561	870.66	43495	84815	34328	108200	51982	338290	35539	138270	17612	85658	86518	47472	31663	52910	46583
Hydroxyphenyllactic acid	Pulic databases	637480	232110	550560	439210	555490	812690	1717500	537210	2326600	577060	568360	441730	249390	465480	356400	421700	260640	501090
INDOLE-3-CARBINOL	Pulic databases	40315	64163	14513	52309	31165	53712	64542	71848	57326	39661	43744	41616	25041	68392	52772	70514	68193	127760
L-Erythrulose	Pulic databases	31320	26150	16074	45324	19618	53176	39207	65654	47864	56589	31174	9868.7	18814	13661	19752	14840	16717	12857
N-lactoyl-phenylalanine	Pulic databases	139970	33653	123620	99177	283650	91773	766490	182110	391070	109860	135130	98647	111700	138180	39742	77764	43897	149410
Cyclamic acid	Pulic databases	0.89849	65572	1.3129	0.97593	1.2285	3376.2	854.39	18241	1.1163	2431.4	1.193	0.82771	148880	639.48	1.6636	1.4107	1.1027	434.9
deoxyguanosine 5'-monophos	Pulic databases	175860	55624	159890	56935	156880	69459	156670	210130	627790	98733	130430	33258	124180	128510	64437	126450	154490	61623
5-Methoxytryptophol	Pulic databases	334090	218370	282310	347850	607970	414940	1006200	180980	828270	197880	438020	343920	159470	275360	207330	254240	139210	247770
Uridine triphosphate (UTP)	Pulic databases	2764.2	3281.1	64.891	4522	85.684	4006	826.11	27435	52760	5168.3	162.63	1594	208	3578.8	90.949	3440.6	61.296	2391
6β-hydroxytestosterone	Pulic databases	6594.8	4069.1	6483.1	17675	6915.9	4040.6	7605.1	2596.7	3804.7	2665.6	4904	7300.3	5432.2	3384.6	6802.1	4623.4	5807.2	3951.4
Carbamoyl phosphate	Pulic databases	1253700	258050	65129	18842	28108	118400	74585	125940	59483	50902	55099	16368	63756	57047	28265	98112	103870	61356
Indoxylsulfuric acid	Pulic databases	60387	101990	4.4151	101280	4.0427	126640	3.9602	190830	6732.7	64596	2.9575	1285.5	3.0534	3003.5	1829.7	418.39	2518.7	560.13
Salicyluric acid	Pulic databases	10944	7476.8	3180	19363	8031.2	33591	21676	17488	187950	14006	17750	21159	24660	13718	20960	19868	12603	50829
Ginkgoic acid	Pulic databases	9675000	9950300	9130500	11530000	11237000	8087800	9140900	9488000	7833900	10158000	10320000	9031700	9459200	12082000	10713000	10788000	10367000	8682400
Hydroquinone	Pulic databases	896.13	1739.8	334.13	112.21	0.23409	85.258	250.07	76.2	0.21288	41.63	485.59	146.51	1334.8	276.49	0.35835	243.02	0.23972	134.93
Anthranilic acid	Pulic databases	31933	54060	12103	91737	16994	4810.1	11630	5827.9	10099	3333.1	12141	3460.4	13400	1893.9	8557	4640.3	15935	6253.6
Succinic anhydride	Pulic databases	3515400	3474800	3549300	4745800	2748400	4717100	4029100	5130700	3208300	5431300	4081600	2653900	4020100	2855400	3491900	2588900	4242500	2491700
3-Methylsalicylic acid	Reference standar	18189	26380	14891	26168	17473	21710	15598	22250	15245	25407	14400	17000	13815	18891	16334	18243	17744	21063
Indoleacetaldehyde	Pulic databases	1755100	1709000	1498000	1402100	1388300	1487300	981120	1684100	1459400	2197200	1543600	1758500	1539000	2715200	1532900	2453500	2180900	2677100
D-Xylulose 5-phosphate	Pulic databases	511860	655490	142220	246120	112150	264770	199840	248870	171080	455140	152090	226330	199220	214440	135260	193410	279940	195240
Vanillylmandelic acid	Pulic databases	9633.5	23880	24117	31930	23122	26328	20130	30098	17499	27268	32057	31961	34795	27371	37315	43605	30208	40915
O-Desmethylnaproxen	Pulic databases	245510	321040	210630	241120	300290	154470	341570	316400	221050	269420	378230	293090	204280	277500	351360	342650	298270	183750
Ethylsalicylate	Reference standar	80356	49625	68717	58501	69157	62470	49854	45649	47761	63266	43516	40370	48198	61936	43155	61745	51766	77372
Maltol	Reference standar	36.496	11244	1869	1048.5	3462.4	2140.1	4553.8	12871	3172.4	4562	1261	728.74	1843.6	3255.8	975.36	2278.1	1338.2	683.38
2-nonanol	Reference standar	472330	478030	412630	515260	421830	481640	346650	472440	420570	480760	405680	457070	457500	492520	480390	477360	451480	475400
1-Naphthol	Reference standar	6490.5	7585.2	7542.8	6009.9	5111.6	6319.4	16.923	7074.3	4822.8	7587.3	9009.7	6671.8	6577.3	7204.8	8099.6	6570.1	7518.7	6136.1
2-n-Pentylfuran	Reference standar	7890.9	8579.4	11278	24214	11678	16071	10784	9933.1	10418	10836	7204.2	6835.8	6381.6	7530	10197	7390.2	11049	8528.8
2-Methyl-5-nitroimidazole-1	Reference standar	6408.8	3633.5	8641.6	31899	10995	22866	27585	23576	15623	13893	16787	12550	34794	8163	16890	26597	29515	13665
Aspirin	Reference standar	1685300	1622600	1563500	1806900	1319100	1827100	2514000	1972100	1267100	2348600	1574000	1519800	1560000	1646900	1399000	1407200	1723500	1594400
Sorbic acid	Reference standar	219890	172620	156550	262960	190290	265150	245930	317290	255390	261580	227130	318980	173240	253790	165150	242830	211330	316590
Phenoxyacetic acid	Reference standar	24.953	4127.6	2.8812	235.79	1296.1	364.05	4054.9	541.88	1573.7	562.35	721.4	1623.2	2.414	650.96	3.5792	476.73	950.13	857.82
4-Ethylphenol	Reference standar	34867000	11888000	785260	1713900	747260	2075700	770220	200620	802910	2460200	586220	2193300	705740	1899400	825060	180920	747740	139740
4-tert-Octylphenol	Reference standar	511060	470660	533150	577370	536540	522330	578460	497490	509360	475960	520390	478180	508370	507510	460250	514470	500960	484750
PHENYL-BETA-D-GLUCOPYR	Reference standar	389930	688390	513950	695080	296190	794540	448500	789970	749770	771830	326220	356360	422170	423390	306860	402820	350840	527270
2-(Methylthio)ethanol	Reference standar	38466	40221	45942	186340	52373	58542	39803	38101	38174	66018	30243	52176	35363	77093	26745	69953	35815	158080
4-Hydroxybenzyl alcohol	Reference standar	3369.3	2346	5565.4	18873	4993.3	3662.3	8589.9	1529	20838	1606.3	4926.4	2140.8	4477.2	55201	3572.3	3621.2	4550	5932.4
2-Nonanone	Reference standar	126450000	124160000	117270000	131570000	119160000	127040000	105130000	130700000	130790000	130050000	130150000	126160000	132940000	131640000	121510000	126970000	133340000	126010000
Propylparaben	Reference standar	20123	65086	16191	27663	18101	17462	11780	20561	9299.3	20307	13096	19619	15081	16179	23345	23105	19407	40518
2,4-Di-tert-butylphenol	Reference standar	36644	36866	32924	43393	39997	37968	36733	39865	41101	39251	40157	37633	36960	37792	38338	38452	36709	33166
Methylparaben	Reference standar	56502	214880	64178	62663	61891	52682	52095	55258	56108	53447	54017	46607	50798	50690	61979	119560	56099	127740
Taurodeoxycholic acid sodium	Reference standar	16327	13902	6833.8	77707	184820	262030	15000	192020	50533	22191	12169	16042	25392	10656	3936.7	7957.9	26158	10922
Xylose	Reference standar	124080	86268	44933	199150	52072	185100	168680	303630	297720	199410	108900	48911	130370	37165	91781	38992	73131	51065
4-Hydroxyhippurate	Reference standar	5306.2	3752	1876.8	11443	1027.6	21698	7150.9	9439.1	74833	8411	7691.2	10097	7886.5	6183.7	12631	12248	5569.6	27168
N-acetyl-beta-alanine	Reference standar	403510	354100	404680	582220	418210	651640	953620	748610	592030	604280	374380	320500	306170	419640	335030	279760	299780	344830
Scyllo inositol	Reference standar	23230	44633	44550	91817	53820	117850	165890	123560	574450	162370	117440	30194	117890	58817	60221	50345	23656	61163
TRP-GLU	Reference standar	5201.3	688.68	12198	1.4365	1817.5	3130.4	36180	12317	2464.3	3494.2	4668.3	8668.8	11787	27451	7973.1	5578.5	5573.6	1759.2
N-acetylornithine	Reference standar	376970	336810	532100	162350	153810	91199	544510	380210	311480	295230	92849	109980	334490	444230	637530	693730	552320	337920
N-Amidino-L-Aspartate	Reference standar	12280	120530	24398	8996.3	8877.2	39265	42572	5452.1	24369	69554	16013	11734	15547	33851	11313	19705	18726	2952.9
Dethiobiotin	Reference standar	140.89	234.01	0.31473	166.83	0.29294	128.11	0.29165	128.27	1012.5	252.48	214.57	151	0.26803	85.296	0.36492	80.179	0.24272	83.664
Mono-Methyl Glutarate	Reference standar	10365	20047	13154	29372	14774	21860	17768	28181	14607	25832	13258	26406	12001	25364	13547	32848	15538	24253
Phenylpyruvate	Reference standar	2646200	562620	119090	454840	150160	31490	175100	325570	18446	418050	141350	76377	87806	1114700	85879	1099700	53776	50710
D-Homocysteine	Reference standar	865240	625020	734860	946310	1493000	1069700	2647600	544160	2176200	532800	1079700	798430	411760	694200	517440	641920	360910	592040
Glycine	Reference standar	63641	81999	52402	86451	71345	92122	74251	63908	92605	103080	80693	65250	80568	56244	66088	90278	69461	83196
L-Tyrosine	Reference standar	99487000	30373000	90822000	83219000	62774000	119400000	86614000	103270000	121550000	78479000	86219000	59172000	62520000	62913000	62075000	91409000	49158000	74978000
L-Lysine	Reference standar	21821000	16786000	31626000	11674000	15729000	19029000	37954000	26966000	36526000	26856000	17544000	11547000	12252000	13671000	17109000	17089000	14946000	17779000
L-Ornithine	Reference standar	972790	1050900	2043000	792940	1083300	1650700	1480100	1736100	2029800	1646200	1632500	897250	1112600	990060	816680	1069900	913030	1502600
L-Alanine	Reference standar	5785100	3062100	2831200	2335800	3434900	3394600	5612200	3888500	3410600	4178500	3055300	3350800	4137200	2887500	2772800	4144000	3535000	6243900
L-Histidine	Reference standar	5934600	5545100	7708400	4151100	4842200	4970400	15200000	6110400	8821200	7131500	5148800	5949700	4313000	5796800	5686400	5458200	6416800	7444300
L-Leucine	Reference standar	185270000	240150000	181980000	84162000	112490000	162180000	286820000	116410000	224890000	163600000	151660000	130300000	107320000	198750000	138010000	157550000	120580000	157900000
L-Methionine	Reference standar	8939000	5177100	9079000	3516200	3985400	5688300	8898000	8390100	10487000	7412900	5302400	4880800	4143400	5619900	5230800	5727500	5300800	4659900
L-Phenylalanine	Reference standar	343570000	292110000	412000000	325710000	362840000	327480000	421650000	313750000	367130000	336680000	355760000	350940000	284410000	335930000	250780000	279230000	220160000	296080000
L-Proline	Reference standar	55662000	40717000	39499000	37368000	37295000	62154000	69794000	54945000	85770000	34234000	37921000	24108000	49415000	28925000	70980000	52652000	76159000	54007000
L-Tryptophan	Reference standar	102570000	104340000	77695000	59117000	75853000	79359000	61678000	66642000	92879000	73762000	94756000	86489000	67149000	93020000	99445000	112980000	90966000	123530000
L-Valine	Reference standar	115890000	118710000	106570000	83624000	83230000	120520000	152500000	83778000	114440000	98496000	112610000	77158000	68943000	128130000	94066000	83772000	80549000	100010000
(5-L-Glutamyl)-L-Amino Acid	Reference standar	23230	4609.1	34281	44301	23384	5680.6	44218	27888	11323	4625.1	33287	59732	34604	26639	17466	27177	27994	81968
3-Chloro-L-Tyrosine	Reference standar	15636	22029	32139	19211	17918	21319	19806	22640	13296	22994	16553	16621	19340	21710	17333	16860	17020	20042
4-Hydroxy-6-Methoxycarbo	Reference standar	9834500	9859400	10089000	8379600	9061100	8815800	10412000	8592900	9205400	8433800	8968300	10250000	9723100	10272000	8889000	10065000	9614500	10624000
5-Hydroxy-L-Tryptophan	Reference standar	335040	351880	269500	220930	247150	253620	166950	242120	259410	375360	229460	251770	290180	495300	295210	435610	403460	474680
5-Oxoproline	Reference standar	950110	652990	505040	560680	697430	648650	569120	347060	396120	545530	859240	590940	759960	601930	761650	791650	924230	520660
Asp-Phe	Reference standar	91247	571080	9927.4	13719	7797	6956.5	38085	153130	40395	122220	16352	17413	13919	25954	34822	6145.3	19019	183930
Betaine	Reference standar	21534000	27472000	26017000	25713000	28482000	16624000	26300000	21962000	28952000	7802900	16346000	25486000	27873000	14313000	16803000	25177000	20224000	18531000
Glutathione Reducedform	Reference standar	36193	36889	8185.6	15051	9572.6	26860	12206	12504	13410	15948	13559	35474	26133	48979	12820	35589	10629	47296
L-Cystathionine	Reference standar	36476	15344	14332	18818	15386	17452	31730	22288	37461	26876	17038	15650	2317.9	7874.9	1648.7	2599.5	7228.5	1576.8
L-Dopa	Reference standar	49697	80872	12746	12891	13058	27520	12640	287150	43130	86589	15975	4787.1	27136	8931.7	12584	6663.5	19913	23626
L-Saccharopine	Reference standar	1296000	1027000	1086400	692400	1179100	901230	923530	761020	1046100	723080	976240	1223500	1263600	1230900	795290	1178200	1283300	2502600
L-Theanine	Reference standar	475740	557900	814170	361980	168720	196650	612570	808320	570170	403310	235190	168380	679040	736530	576430	801880	819050	293320
Methionine Sulfoxide	Reference standar	376090	429280	444460	278540	441260	274010	486800	339020	346210	379480	319420	365610	296130	438610	298120	523600	272590	395760
N,N-Dimethylglycine	Reference standar	4066400	2958800	5013500	2571600	3714100	4767700	4589000	4496900	4482900	4316300	2644300	2608400	2430200	3415100	2235000	3423200	2735200	3111000
N6-Acetyl-L-Lysine	Reference standar	170940	171070	224580	269390	263480	266400	492760	112560	220970	197890	290670	153710	294740	194400	382530	132060	273020	287140
N-Acetyl-L-Tyrosine	Reference standar	68572	15245	19115	35942	24079	31600	99357	20612	108540	33550	20510	50244	17701	28227	23973	34102	25849	26045
N-Acetylmannosamine	Reference standar	83142	15736	27233	15019	37341	51934	52778	41261	43747	59243	24362	43961	28505	212360	21215	38704	26599	50386
N-Glycyl-L-Leucine	Reference standar	119000	33664	17006	124290	15690	60555	77130	58471	38285	22828	40373	53677	29483	34339	21319	96843	36406	91533
Nα-Acetyl-L-Arginine	Reference standar	1516700	465480	40415	216840	69336	8258.1	29635	79377	37920	123590	55867	12190	49162	7923.5	102550	36154	82858	19248
Phenylacetyl-L-Glutamine	Reference standar	5737400	8448500	3562000	9453800	1006300	8006500	10876000	18673000	17130000	6314800	4711500	1226200	2339700	7321500	3839200	991150	5970700	919590
Phe-Phe	Reference standar	36421	119010	14044	10723	55712	10113	81493	2980.5	14420	2399	63447	4146.4	27363	7150	32774	2363.4	30272	5580.2
S-(5-Adenosyl)-L-Homocyste	Reference standar	126800	23582	26231	29494	40317	70873	316580	37146	891470	38711	28802	31092	24216	12893	44039	4865.9	3893.4	12607
Serotonin	Reference standar	1051500	459040	54353	52024	13.465	36373	28.574	376740	15.924	49576	72278	271730	176580	21352	84306	388130	615760	426110
Urea	Reference standar	44585000	34567000	44211000	36487000	30402000	50867000	56393000	62235000	33234000	83021000	41993000	21088000	17923000	36406000	34229000	30156000	27816000	26907000
L-Alanyl-L-Lysine	Pulic databases	1340700	34363	190020	137400	177190	197670	268980	112050	130680	112740	168140	94797	79955	86682	111270	72229	62856	114110
Methyleugenol	Reference standar	8421.1	11212	6197.1	9737.2	8861.7	12949	7731.6	11331	7921.9	12673	11521	5384.9	7323.6	20952	9980.7	27824	10581	24696
P-Coumaric Acid	Reference standar	4961500	3403900	3953800	2986000	3239500	4676700	3629000	4038200	5616000	3200100	3817400	3208900	3021300	3522700	3182300	5321100	2752000	4291500
Syringic Acid	Reference standar	8140.9	7800.2	12926	3628.7	6.3	8437.5	12139	39556	18722	26072	3959.8	2463.9	8.3941	5914.7	8.8348	2.7922	5794.4	5714.3
4-Ethylbenzoic Acid	Reference standar	46951	42319	45548	39606	42020	30757	31547	31572	42120	47514	53873	36579	38711	42403	49547	36641	41168	34707
4-Hydroxybenzoic Acid	Reference standar	3112200	1023300	141740	107070	116510	113070	123680	117310	100750	104780	128940	99872	136380	127890	135800	119160	114380	111250
Phthalic Acid	Reference standar	19008000	18164000	19601000	17474000	18360000	17339000	19711000	17147000	18231000	18192000	18527000	18788000	17355000	17694000	16092000	16600000	15783000	16627000
1,4-Dihydro-1-Methyl-4-Oxo	Reference standar	7734200	1746700	1788600	3148800	3494200	3979100	11158000	3545800	6898100	6097000	1864300	1641000	1204600	6675400	2132700	2725500	2191200	2646600
4-Pyridoxic Acid	Reference standar	182780	150040	21378	127170	20419	91102	320900	117710	286050	138960	28291	127080	27226	96941	33858	159080	26242	137960
Theobromine	Reference standar	17.905	59966	1162.4	59.018	0.53665	182.7	1.135	162.89	0.63044	79.195	2857	197.73	0.7355	107.96	125.26	222.79	140.32	87.163
Acetylcholine Chloride	Reference standar	189760	79434	177160	155310	122920	288680	301740	248850	242690	304330	118000	92498	194130	150860	119020	126620	235930	148860
Choline	Reference standar	446400	284060	533860	193830	509620	342410	350660	400790	550030	254150	314870	258140	329650	290080	286540	381340	292330	338610
Putrescine	Reference standar	19481000	10618000	910460	920600	858410	2552100	1003500	2557200	999860	2241600	665290	339720	762180	303540	840610	256650	889110	131850
Diethanolamine	Reference standar	73531	46275	69044	61947	35121	65429	59291	98814	68574	42435	42800	34899	76729	58619	50139	54181	85375	44623
4-Nitrophenol	Reference standar	10864000	10958000	9833300	9612500	8955300	8404800	7830900	8788700	9008500	10312000	10359000	10440000	10564000	10184000	9387400	10956000	10576000	11403000
Inositol	Reference standar	19532000	20074000	21118000	17509000	16519000	14831000	18904000	18509000	16267000	15922000	20405000	20883000	22806000	22840000	15713000	24518000	20673000	23505000
Myoinositol	Reference standar	20670000	20746000	19509000	15041000	15404000	14135000	12552000	18114000	16881000	18227000	18267000	17471000	17054000	19001000	16106000	20347000	20833000	19525000
Vanillin	Reference standar	3341800	737550	776400	1519100	1668300	1385100	5631300	1366900	3533400	2985800	833550	964480	565930	3776500	1027300	1604100	1010400	1240300
1,7-Dimethylxanthine	Reference standar	62.297	10714	30639	1.5901	63.758	1.1731	17.176	1.9801	15.837	0.8218	51.617	49.783	0.090692	0.60191	188.32	0.80291	0.059198	1.2179
1-Methyladenine	Reference standar	40822	23403	29079	23751	23612	46120	24375	26343	23425	7234.7	28590	27285	30041	30016	19270	33589	22872	31279
1-Methylhistidine	Reference standar	624950	491280	599680	582590	387070	575430	815520	1387000	1297100	810700	351580	364200	899690	335980	544800	253660	1264200	372580
3'-Aenylic Acid	Reference standar	3454800	1036500	1871900	777640	2442000	939690	3640500	2995500	8028800	1194000	2807700	819660	2944100	2277300	890150	3013500	4232100	1194600
5,6-Dihydro-5-Methyluracil	Reference standar	242690	174300	149700	96787	122930	190370	383650	314120	255490	320820	200440	102070	390010	168180	112630	184930	285630	167850
5-Methyluridine	Reference standar	72866	15503	43260	87505	53850	85173	126120	105020	120280	93016	39785	42608	61085	70255	60213	55349	70204	66616
7-Methylxanthine	Reference standar	3.1816	62879	18275	46610	19215	44379	31425	38656	21275	27570	15650	13790	11874	17968	17512	14648	20878	10984
Adenine	Reference standar	1737600	992510	1078200	853750	985840	1179200	2404800	1096600	1734600	809810	1224800	714130	953680	781730	865470	1228200	794520	901510
Cytidine-5-Monophosphate	Reference standar	128170	115200	123050	121690	113810	99672	150310	121500	266390	67137	96325	129480	122250	123890	88311	105080	192690	124930
Cytosine	Reference standar	89408	125190	23244	83897	34494	61641	60510	47120	40621	40323	37514	273500	58512	35718	53094	16595	70048	93639
Guanosine Monophosphate	Reference standar	27896	24129	14144	27749	11860	5831	28163	19553	51051	9827.8	11746	12378	21491	5266.7	4851.8	41968	21098	11973
Inosine	Reference standar	25137	24342	4.2102	21940	15186	6500.5	7268.4	5298.5	37776	28047	15416	12131	7689.3	17151	3349.8	12301	3260.1	6943.4
Purine	Reference standar	878290	898570	973640	777730	842820	788740	1039300	795040	860740	869770	880370	1055400	727590	908320	708000	785300	668510	737150
Thymine	Reference standar	48306	43819	37829	6868.5	11125	40508	68680	26557	39332	16416	28768	36027	69646	126620	19229	93468	98973	86699
Uracil	Reference standar	450240	662120	568650	478510	369130	380300	284220	568420	164520	441290	423310	288450	402990	585900	478650	460880	531480	518310
Uridine	Reference standar	947610	1602900	1529500	1429400	899380	970980	591230	1379700	414870	1148800	785950	583160	826180	1085200	1142000	835350	1227300	1053900
3,3',5-Triiodo-L-Thyronine	Reference standar	4420.3	5434.7	2494.5	2875.6	1536.1	2618.9	1938.5	2130.4	1773.7	1822.6	3170.3	4335.4	3553.4	2901.6	4133.5	7804	5484.7	7874.8
Norepinephrine	Reference standar	186080	124560	95738	90230	53057	62750	95481	79425	43912	69028	73327	35032	92708	110480	80144	99766	155930	128380
D-Arabitol	Reference standar	1939100	1201100	442880	62782	477520	473660	555020	18001	518560	49097	353890	88965	575310	53814	357540	119730	1150400	587070
D-Mannitol	Reference standar	5568600	918880	29528	231590	8122.2	167470	37985	248690	154590	206100	21340	169530	120660	184180	11756	102160	28435	143040
D-Fructose	Reference standar	123420000	133590000	128950000	127070000	113940000	110790000	103270000	106870000	116960000	135090000	128740000	135500000	136360000	140210000	115680000	178610000	140570000	163610000
D-Mannose	Reference standar	106570000	115830000	115220000	138980000	110780000	114990000	92732000	98999000	100770000	116010000	113570000	140690000	130980000	135820000	114110000	167730000	129800000	163310000
D-Melezitose	Reference standar	6249.1	3142.6	642.19	18639	29129	3546.2	209.15	5890	13657	1740.7	35278	1213.6	2008.9	6137	165.63	1942.9	979.24	797.48
Lactose	Reference standar	44911	6627.6	37957	41355	74865	11241	39055	6245	65669	14086	35733	10383	60226	96947	10259	131590	11269	238960
Maltose	Reference standar	160550	136060	186230	186880	167370	161650	151800	185800	183290	166010	158260	183470	189740	198410	176900	246030	190510	195120
N-Acetyl-D-Glucosamine	Reference standar	1226800	1534500	1933900	1271300	2062800	1983000	2133900	2151900	2010200	1586400	1263700	2679500	1385700	2081900	1396400	2642700	1604200	1675700
Raffinose	Reference standar	6754.5	2594.5	650.67	37151	36075	4344.4	1057.8	5757.7	11439	7501.7	31696	1095.7	2076.8	354.26	791.64	1337.4	243.15	2232.8
Gluconic Acid	Reference standar	64482	3.4275	9433.4	10738	7304.1	19615	4050.2	39623	12657	16720	5587.2	12636	15277	28992	2000.2	11691	2216.2	10789
L-Ascorbate	Reference standar	24353000	24596000	29859000	30103000	35424000	22601000	42746000	22955000	30471000	30456000	43194000	42588000	28988000	28647000	34526000	44433000	33870000	75256000
Nicotinamide	Pulic databases	1858800	756170	747520	711940	266950	659780	269390	782370	443440	334100	597040	801720	850870	958660	586500	1085000	1387200	657410
4-Oxoretinol	Reference standar	1387.8	3168.1	2182.4	2104.8	3365	4954.8	2029.4	3870.9	2327.3	2430.1	2821.5	4036.6	3063.9	2212.4	2382.9	3398.2	3173.3	2372.8
Nicotinic Acid	Reference standar	28169000	168170	326630	219820	239250	364820	299070	461650	518440	283420	346550	220320	271930	332650	275880	262690	212630	259130
Nicotinuric Acid	Reference standar	502820	154750	281840	112860	193410	387380	1014000	641920	1159800	673200	504420	100860	162000	318380	201430	175780	134140	229570
Pantothenol	Reference standar	4066.2	15.738	2451.5	362.94	554.48	930.79	2377.2	1957.8	1198.9	866.15	1015.4	3234.2	1377.7	1447.4	499.59	2800.5	928.61	1993.3
Riboflavin	Reference standar	290870	190520	160680	306090	148100	285870	156960	236720	84086	278320	473160	226050	301560	301720	300930	312170	273130	332660
Trigonelline	Reference standar	9892.5	9690	43348	59945	50313	16838	81458	140630	11830	38735	92406	46854	151780	16224	140410	121470	49868	42692
3-Indolepropionic Acid	Reference standar	23553	19653	1.4827	6768.7	0.87903	1680	1.8902	2.0752	507.83	0.57124	0.6833	1824.1	1.548	0.38531	385.53	0.38428	1.0203	0.76361
Indole-3-Acetic Acid	Reference standar	2411200	2073700	1822900	1803300	1828400	1883400	1177300	1962400	1650800	3219100	1886500	2262000	2275800	4165600	1890600	3573100	3000600	3864700
Methyl Indole-3-Acetate	Reference standar	16552	11788	2076.1	1756.9	4453.7	6356.2	12142	1522	14205	10691	4876.3	2788.5	3003.2	2268.4	3093.6	2083.6	4863.4	2993.4
3,4,5-Trimethoxycinnamic Acid	Reference standar	19193	19634	6407.6	33076	3221.6	39736	12291	39601	27.505	32654	5733	25947	4274.6	21190	29.831	80316	17.953	76830
4-Acetamidobutyric Acid	Reference standar	323670	257260	284790	708140	272650	1478600	784220	873540	1892000	315820	257310	210800	307740	385440	262070	428600	242550	388440
4-Guanidinobutyric Acid	Reference standar	4024000	1701500	4681600	3418500	3419500	5879400	5019700	5609100	5438300	6609600	2317300	1975800	2952700	2726600	3646000	2455400	3841500	3031200
5-Aminovaleric Acid	Reference standar	281510	232820	263680	100280	227760	86776	215660	334200	243030	66637	216600	139570	203870	102870	358810	412740	279820	383180
6-Aminocaproic Acid	Reference standar	35849000	25122000	25382000	15754000	13698000	32378000	36087000	19936000	46949000	26015000	23449000	19336000	23079000	33986000	21645000	22972000	26018000	27728000
7-Methyluric Acid	Reference standar	256490	197540	254370	210680	215440	242920	216260	220930	259140	200160	202680	214840	207090	211870	255470	210630	217030	211120
Azelaic Acid	Reference standar	174230	125910	162890	141760	143040	148260	140090	163470	129460	176920	151540	126380	178770	141930	151550	146890	127000	149910
Chlorogenic Acid	Reference standar	59479	2081.1	1804.9	4.2712	1943.4	1219.8	1929.8	3512.2	3572.8	1835.3	5253	4545.8	2940.5	2530.1	2400.3	6499.3	3984	10372
Creatinine	Reference standar	1096200	720890	1332200	1769400	1017400	1754800	2528200	1962400	1803200	1328300	705530	1061200	606700	838660	815660	687970	636310	893830
Dl-2-Aminooctanoic Acid	Reference standar	306000	728550	155900	522850	121840	520050	100820	446020	156260	247480	310020	258480	712630	104890	515090	1290200	604530	965220
D-Pipecolinic Acid	Reference standar	524080000	113210	2028700	304050	930330	396100	1782900	506020	2486000	450660	409920	585130	734820	625900	685860	802480	335970	595880
Guanidineacetic Acid	Reference standar	16485	13116	28754	10651	8653.2	14335	14422	21771	4497.1	8662.7	7947.9	7416.8	15013	28305	25571	39214	17487	41342
Hippuric Acid	Reference standar	1043200	788720	10780	271240	13746	1002900	216090	136060	2487900	128830	161440	196470	149450	344330	33769	65108	30832	1852300
Homovanillic Acid	Reference standar	9572000	7355200	8516000	6404100	7016800	9581600	7799000	8947200	12734000	6737300	8798500	6518900	6845600	7052800	6399300	10873000	5734100	8137700
Hydrocinnamic Acid	Reference standar	33097	23761	58381	29315	16942	37939	6015	36887	8864.6	22909	24593	17836	26447	19859	18747	23317	17673	11488
Kinurenine	Reference standar	3260900	2072900	2727900	3336800	5326500	3631400	8958000	1904300	7669500	1976500	3556800	2737500	1446400	2143200	1770800	2124700	1261800	1919100
Kynurenic Acid	Reference standar	310510	200990	194930	380630	219830	628750	152570	1072600	233260	615600	227170	134280	156170	140760	256760	102660	250510	87470
L-Homoserine	Reference standar	2196700	2015000	4069600	1428200	2449500	2189700	2710100	3752100	6099000	2392000	1301000	1462700	1763200	1481300	1875900	2850700	1368600	1968200
Maleic Acid	Reference standar	3158200	2037600	1977200	1927000	2052700	3531700	3454500	2705700	4404100	1884900	1949300	1235000	2620400	1590600	4089100	2566700	4350900	3027500
N'-Formylkynurenine	Reference standar	168350	102420	21570	4284.4	28572	3469.3	35525	33560	29184	20263	7274.3	73445	4529.8	125890	5341.7	31904	4229	196750
P-Aminohippuric Acid	Reference standar	28358	20374	8726.1	10274	9501.6	34746	67148	44330	140920	24582	8904.9	10686	3391.9	5428.6	4608.7	8701.1	2056.4	9376.3
Cinnamic Acid	Reference standar	4241600	3000600	4741500	3530900	3939700	3235200	5186800	3181300	3892700	3950600	3896000	4689700	2694000	3748400	2374400	2715200	2001400	2978300
Uric Acid	Reference standar	24116000	20827000	21788000	25197000	9946000	16026000	21727000	28274000	10035000	28923000	16190000	14949000	19794000	14614000	21309000	14630000	25184000	15912000
4-Hydroxybenzaldehyde	Reference standar	7824600	5845400	6705900	4952000	5584400	7450600	6061800	7066800	9098500	5043200	6293200	4475800	4976300	5341100	5271700	7958700	4721700	6250900
Taurocholic Acid Sodium Salt H	Reference standar	2318600	2741300	2523800	2691800	2862600	2358000	2731500	2607600	2618400	2319600	1926900	1622200	2430900	1874700	2371000	1661000	2405100	1751300
2-(Formylamino)Benzoic Acid	Reference standar	42091000	29768000	49906000	35208000	40093000	32873000	52284000	32967000	40966000	38116000	40683000	45174000	28857000	39653000	24580000	27139000	20218000	29622000
Acetaminophen Glucuronide	Reference standar	16743	5880	2834.2	11711	6275.5	18581	22628	17545	40824	15091	7694.6	7259.3	3903.2	7478.1	5328	4452.8	5439	6428.8
Cyclic Amp	Reference standar	75943	111290	54023	150410	85478	111690	510990	52346	156150	45258	124360	119230	134440	31982	72198	22466	28714	27722
N6-Succinyl Adenosine	Reference standar	512010	599410	433380	641660	291080	924280	1562000	716950	985280	677700	405300	407270	497300	385050	398560	525440	466710	557380
3-Hydroxyhippuric Acid	Reference standar	61622	55858	51147	489420	65271	256710	232180	121710	1734200	87814	148810	125280	127720	74028	182010	140970	105420	467700
2-(Dimethylamino)Guanosine	Reference standar	1156500	1075100	742480	1285200	921420	2175100	4055500	1342100	5841300	1123800	713920	1037800	808870	1126100	911170	715790	935740	770920
7-Methylguanine	Reference standar	93216000	91419000	111800000	79575000	90189000	77017000	122730000	75272000	89433000	85703000	90241000	107270000	62264000	92948000	54658000	63940000	46271000	68209000
B-Pseudouridine	Reference standar	2184000	67804	134910	232900	126810	239890	258320	256060	298160	168830	110800	144080	157750	123500	113630	157420	191060	203050
Isoxanthopterin	Reference standar	2572200	51764	57282	224130	46464	98263	155990	3439.4	110290	94280	48474	891720	62048	416160	54253	27631	52571	811240
H-Homoarg-Oh	Reference standar	190450	187520	138020	104360	49266	114040	106180	227260	63301	194930	66935	88783	56260	81537	138410	220180	110740	208820
Pantetheine	Reference standar	19665000	14523000	17118000	14810000	17417000	14720000	17671000	15605000	17671000	14878000	13854000	13751000	15297000	14851000	17092000	11278000	18236000	11810000
4-Hydroxyretinoic Acid	[illegible]	[illegible]	[illegible]	[illegible]	[illegible]	[illegible]	[illegible]	[illegible]	[illegible]	[illegible]	[illegible]	[illegible]	[illegible]	[illegible]	[illegible]	[illegible]	[illegible]	[illegible]	[illegible]
L-Pipecolic Acid	Reference standar	736420	933940	712770	773360	891360	1281000	744870	589030	525630	771420	1077200	1009900	938710	1078500	903430	597960	1097500	632540
5'-Deoxy-5'-(Methylthio) Ade	Reference standar	189250	56289	4362.3	40421	11357	12397	521110	6874.1	6562200	7014.8	4666.4	3709.7	4028	4611.5	5912	3621.3	3539.3	4046.5
Sarcosine	Reference standar	8496300	8993600	1232800	1330000	1217300	11165000	1202500	12314000	1685000	92138	1809000	291040	1053200	304880	1227300	144220	1090600	566950
3-Aminoisobutanoic Acid	Reference standar	131090	59191	146680	51748	79697	86062	93861	106080	115960	76186	80671	76982	63756	77010	96148	79501	100740	98431
Cys-Gly	Reference standar	33248	63567	7629.9	60795	12138	22389	11953	38478	18017	72468	146320	65167	78253	25361	44503	81902	40980	43560
Trans-Citridic Acid	Reference standar	1196700	1334700	1129100	1096100	1196100	1090100	968130	1109300	1066900	979540	1478000	1333100	1503500	1310100	1136900	1294700	1383600	1294500
6-Phosphogluconic Acid Triso	Reference standar	27131000	12379000	21558000	14758000	12111000	9169900	20729000	8682600	15630000	10929000	7463500	8860200	16930000	11981000	19922000	7386300	19162000	6745000
Phosphoric Acid	Pulic databases	1.0982E+11	72470000	29288000	26657000	23982000	28189000	88728000	32762000	28667000	28784000	34649000	37324000	32284000	36778000	26439000	32392000	27578000	38150000
2-Aminoadipic Acid	Pulic databases	9724600	48957	165500	177590	140490	218930	285370	323180	530910	343020	187150	62265	100010	144020	118770	110480	102710	213120
Hordenine	Pulic databases	406020	286250	382130	357610	341200	353720	419730	363890	358070	391040	370500	457780	322500	410200	270010	263570	262410	285750
6-Methylmercaptopurine	Pulic databases	82691000	78272000	85782000	78289000	80241000	76661000	86102000	74705000	78927000	79662000	79947000	81264000	74930000	79476000	67149000	73195000	67337000	71473000
Ergothioneine	Pulic databases	14533000	442830	352970	65106	338400	103170	480580	75061	408260	43619	181250	387340	92647	375040	256170	173590	152420	211890
N-Formylmethionine	Pulic databases	2950.8	3269.1	1494.8	4962.8	6.903	7047.5	14.635	2934.9	8.1479	2245.4	4.4267	6659	3259.6	3759.4	1661	9583.6	8812.2	634.03
Methoxyindoleacetic Acid	Pulic databases	1963300	1850300	1442400	1015100	1407200	1392000	1096900	1182800	1640100	1331800	1793600	1619400	1212800	1707300	1881700	2371400	1602000	2453800
Sn-Glycero-3-Phosphocholine	Pulic databases	101720000	948070	640260	296000	731790	725650	529450	416420	2201100	296620	886610	1092900	2038700	1462800	470530	2890700	1231700	3698100
3-Hydroxykynurenine	Pulic databases	23987	29755	17338	11258	6989.6	23195	7879.1	18185	33819	8685.9	4323.5	8661.4	3570.7	25164	1869.1	11518	3317.8	13663
P-Aminobenzoate	Pulic databases	17415	18319	5861.9	7962.9	4847.4	9673.8	84591	9232.4	24727	57792	8964.4	58631	21683	16649	3705.1	17030	6303.4	139460
Indole	Pulic databases	2001200	2415200	1700800	1197900	1830100	1593200	1909900	1366600	1747400	1526500	1755700	1804000	1846200	2116000	1806900	2617300	1963800	2745600
Isoquinoline	Pulic databases	50281	55998	11041	44528	30192	41997	56902	52636	53180	33200	38268	45413	20941	59472	45635	69474	54103	141560
Piperidine	Pulic databases	7144100	4900600	5122700	3005400	2540000	6598200	6561800	4027300	8234300	5467700	4506100	3758200	4567000	6928900	4259800	3988900	5224600	5628000
5-Aminolevulinate	Pulic databases	838020	538210	606910	258950	335880	797720	710820	381610	1036300	532300	494620	403630	516750	796080	490100	512500	635900	646390
2-Methylguanosine	Pulic databases	633450	574300	658460	392380	476980	513670	1437800	655710	986170	432400	435280	625710	633120	1025300	528610	688250	643840	650050
Glucosamine	Pulic databases	326290	416570	324050	703850	378880	498030	482100	63703	275770	644770	197710	288230	359700	810850	388590	796670	248710	525570
2-Hydroxycinnamic acid	Pulic databases	31552000	20562000	25573000	19606000	20313000	30015000	25612000	26403000	37548000	20579000	23939000	19719000	18835000	20349000	19034000	32166000	16619000	25680000
trans-3-Indoleacrylic acid	Pulic databases	20829000	20448000	14780000	10428000	15472000	15307000	12226000	12300000	19704000	13721000	17808000	16975000	12483000	18950000	19672000	24472000	17380000	26418000
DL-Stachydrine	Pulic databases	1011300	550810	245030	2635800	3191500	3085700	620510	3027000	280880	3238300	822290	2497800	869240	2297400	352190	2349900	1905900	2191000
L-Norleucine	Pulic databases	10047000	10219000	7533900	5515800	7558700	7244800	5851300	6422000	8208700	7357300	8467300	7978200	6785300	9616300	9067300	11783000	9065100	12508000
N-Acetyl-L-alanine	Pulic databases	3594200	2154700	1868700	522000	1041700	451360	8525900	506430	6392900	651570	1560000	702060	850140	3404100	563890	1756300	599270	1854300
Indole-3-acetamide	Pulic databases	3688800	243130	141440	185230	134120	127950	280190	77386	213710	73408	106030	94408	68036	67241	57011	67916	54954	78888
Triethyl phosphate	Pulic databases	866510	887920	560040	472620	1039800	995690	751740	712540	1315600	948000	886980	862130	745510	702790	979970	1476300	674380	1004100
δ-Valerolactam	Pulic databases	555670	1554700	13.265	1839400	7.7621	331340	40137	251300	9.1837	95403	5.009	113530	11.251	61371	96609	1.6647	208810	3.3075
2-Pyrrolidinone	Pulic databases	315830	1714200	186660	92204	168250	832140	492660	425340	1235400	257330	173930	182500	224930	44120	202890	252610	180270	375060
Androsterone	Pulic databases	9514	3056.1	26.421	773.25	1542.4	489.68	7777.2	8156.8	9328.3	4714.7	2309.2	14653	4851.9	59287	5469.4	19189	9542	94229
Dihydrouracil	Pulic databases	50260	20094	19311	21233	11096	47177	16783	44712	16370	15101	20254	22399	26583	127160	28129	47542	34284	62191
[illegible]	Pulic databases	17618	3722.1	14875	1716.6	10687	2916	75513	1321.3	16879	795.39	7235.3	23064	17091	3062.8	7649.1	1754.6	7378	39312
N1-Acetylspermine	Pulic databases	78431	90829	91843	209780	31122	259560	331150	30105	668460	213370	17023	82495	29015	147580	31156	3.9614	23490	10826
Asp-Phe methyl ester	Pulic databases	2436700	1084300	3904800	3942400	1815200	4993800	2830100	866600	2696700	2302600	1957200	3339100	1333500	2090400	1160900	994470	935870	2018100
Pterine	Pulic databases	46652	23663	41137	500880	46429	363770	98943	64724	167730	131650	30767	65555	29467	47112	27140	39082	25707	15128
N-Acetylphenylalanine	Pulic databases	267270	284930	263990	313140	283740	329440	901350	305350	701230	306480	199360	303960	156410	274250	159570	267010	189610	255950
N-Alpha-acetyllysine	Pulic databases	97045000	119080	367340	225540	429990	357020	926050	801590	623380	1077500	462540	113300	446270	228340	381120	230480	445880	210040
Ritalinic acid	Pulic databases	11877	10223	5413.3	45092	18040	14412	9136	7005.3	5806.4	6117.5	10012	10166	6599.4	7809.1	1416.7	8094.9	5589	5592.6
Dihydroactinidiolide	Pulic databases	39444	53138	105.55	17725	61.983	33678	14991	10932	79265	10479	43.702	2673.5	99.332	8418.8	117.58	9232.3	71.569	7261
2'-Hydroxy-5'-methylacetoph	Pulic databases	2327000	28520	131190	51186	45447	71986	28944	51988	16530	48526	40379	34684	35679	33670	30090	39984	29093	39941
Spermidine	Pulic databases	223970	182400	133380	167300	116210	172640	222520	201130	125560	201490	54220	115060	52949	63918	145170	67589	116850	111530
2-(3,4-dimethoxyphenyl)etha	Pulic databases	33397000	18483000	28374000	19435000	21419000	28490000	25692000	26735000	37367000	19679000	24806000	19765000	20059000	21964000	20306000	33478000	15338000	25758000
N-Acetyl-L-Histidine	Pulic databases	8094	28935	22336	84072	10876	34474	34471	53026	34568	45334	3967	91291	9902.3	67515	10193	114540	13501	47139
Isonicotinic acid	Pulic databases	52711000	5319700	272030	11744	99897	213150	98208	175050	126600	373020	227950	3140000	337460	2673400	146190	491200	439100	6921200
Furfural	Pulic databases	2352500	2398300	1975500	1916100	2054300	2290900	1990700	2004200	1652900	1923400	2295800	2044900	1938500	1605200	2046200	1757400	1700900	1559700
L-phenylalanyl-L-proline	Pulic databases	385190	551260	394920	263130	362300	261510	540680	509250	623310	514990	294450	401070	203290	376780	287250	274290	366690	340490
3-Hydroxy-DL-kynurenine	Pulic databases	21646	7128.4	13546	3096	19163	60170	28236	2875.3	29764	10939	25110	24461	5417.5	52007	7675.2	2762.8	3702.6	10950
Methyl cinnamate	Reference standar	88736	75265	60273	79932	93092	86910	70826	89446	50932	84497	113470	73844	109020	71668	90455	85087	78997	62100
Benzylcinnamate	Reference standar	140360	121330	125250	54822	98636	50626	184870	72810	264630	59103	53801	75063	78777	50831	75795	66491	65410	67326
1,2-Dichloroethane	Reference standar	1047800000	3605500	5517000	4582600	5514200	4204900	5601100	4869900	6773200	4434300	5498900	5465800	5500300	5433100	5842800	5230700	5602800	4558800
m-Cresol	Reference standar	474200	513350	425590	409980	391300	390400	719240	496890	389080	432270	515520	429800	549740	408490	233240	418250	257200	419590
Cyclohexylamine	Reference standar	3464.2	11099	746.65	17191	178.54	1521.7	488.46	1624	605.51	1296.8	324.47	686.24	1161.9	629.31	315.05	407.86	717.5	321.12
2-Picoline	Reference standar	3968800	4987400	369010	228040	368600	211240	302620	1305100	294210	372070	300950	49126	293910	87466	300490	284680	326720	267120
Butylamine	Reference standar	93085	155240	100850	73492	81149	94170	142880	162030	201830	118120	78248	51188	77792	71904	87814	177600	85923	70758
6-Methyl-5-hepten-2-one	Reference standar	120510	180090	86602	88758	182250	297220	115860	18414	211200	262410	151880	157340	65089	178650	114190	201540	96626	131780
2,2,2-Trichloroethanol	Reference standar	7492400	5797700	6274900	5284300	6143400	4868700	5944200	3996500	6123000	5087600	4980500	3333000	5734900	5216500	6402600	3675500	5947700	3647600
Hydroxyacetone	Reference standar	987490	1426400	2068800	1358700	1638500	1101100	1317000	1093300	1430200	995080	1855700	1123400	1861800	1168400	974030	1846300	1376900	1317300
Methylcysteine	Reference standar	11831000	7550400	9603600	7035700	8510300	10900000	10266000	9780100	18327000	7369400	9495900	7419900	7578200	8443300	7883200	13205000	6064900	10450000
2-Acetylfuran	Reference standar	9900100	8554500	6228300	8184300	7573100	8017100	7371900	9005800	6705300	7982000	7198900	8679100	8856700	8538800	6756400	8687600	6685000	8550500
Benzophenone	Reference standar	79359	92101	81042	75515	74218	82198	76407	86916	80384	83904	84759	84762	89894	75572	82317	76870	79356	71131
TranexamicAcid	Reference standar	94056	69157	63807	76464	68659	99414	78921	67909	86707	65529	106700	67182	84630	58696	66144	48494	92627	80576
Triethylamine	Reference standar	481790	3136300	262890	509810	283960	845550	292090	440920	249460	341520	265870	27741	234860	81246	367620	55893	284780	36715
2,5-Dimethyl pyrazine	Reference standar	14431000	4524400	6509300	6464900	6315700	3899100	6713200	13397000	5776900	7083000	6867200	2839400	6851300	5707200	6518400	2178600	7237700	5112300
2,6-Di-tert-butyl-4-methylph	Reference standar	86676	81088	61086	82068	54793	100010	52777	98387	51164	81676	66540	105330	89082	100430	87041	72230	48769	84722
(E)-2-Octen-1-ol	Reference standar	94684	94841	308200	480460	273740	548380	669380	415490	132490	1125300	1308200	151700	692330	324820	203940	149280	218180	227290
Naproxen	Reference standar	6226.1	578.35	666.54	284.45	2062.8	1282.4	1332.9	97.874	2880.6	6736.5	2014.8	0.088891	2.4346	105.17	2.7445	0.056457	1.67	0.11373
2-Pentadecanone	Reference standar	1679100	2054200	2284000	1536500	1459200	1637100	2262700	1724100	2088000	1560400	1460100	1748300	1741100	1681700	1875800	1638000	1615100	1845700
Methyl dihydrojasmonate	Reference standar	2815800	3554000	3614600	2502800	2275500	2534700	3573500	2758500	3441100	2623600	2261900	2694000	2897900	2691000	3097500	2952900	2652500	2868300
Pyrazine	Reference standar	179190	13513	53658	34434	40529	65631	43960	18590	43311	38365	51273	31163	53285	67671	37480	10387	54393	31289
Methylisobutyrate	Reference standar	129070	102640	121080	137880	119300	128210	117240	151350	127600	143640	154530	118150	124760	133750	120760	120820	119300	135130
N-Nitrosodiethylamine	Reference standar	109830	81080	123220	101130	91686	107490	102780	114460	103890	101530	111960	110280	76994	112170	114850	85432	101230	92586
3,5-Dinitrosalicylic acid	Reference standar	2278900	2630800	2975400	967510	1944400	1543100	3677200	1377500	4723600	1584900	1344300	1967100	1826200	1510100	1857300	2061200	1564400	1195300
Barbituric acid	Reference standar	150490	80369	376470	579710	348680	585490	794570	518720	172300	1472800	1709200	144860	996090	409610	258660	176400	297760	299150
2-Aminophenol	Reference standar	65949	10857	15839	12033	27958	6570.7	25130	11071	21038	12127	34223	10808	21464	50341	16487	37049	22604	11355
DL-Leucine	Reference standar	282000000	217440000	270500000	176520000	158690000	275860000	427460000	185130000	335110000	246930000	222990000	196970000	159040000	273270000	209770000	170170000	186040000	229650000
[illegible]	Reference standar	53140	48421	49279	43297	56944	51467	47479	38474	55076	51374	37911	48795	48890	49592	63562	49318	56092	48339
PYRROLIDINE	Reference standar	1242200	975750	1006800	766940	793710	1267200	1492100	794170	1116100	1219200	1180000	888640	810320	1601100	1193000	1020400	946130	1236700
Octanal	Reference standar	147130	78963	343040	410330	277240	443430	689040	494750	148870	1111900	1546300	183410	804630	400600	213520	235230	223350	272910
2-(Methylthio)benzothiazole	Reference standar	1568600	47995	32798	67513	27718	40284	39733	17100	30061	13853	29997	17044	29203	54937	30591	15872	25446	31920
5-METHYLFURFURAL	Reference standar	2705200	2488600	2427000	2696400	2441700	2548100	2061800	2322900	2826200	2412200	2621400	2679200	2597100	2634200	2376100	2538100	2635300	2599900
N-ETHYLACETAMIDE	Reference standar	122040	63446	87631	75911	97531	80001	86466	115970	108450	76698	79040	137900	92701	191930	95606	132670	122190	89714
1-PENTADECANOL	Reference standar	2632800	2997800	3146900	2162500	2352400	2018300	4093300	2651600	5149800	1601500	1623200	2113800	2069800	2061700	2090900	2992400	1846400	2262500
2-Methyl-1-propanol	Reference standar	407630	429540	369850	370050	568310	400030	344020	348700	461600	495730	887980	583420	558730	613990	328690	406200	397850	472740
1-Aminopropan-2-ol	Reference standar	49156	66216	1659.7	75379	2290.7	23928	13707	112540	1556.1	47722	34009	44556	18516	8710.9	275910	3.4614	3404.6	5035.7
Dibutyl phthalate	Reference standar	10561000	9811000	9539600	9989900	9202800	8751200	9185600	7375000	9872600	8521700	8244400	7294300	8686200	9050200	10079000	6468600	9996000	7892800
Pulegone	Reference standar	7637400	2210.8	135700	1906900	127750	1810100	156490	1502800	201800	1548900	112040	90079	130130	81143	253050	59818	100510	54980
FURFURYL ALCOHOL	Reference standar	18573000	19129000	16538000	16766000	16331000	16528000	14938000	16226000	16189000	17479000	19776000	19620000	20964000	20135000	17114000	19945000	20219000	21469000
1-Phenylethanol	Reference standar	53602000	49774000	53290000	40551000	56155000	40505000	60273000	47203000	47107000	49374000	50423000	52221000	45594000	51734000	48723000	55694000	45610000	52231000
Nα-Acetyl-L-glutamine	Reference standar	211550	277770	161570	346500	93350	324500	401400	372990	411220	759590	181270	59104	75006	84849	101600	130360	351120	98495
Choline chloride	Reference standar	23332000	9808900	24586000	8420300	20497000	14987000	19134000	27512000	26691000	9797900	16490000	16734000	20233000	25712000	14482000	23811000	21243000	21590000
glycylphenylalanine	Reference standar	686480	538580	82429	38806	36773	71434	273340	68139	39094	45493	180310	198470	185380	199380	47345	340510	181700	75746
L-Glutamic acid	Reference standar	2052400	1312000	3579000	4158000	1818700	3217900	2214300	4707800	2026300	7189700	1468200	1819800	1871900	2098100	1234500	2102000	2375600	2299400
proline betaine	Reference standar	28355000	41981000	37854000	35498000	38515000	23506000	36640000	32821000	36777000	11533000	23803000	39877000	36027000	22960000	22724000	36503000	27592000	32305000
N-methylalanine	Reference standar	775330	545190	1044000	422390	750780	731900	940970	999010	1023400	694300	504630	616830	657310	917190	630960	872920	922370	832190
tryptophan betaine	Reference standar	267580	295300	954260	207430	706150	84977	2212600	50734	846330	59267	872090	209960	341610	82347	584530	77634	475660	55385
O-Succinyl-L-Homoserine	Reference standar	34352	4825.7	4021.7	3204	19997	3098.2	5648.6	4280.5	14169	1924.8	20925	4189	21321	16742	8814.1	17620	12125	18521
Cytidine 5'-Diphosphocholine	Reference standar	38072	29799	17502	34742	26060	18139	18187	16622	26173	16291	17029	28968	23197	26808	13687	25580	3756.1	21286
N-Methyl-L-Glutamate	Reference standar	171670	51604	140040	204670	117560	255600	200790	143230	331750	219440	177720	51646	63276	184850	65194	125590	50373	182920
N-Methyl-D-Aspartic Acid	Reference standar	49391	35355	62830	122270	80240	50439	110910	174570	18302	120440	79913	46906	89772	73788	60971	26304	67558	65819
N-Alpha-Acetyl-L-Asparagin	Reference standar	728950	256770	61906	90998	63471	92627	123560	38921	83353	24498	62179	33148	42056	28551	35283	16379	25280	65870
Phosphocholine	Reference standar	13956000	13278000	15259000	11064000	14291000	11820000	16306000	14087000	13583000	14040000	12469000	16137000	11639000	14532000	12558000	15061000	11890000	14763000
2,4-Dihydroxypteridine	Reference standar	164840	86401	111080	92498	101450	127980	124630	119890	192100	111090	124510	86895	93891	90217	111780	179500	79200	117300
L-Methionine Sulfoximine	Reference standar	34880	128120	19798	34185	25062	14339	22596	30584	21524	23067	23209	7823.1	16965	14630	14978	13158	37137	17099
2'-Deoxycytidine 5'-Diphosph	Reference standar	671600	459950	631470	575710	333620	1225700	519190	1194800	347640	668980	433730	791070	471390	635140	309560	933700	314660	853940
Lumichrome	Reference standar	10542	15258	5714.8	26716	3833.1	7792.6	5621.7	9321.5	4779.8	6477	7728.6	16755	8083.3	16904	8980.4	20060	10910	18386
L-Tryptophanamide	Reference standar	537040	583140	468380	475830	451670	504710	318400	493470	535420	826700	603360	477290	449920	1077600	652340	875550	597600	946670

Table S15 The ionization modes and ion pairs of the metabolites

Compounds	Ionization mode	Q1 (Da)	Q3 (Da)
L-Cystine	Negative	239.0	88.8
L-Threonine	Negative	118.0	73.0
L-Arginine	Negative	173.1	155.0
L-Aspartic Acid	Negative	132.0	71.9
L-Citrulline	Negative	174.1	131.9
L-Glutamic Acid	Negative	146.1	73.0
L-Isoleucine	Negative	130.1	88.1
L-Pyroglutamic Acid	Negative	128.0	82.0
L-Serine	Negative	104.0	74.4
Allantoin	Negative	157.0	157.0
Dopamine	Negative	152.0	135.1
Hexanoyl Glycine	Negative	172.1	126.1
L-Asparagine Anhydrous	Negative	131.1	113.0
L-Carnosine	Negative	225.2	132.1
L-Cysteine	Negative	120.0	84.0
L-Glutamine	Negative	145.1	73.9
L-Homocitrulline	Negative	188.1	145.9
N-Acetyl-L-Glutamic Acid	Negative	188.1	100.1
N-Acetyl-L-Leucine	Negative	172.1	128.2
N-Acetylneuraminic Acid	Negative	308.1	170.1
N-Isovalerylglycine	Negative	158.1	114.1
N-Propionylglycine	Negative	130.1	83.7
O-Phospho-L-Serine	Negative	184.1	148.0
S-Sulfo-L-Cysteine	Negative	200.2	113.0
Trans-4-Hydroxy-L-Proline	Negative	130.1	70.9
Benzoic Acid	Negative	121.0	121.0
Terephthalic Acid	Negative	165.0	93.0
4-Dimethoxyphenyl) Acetic A	Negative	195.1	136.0
2-Methoxybenzoic Acid	Negative	151.1	76.9
3,4-Dihydroxyphenylacetic Ac	Negative	167.1	123.1
3-Hydroxyanthranilic Acid	Negative	152.0	91.0
P-Hydroxyphenyl Acetic Ac	Negative	151.0	93.0
Taurocholic Acid	Negative	514.3	514.3
Taurochenodeoxycholic Aci	Negative	498.3	498.0
Glycoursodeoxycholic Acid	Negative	448.3	448.0
Glycocholic Acid	Negative	464.3	464.0
Glycochenodeoxycholic Acid	Negative	448.3	448.3
Chenodeoxycholic Acid	Negative	391.3	391.0
Phenethylamine	Negative	120.9	77.0
Dulcitol	Negative	181.1	101.0
1-Methylxanthine	Negative	165.1	79.9
2,6-Dihydroxypurine	Negative	151.0	79.9
S-Methylcysteine	Negative	134.1	81.0
Adenosine 5'-Monophosphate	Negative	346.0	96.9
Hypoxanthine	Negative	135.0	65.0
Thymidine	Negative	241.1	122.3
β-Nicotinamide Mononucleoti	Negative	333.1	211.0
[illegible]	Negative	187.0	148.1
L-Thyroxine	Negative	775.2	758.6
Pyrroloquinoline Quinone	Negative	329.0	240.9
Citric Acid	Negative	191.0	147.1
Succinic Acid	Negative	117.0	73.0
Cis-Aconitic Acid	Negative	173.0	76.9
Pyruvic Acid	Negative	87.0	59.0
Citramalic Acid	Negative	147.1	85.1
Tryptamine	negative	159.2	159.0
1,5-Anhydro-D-Glucitol	Negative	163.2	76.8
D-Trehalose	Negative	341.0	243.9
D-Erythronolactone	Negative	117.0	117.0
D-Arabinose	Negative	149.0	70.8
L-Fucose	Negative	163.0	76.8
L-Rhamnose	Negative	163.0	76.8
D-Gluconic Acid	Negative	195.1	70.9
D-Glyceric Acid	Negative	105.0	58.9
L-Gulonic-γ-Lactone	Negative	177.1	70.9
Orotic Acid	Negative	155.0	112.0
Pantothenate	Negative	218.1	88.0
3-Indolebutyric Acid	Negative	202.1	156.2
Indole-3-Carboxaldehyde	Negative	144.1	142.1
2,6-Diaminopimelic Acid	Negative	189.1	73.8
2-Aminoethanesulfinic Acid	Negative	108.0	67.5
2-Aminoethanesulfonic Acid	Negative	124.0	81.9
2-Hydroxybutanoic Acid	Negative	103.0	72.8
2-Hydroxyisocaproic Acid	Negative	131.1	72.8
2-Hydroxy-3-Methyl Butyric A	Negative	117.1	116.8
3-Hydroxybutyrate	Negative	103.1	57.0
3-Methylcrotonyl Glycine	Negative	156.1	71.9
4-Hydroxy-2-Oxoglutaric Aci	Negative	161.0	84.7
Adipic Acid	Negative	145.1	101.0
Creatine	Negative	130.1	103.8
Dodecanedioic Acid	Negative	229.2	211.0
Quinic Acid	Negative	191.0	87.0
L-3-Phenyllactic Acid	Negative	165.1	72.8
L-Lactic Acid	Negative	89.0	72.0
L-Dihydroorotic Acid	Negative	157.0	140.0
Malonic Acid	Negative	103.0	59.0
Mandelic Acid	Negative	151.0	93.0
Methylmalonic Acid	Negative	117.0	73.0
[illegible]	Negative	263.1	161.1
Phenyllactate (Pla)	Negative	165.1	72.8
Phenylpyruvic Acid	Negative	163.1	146.0
Pyrrole-2-Carboxylic Acid	Negative	110.1	67.3
Shikimic Acid	Negative	173.1	73.0
Subericacid	Negative	173.1	80.9
[illegible]	Negative	[illegible]	[illegible]
12-Hete	Negative	319.1	257.4
Lysope 22:6	Negative	568.3	153.0
Lysope [illegible]	Negative	500.3	153.0
Lysope 18:1	Negative	478.3	281.3
Lysope 18:0	Negative	480.3	283.3
Lysope 16:0	Negative	452.3	255.2
Lysope 14:0	Negative	424.2	227.2
Lysope 18:0	Negative	437.3	153.0
Lysope 16:0	Negative	409.2	153.0
9-Hpode	Negative	311.1	223.1
13S-hydroxy-9Z,11E,15Z-octad	Negative	293.2	185.3
[illegible]9,10-dihydroxy-12Z-oct	Negative	313.2	183.3
γ-Linolenic Acid(C18:3N6)	Negative	277.2	233.0
Tridecanoic Acid	Negative	213.2	195.0
Palmitoleic Acid (C16:1)	Negative	253.2	95.0
Hexadecanoic Acid (C16:0)	Negative	255.2	255.2
Linoleic Acid (C18:2N6C)	Negative	279.2	279.2
Dodecanoic Acid(C12:0)	Negative	199.2	199.2
Elaidic Acid (C18:1N9T)	Negative	281.3	262.9
8Z,11Z,14Z,17Z-eicosapenta	Negative	300.9	257.2
Z,10Z,13Z,16Z,19Z-docosahe	Negative	327.2	283.3
cis-11,14-Eicosadienoic Acid (C	Negative	307.2	289.2
11,14,17-Eicosatrienoic Acid (	Negative	305.3	149.5
Arachidic Acid(C20:0)	Negative	311.3	293.3
α-Linolenic Acid(C18:3N3)	Negative	277.2	127.0
Neopterin	Negative	252.1	147.1
[illegible]	Negative	167.1	150.1
[illegible]	Negative	145.1	80.9
N-Acetylglycine	Negative	116.0	71.8
Thiamine Triphosphate	Negative	503.0	421.0
N-Acetylthreonine	Negative	160.0	71.8
[illegible]	Negative	267.1	133.8
D-Sedoheptulose 7-Phosphate	Negative	288.0	178.0
Oxoadipic Acid	Negative	159.1	97.1
[illegible]	Negative	265.0	79.9
[illegible]	Negative	181.0	92.9
Aminomalonic Acid	Negative	118.0	100.1
3-Methyl-2-Oxobutanoic Aci	Negative	115.0	87.0
Palmitaldehyde	Negative	239.0	75.0
Allysine	Negative	144.1	108.0
[illegible]	Negative	293.0	275.0
8,15-Dihete	Negative	335.1	253.1
N-Acetylmethionine	Negative	190.1	84.0
Ribulose-5-Phosphate	Negative	229.0	97.0
Argininosuccinic acid	Negative	289.1	97.1
2-Deoxyribose 1-Phosphate	Negative	213.1	81.1
N-Acetylglucosamine 1-Phosph	Negative	300.1	96.9
Estrone	Negative	269.2	269.3
[illegible]-4-hydroxyphenyl)ethylene a	Negative	263.0	79.9
Xanthosine	Negative	283.1	108.0
estrone 3-sulfate	Negative	349.1	80.0
UDP-glucose	Negative	565.1	79.0
3'-Sialyllactose	Negative	632.2	87.0
1-Methylguanine	Negative	164.1	66.0
DL-3,4-Dihydroxyphenyl glyc	Negative	169.1	93.0
dehydrocholesterol	Negative	397.4	341.3
Indoleacrylic acid	Negative	186.1	116.0
2-Methylbenzoic acid	Negative	135.0	65.0
Hydroxyphenyllactic acid	Negative	181.1	72.8
INDOLE-3-CARBINOL	Negative	146.1	47.0
DL-o-Tyrosine	Negative	180.1	107.0
L-Erythrulose	Negative	119.0	41.0
N-Acetyl-L-phenylalanine	Negative	206.1	88.0
Cyclamic acid	Negative	178.1	80.0
D-(+)-Malic acid	Negative	133.0	43.0
[illegible]nosine 5'-monophosphate (	Negative	346.1	96.9
Tetradecanedioic acid	Negative	257.2	105.2
5-Methoxytryptophol	Negative	190.1	116.0
Uridine triphosphate (UTP)	Negative	483.0	79.0
6β-hydroxytestosterone	Negative	303.2	269.2
O-Acetyl-L-serine	Negative	146.1	59.0
Indoxylsulfuric acid	Negative	212.0	77.0
Salicyluric acid	Negative	194.1	92.0
Formononetin	Negative	267.1	195.0
Ellagic acid	Negative	301.0	97.0
N-Oleoyl Glycine	Negative	338.3	74.0
Ginkgolic acid	Negative	345.3	106.0
Hydroquinone	Negative	109.0	53.0
Anthranilic acid	Negative	136.0	65.0
Succinic anhydride	Negative	99.0	80.0
5-Methylsalicylic acid	Negative	151.0	106.0
Indoleacetaldehyde	Negative	158.1	128.1
Hexadecanedioic acid	Negative	285.2	57.0
Vanillylmandelic acid	Negative	197.1	136.0
Gamma-Glu-Leu	Negative	259.1	87.0
O-Desmethylnaproxen	Negative	215.1	143.1
Isopropyl myristate	Negative	268.8	191.1
Methyl stearate	Negative	297.1	281.0
Ethyl salicylate	Negative	164.6	75.1
Maltol	Negative	125.0	57.8
Octadecanamide	Negative	282.4	238.3
2-naphthol	Negative	143.3	101.0
1-Naphthol	Negative	142.9	115.2
Butyl Acetate	Negative	117.8	68.9
2-n-Pentylfuran	Negative	137.1	101.0
Methyl-5-nitroimidazole-1-etha	Negative	170.1	95.7
[illegible]	Negative	179.0	97.0
Sorbic acid	Negative	111.0	96.2
Methyl hexadecanoate	Negative	269.4	101.2
Phenoxyacetic acid	Negative	151.3	107.1
4-Ethylphenol	Negative	120.8	59.0
4-tert-Octylphenol	Negative	205.2	188.9
RICINOLEIC ACID	Negative	297.3	279.0
[illegible]	Negative	255.4	237.5
Cis-3-Hexenylacetate	Negative	141.1	59.1
2-(Methylthio)ethanol	Negative	90.7	59.8
4-Hydroxybenzyl alcohol	Negative	123.0	77.1
2-Nonanone	Negative	140.9	58.0
Propylparaben	Negative	179.1	136.0
2,4-Di-tert-butylphenol	Negative	205.2	189.2
Methylparaben	Negative	151.1	135.9
[illegible]hydroxy-5Z,8Z,10E,14Z,17Z-	Negative	317.2	179.2
12-hydroxy-5Z,8Z,10E,14Z-ei	Negative	319.3	179.0
5-hydroxy-6E,8Z,11Z,14Z-eico	Negative	319.2	115.0
(±)11(12)-epoxy-5Z,8Z,14Z-eic	Negative	319.3	167.2
[illegible]	Negative	295.2	195.1
[illegible]	Negative	293.0	112.8
[illegible]droxy-4Z,7Z,10Z,12E,16Z,19	Negative	343.1	205.0
[illegible]	Negative	295.2	171.2
[illegible]	Negative	293.0	185.1
auroursodeoxycholic acid sodium	Negative	498.2	123.9
Xylose	Negative	149.0	[illegible]
[illegible]	Negative	[illegible]	107.1
[illegible] (C18)	Negative	253.2	84.1
N-acetyl-betaalanine	Negative	129.9	70.9
Scyllo-inositol	Negative	178.9	104.3
TRP-GLU	Negative	332.0	468.6
N-acetylornithine	Negative	173.0	[illegible]
N-Acetyl-L-Aspartate	Negative	174.1	160.9
Dethiobiotin	Negative	213.3	58.0
Mono-Methyl Glutarate	Negative	145.0	170.0
[illegible]	Negative	167.9	74.0
D-Homocysteine	Positive	136.0	73.0
Glycine	Positive	76.0	42.0
L-Tyrosine	Positive	182.1	122.9
L-Lysine	Positive	147.0	130.1
L-Ornithine	Positive	133.0	114.8
L-Alanine	Positive	90.0	89.9
L-Histidine	Positive	156.1	114.9
L-Leucine	Positive	132.1	69.0
L-Methionine	Positive	150.0	89.9
L-Phenylalanine	Positive	166.0	93.0
L-Proline	Positive	116.1	116.0
L-Tryptophan	Positive	205.1	118.0
L-Valine	Positive	118.1	58.1
(5-L-Glutamyl)-L-Amino Aci	Positive	219.0	130.1
3-Chloro-L-Tyrosine	Positive	216.0	153.1
[illegible]-3-Methylpentane-1,5-Di	Positive	163.0	103.0
5-Hydroxy-L-Tryptophan	Positive	221.1	134.0
5-Oxoproline	Positive	130.0	70.8
Asp-Phe	Positive	281.3	175.1
Betaine	Positive	118.1	72.9
Glutathione Reduced form	Positive	308.0	179.0
L-Cystathionine	Positive	223.0	90.2
L-Dopa	Positive	198.1	107.0
L-Saccharopine	Positive	277.0	195.2
L-Theanine	Positive	175.0	84.0
Methionine Sulfoxide	Positive	166.0	102.0
N,N-Dimethylglycine	Positive	104.0	61.5
N6-Acetyl-L-Lysine	Positive	189.2	130.1
N-Acetyl-L-Tyrosine	Positive	224.1	165.1
N-Acetylmannosamine	Positive	222.1	84.0
N-Glycyl-L-Leucine	Positive	189.1	129.9
Nα-Acetyl-L-Arginine	Positive	217.1	157.1
Phenylacetyl-L-Glutamine	Positive	265.1	91.0
Phe-Phe	Positive	313.4	103.1
S-(5'-Adenosyl)-L-Homocystein	Positive	385.0	88.0
Serotonin	Positive	177.0	159.1
Urea	Positive	61.0	44.0
L-Alanyl-L-Lysine	Positive	218.1	84.4
N-Acetylhistamine	Positive	154.1	80.0
Methyleugenol	Positive	179.0	95.0
P-Coumaric Acid	Positive	165.0	91.1
Syringic Acid	Positive	199.1	95.0
4-Ethylbenzoic Acid	Positive	151.1	133.1
4-Hydroxybenzoic Acid	Positive	139.0	95.0
Phthalic Acid	Positive	167.0	93.0
[illegible]o-1-Methyl-4-Oxo-3-Pyridinec	Positive	153.1	108.0
4-Pyridoxic Acid	Positive	184.1	138.1
Theobromine	Positive	181.0	108.0
Acetylcholine Chloride	Positive	147.1	147.1
Choline	Positive	105.1	61.0
Putrescine	Positive	89.0	60.8
Diethanolamine	Positive	106.0	91.0
4-Nitrophenol	Positive	140.0	100.5
Inositol	Positive	181.1	99.0
Myoinositol	Positive	181.1	102.9
Vanillin	Positive	153.0	92.0
1,7-Dimethylxanthine	Positive	181.1	98.0
1-Methyladenine	Positive	150.0	119.0
1-Methylhistidine	Positive	170.1	109.1
3'-Adenylic Acid	Positive	348.0	136.1
5,6-Dihydro-5-Methyluracil	Positive	129.1	84.0
5-Methyluridine	Positive	259.1	110.0
7-Methylxanthine	Positive	167.0	149.1
Adenine	Positive	136.1	94.0
Cytidine-5-Monophosphate	Positive	324.0	246.2
Cytosine	Positive	112.0	68.9
Inosine	Positive	269.1	139.3
Purine	Positive	121.0	67.0
Thymine	Positive	127.0	84.0
Uracil	Positive	113.0	69.9
Uridine	Positive	245.1	117.0
3,3',5-Triiodo-L-Thyronine	Positive	651.7	508.1
Norepinephrine	Positive	170.0	135.1
L-Carnitine	Positive	162.0	102.0
D-Arabitol	Positive	153.1	69.0
D-Mannitol	Positive	183.1	83.0
D-Fructose	Positive	181.0	99.0
D-Mannose	Positive	181.0	99.0
D-Melezitose	Positive	505.2	163.0
Lactose	Positive	343.1	85.0
Maltose	Positive	343.0	85.0
N-Acetyl-D-Glucosamine	Positive	222.0	84.0
Raffinose	Positive	505.2	163.0
Gluconic Acid	Positive	197.1	154.9
L-Ascorbate	Positive	177.0	117.9
Nicotinamide	Positive	123.1	80.3
[illegible]	Positive	301.2	147.1
Nicotinic Acid	Positive	124.0	106.0
Nicotinuric Acid	Positive	181.1	135.0
Panthenol	Positive	206.1	76.1
Riboflavin	Positive	377.0	172.2
Trigonelline	Positive	138.0	92.0
3-Indolepropionic Acid	Positive	190.1	148.9
Indole-3-Acetic Acid	Positive	176.1	116.9
Methyl Indole-3-Acetate	Positive	190.1	132.2
3,4,5-Trimethoxycinnamic Ac	Positive	239.1	193.2
4-Acetamidobutyric Acid	Positive	146.1	68.9
4-Guanidinobutyric Acid	Positive	146.1	146.1
5-Aminovaleric Acid	Positive	118.2	83.0
6-Aminocaproic Acid	Positive	132.1	78.8
7-Methyluric Acid	Positive	183.0	135.9
Azelaic Acid	Positive	189.1	107.0
Chlorogenic Acid	Positive	355.1	337.2
Creatinine	Positive	114.1	86.0
Dl-2-Aminooctanoic Acid	Positive	160.1	120.2
D-Pipecolinic Acid	Positive	130.2	85.5
Guanidinoacetic Acid	Positive	118.1	72.9
Hippuric Acid	Positive	180.1	107.5
Homovanillic Acid	Positive	183.1	138.1
Hydrocinnamic Acid	Positive	151.1	133.1
Kynurenine	Positive	209.1	132.1
Kynurenic Acid	Positive	190.0	162.1
L-Homoserine	Positive	120.1	92.0
Maleic Acid	Positive	117.0	74.9
N'-Formylkynurenine	Positive	237.1	136.1
P-Aminohippuric Acid	Positive	195.1	92.0
Cinnamic Acid	Positive	149.1	77.0
Uric Acid	Positive	169.0	126.1
Lysopc 14:0	Positive	468.3	184.0
Lysopc 16:0	Positive	496.3	184.0
Lysopc 16:1	Positive	494.3	184.0
Lysopc 18:0	Positive	524.4	184.0
Lysopc 18:1	Positive	522.4	184.0
Lysopc 18:2	Positive	520.3	184.0
Lysopc 20:1	Positive	550.4	184.0
Lysopc 20:2	Positive	548.4	184.0
4-Hydroxybenzaldehyde	Positive	123.0	123.0
[illegible]cholic Acid Sodium Salt Hy	Positive	356.3	283.3
2-(Formylamino)Benzoic Aci	Positive	166.0	148.1
Acetaminophen Glucuronide	Positive	328.1	154.5
Cyclic Amp	Positive	330.0	312.1
N6-Succinyl Adenosine	Positive	384.0	162.1
3-Hydroxyhippuric Acid	Positive	196.1	113.9
2-(Dimethylamino)Guanosine	Positive	312.0	164.1
7-Methylguanine	Positive	166.0	123.0
B-Pseudouridine	Positive	245.0	101.1
Isoxanthopterin	Positive	180.0	152.1
H-Homoarg-Oh	Positive	189.0	56.1
Pantetheine	Positive	279.1	131.1
[illegible]	Positive	317.3	147.1
L-Pipecolic Acid	Positive	130.0	85.8
[illegible]Deoxy-5'-(Methylthio) Adeno	Positive	298.0	139.2
Sarcosine	Positive	90.0	59.0
3-Aminoisobutanoic Acid	Positive	104.0	61.5
Cys-Gly	Positive	179.0	58.9
Trans-Citridic Acid	Positive	175.0	116.0
[illegible] Acid Trisodium	Positive	224.3	210.4
Phosphoric Acid	Positive	98.9	80.9
Cholesterol	Positive	387.1	331.1
Punicic Acid	Positive	279.3	95.1
2-Aminoadipic Acid	Positive	162.1	97.9
Hordenine	Positive	166.1	121.3
Lysopc 17:0	Positive	510.1	184.4
6-Methylmercaptopurine	Positive	167.1	121.1
Ergothioneine	Positive	230.3	186.1
N-Formylmethionine	Positive	178.0	161.0
Methoxyindoleacetic Acid	Positive	206.1	147.5
Sn-Glycero-3-Phosphocholine	Positive	258.2	104.2
3-Hydroxykynurenine	Positive	225.1	121.0
P-Aminobenzoate	Positive	137.3	94.2
Indole	Positive	118.0	91.0
Isoquinoline	Positive	130.1	103.3
Piperidine	Positive	86.1	69.2
5-Aminolevulinate	Positive	132.1	86.1
2-Methylguanosine	Positive	298.1	166.3
Lysopc 15:0	Positive	482.3	184.1
Lysopc 18:2 (2N Isomer)	Positive	520.6	460.6
Lysopc 18:3	Positive	518.3	459.3
Glucosamine	Positive	180.0	162.0
2-Hydroxycinnamic acid	Positive	165.1	123.0
trans-3-Indoleacrylic acid	Positive	188.1	146.1
Acetyl-L-carnitine	Positive	204.1	85.0
DL-Stachydrine	Positive	144.1	84.1
L-Norleucine	Positive	132.1	132.1
DL-Carnitine	Positive	162.1	103.0
N-Acetyl-L-alanine	Positive	132.1	43.0
Indole-3-acetamide	Positive	175.1	77.0
Triethyl phosphate	Positive	183.1	81.0
PAF C-16	Positive	524.4	86.1
δ-Valerolactam	Positive	100.1	44.0
2-Pyrrolidinone	Positive	86.1	69.0
Androsterone	Positive	291.2	91.1
Farnesene	Positive	205.2	77.0
[illegible]	Positive	259.1	55.0
N1-Acetylspermine	Positive	245.2	100.1
Isobutyryl carnitine	Positive	232.1	57.0
[illegible]	Positive	205.2	77.0
gamma-Muurolene	Positive	205.2	121.1
Asp-Phe methyl ester	Positive	295.1	91.1
Pterine	Positive	164.0	92.0
N-Acetylphenylalanine	Positive	208.1	103.1
N-Alpha-acetyllysine	Positive	189.1	56.1
Ritalinic acid	Positive	220.1	56.0
Dihydroactinidiolide	Positive	181.1	107.1
2-Hydroxy-5-methylacetophen	Positive	151.1	65.0
Spermidine	Positive	146.2	84.1
Cuminaldehyde	Positive	149.1	43.1
(3,4-dimethoxyphenyl)ethanon	Positive	182.1	91.1
N-Acetyl-L-Histidine	Positive	198.1	83.1
2-Methylbutyroylcarnitine	Positive	246.2	57.1
Isonicotinic acid	Positive	124.0	53.0
Methyl jasmonate	Positive	225.1	91.1
Dimethyl fumarate	Positive	145.0	53.0
beta-Cubebene	Positive	205.2	77.0
Furfural	Positive	97.0	50.0
L-phenylalanyl-L-proline	Positive	263.1	120.1
LysoPE(16:1(9Z)/0:0)	Positive	452.3	60.1
3-Hydroxy-DL-kynurenine	Positive	225.1	121.0
Methyl cinnamate	Positive	163.4	125.2
Benzyl cinnamate	Positive	239.3	239.3
Isoamyl butyrate	Positive	159.2	115.0
Ethyl dodecanoate	Positive	229.1	104.1
Propyl propanoate	Positive	117.1	71.9
1,2-Dichloroethane	Positive	98.0	98.0
m-Cresol	Positive	109.4	81.1
Cyclohexylamine	Positive	99.9	71.8
2-Picoline	Positive	94.1	66.9
Butyl 3-methylbutanoate	Positive	159.4	98.9
Butylamine	Positive	74.1	57.0
6-Methyl-5-hepten-2-one	Positive	127.3	57.9
2,2,2-Trichloroethanol	Positive	149.2	121.0
Hydroxyacetone	Positive	75.1	57.1
Methylcysteine	Positive	136.2	72.9
2-Acetylfuran	Positive	111.2	111.2
Benzophenone	Positive	182.9	122.1
Tranexamic Acid	Positive	158.1	117.1
Triethylamine	Positive	102.0	61.1
2,5-Dimethyl pyrazine	Positive	109.3	81.9
2,6-Di-tert-butyl-4-methylphen	Positive	221.1	161.3
(E)-2-Octen-1-ol	Positive	129.0	129.0
[illegible]	Positive	231.8	170.3
2-Pentadecanone	Positive	227.3	227.3
Methyl dihydrojasmonate	Positive	222.3	181.2
Hexyl butyrate	Positive	173.3	114.0
Pyrazine	Positive	80.9	80.9
Methyl isobutyrate	Positive	102.9	102.9
N-Nitrosodiethylamine	Positive	103.2	103.2
3,5-Dinitrosalicylic acid	Positive	229.1	229.1
Barbituric acid	Positive	129.0	129.0
2-Aminophenol	Positive	110.2	110.2
[illegible]	Positive	165.2	135.2
p-Cymene	Positive	135.3	117.1
Hexyl Acetate	Positive	167.1	131.1
Terpinolene	Positive	137.1	101.0
gamma-Terpinene	Positive	137.2	101.1
alpha-Terpinene	Positive	137.2	118.9
DL-Limonene	Positive	137.1	86.0
Glycyrrhetinic acid	Positive	471.3	189.3
(+)-borneol	Positive	155.1	114.0
p-Menth-1-en-8-ol	Positive	155.2	99.1
Norambreinolide	Positive	251.3	101.4
Ethyl butyrate	Positive	116.6	55.0
2-Undecanone	Positive	171.1	55.2

10-UNDECENOIC ACID	Positive	185.0	114.2
Undecanal	Positive	171.1	83.1
2-Pentyl-3-phenyl-2-propenal	Positive	203.4	185.3
PYRROLIDINE	Positive	72.3	57.2
Octanol	Positive	128.2	97.0
Pyrene	Positive	203.3	185.1
2-(Methylthio)benzothiazole	Positive	182.2	109.2
5-METHYLFURFURAL	Positive	111.1	83.1
N-ETHYLACETAMIDE	Positive	88.3	57.1
PROPYL HEXANOATE	Positive	159.3	99.1
1-PENTADECANOL	Positive	229.1	176.3
2-Methyl-1-propanol	Positive	75.3	57.0
1-Aminopropan-2-ol	Positive	76.1	58.0
Dibutyl phthalate	Positive	279.3	170.9
Pulegone	Positive	153.1	99.2
ETHYL ISOBUTYRATE	Positive	117.0	55.0
FURFURYL ALCOHOL	Positive	99.0	55.1
1-Phenylethanol	Positive	124.0	83.2
Nα-Acetyl-L-glutamine	Positive	189.1	130.1
Choline chloride	Positive	104.1	60.1
[illegible] (10:0)	Positive	541.2	129.0
Isobutyl decanoate	Positive	229.2	256.9
glycylphenylalanine	Positive	223.2	144.1
L-Glutamic acid	Positive	148.4	22.8
proline betaine	Positive	118.2	179.2
[illegible]	Positive	229.3	109.0
N-methylalanine	Positive	104.5	177.1
ubiquinone	Positive	319.5	163.2
[illegible]	Positive	307.5	107.1
Glycerophosphorylcholine	Positive	258.4	248.2
tryptophan betaine	Positive	247.1	108.1
O-Succinyl-L-Homoserine	Positive	220.2	176.2
Cytidine 5'-Diphosphocholine	Positive	489.2	85.0
N-Methyl-L-Glutamate	Positive	162.3	336.4
N-Methyl-D-Aspartic Acid	Positive	148.2	116.2
N-Alpha-Acetyl-L-Asparagine	Positive	175.3	131.1
Phosphocholine	Positive	185.1	137.0
2,4-Dihydroxypteridine	Positive	165.3	112.9
L-Methionine Sulfoxide	Positive	181.3	119.0
2'-Deoxycytidine 5'-Diphospho	Positive	388.2	181.1
Cortisol	Positive	363.5	142.1
Lumichrome	Positive	243.2	118.0
L-Tryptophanamide	Positive	204.3	331.4
Omega-Hydroxydodecanoic Ac	Positive	217.3	505.7
Oleate	Positive	283.2	91.1
12,13-EpOME	negative	295.2	195.1
FFA(20:5)	negative	301.2	257.4
FFA(20:4)	negative	303.2	259.3
15-oxoETE	negative	317.2	113.2
(±)12-HETE	negative	319.2	179.0
PGE3	negative	349.2	269.0
PGF2α	negative	353.2	193.2
5-iso PGF2VI	negative	353.2	115.2
TXB3	negative	367.2	169.1
TXB2	negative	369.2	168.9
FFA(4:0)	negative	87.0	87.0
FFA(5:0)	negative	101.1	101.1
FFA(6:0)	negative	115.1	115.1
FFA(10:0)	negative	171.1	171.1
FFA(11:0)	negative	185.2	185.2
FFA(12:0)	negative	199.2	199.2
FFA(14:0)	negative	227.2	227.2
FFA(15:0)	negative	241.2	241.2
FFA(16:0)	negative	255.2	255.2
FFA(17:0)	negative	269.2	269.2
FFA(18:0)	negative	283.3	283.3
FFA(20:0)	negative	311.3	311.3
FFA(24:0)	negative	367.4	367.4
FFA(14:1)	negative	225.2	225.2
FFA(15:1)	negative	239.2	239.2
FFA(16:1)	negative	253.2	253.2
FFA(17:1)	negative	267.2	267.2
FFA(18:1)	negative	281.2	281.2
FFA(20:1)	negative	309.3	309.3
FFA(24:1)	negative	365.3	365.3
FFA(16:2)	negative	251.2	251.2
FFA(18:2)	negative	279.2	279.2
FFA(20:2)	negative	307.3	307.3
FFA(22:2)	negative	335.3	335.3
FFA(24:2)	negative	363.3	363.3
FFA(18:3)	negative	277.2	277.2
FFA(18:4)	negative	275.2	275.2
FFA(22:4)	negative	331.3	331.3
FFA(24:4)	negative	359.3	359.3
FFA(24:5)	negative	357.3	357.3
FFA(22:6)	negative	327.2	327.2
FFA(24:6)	negative	355.3	355.3
LPA(0:0/16:0)	negative	409.2	255.0
LPA(0:0/18:0)	negative	437.3	283.0
LPC(16:0/0:0)	negative	540.3	255.0
LPC(18:0/0:0)	negative	568.4	283.0
LPC(16:1/0:0)	negative	538.3	253.0
LPC(18:1/0:0)	negative	566.3	281.0
LPC(20:1/0:0)	negative	594.4	309.0
LPC(18:2/0:0)	negative	564.3	279.0
LPC(20:2/0:0)	negative	592.4	307.0
LPC(20:3/0:0)	negative	590.3	305.0
LPC(20:4/0:0)	negative	588.3	303.0
LPC(22:4/0:0)	negative	616.4	331.0
LPC(20:5/0:0)	negative	586.3	301.0
LPC(22:6/0:0)	negative	612.3	327.0
LPE(0:0/16:1)	negative	450.3	253.0
LPE(0:0/18:1)	negative	478.3	281.0
LPE(0:0/20:1)	negative	506.3	309.0
LPE(0:0/20:3)	negative	502.3	305.0
LPE(0:0/20:4)	negative	500.3	303.0
LPE(0:0/22:5)	negative	526.3	329.0
LPE(0:0/22:6)	negative	524.3	327.0
LPI(14:1/0:0)	negative	541.2	225.0
PA(16:0/20:1)	negative	701.5	255.0
PA(18:1/20:0)	negative	729.6	281.0
PC(16:0/16:0)	negative	778.6	255.0
PC(14:0/18:1)	negative	776.5	227.0
PC(16:0/16:1)	negative	776.5	253.0
PC(18:0/18:1)	negative	832.6	283.0
PC(16:1/18:1)	negative	802.6	253.0
PC(18:0/18:2)	negative	830.6	283.0
PC(18:1/18:1)	negative	830.6	281.0
PC(16:1/18:2)	negative	800.5	279.0
PC(16:0/18:3)	negative	800.5	277.0
PC(18:1/18:2)	negative	828.6	281.0
PC(16:0/20:3)	negative	828.6	305.0
PC(18:0/18:3)	negative	828.6	277.0
PC(14:0/20:4)	negative	798.5	303.0
PC(16:0/20:4)	negative	826.6	303.0
PC(18:0/20:4)	negative	854.6	283.0
PC(20:4/16:1)	negative	824.5	303.0
PC(18:2/18:3)	negative	824.5	279.0
PC(18:1/20:4)	negative	852.6	303.0
PC(20:5/18:0)	negative	852.6	301.0
PC(16:0/22:5)	negative	852.6	329.0
PC(18:2/20:4)	negative	850.6	303.0
PC(18:1/20:5)	negative	850.6	301.0
PE(16:1/16:0)	negative	688.5	253.0
PE(18:1/16:0)	negative	716.5	281.0
PE(18:1/18:0)	negative	744.6	281.0
PE(20:1/18:0)	negative	772.6	309.0
PE(18:1/16:1)	negative	714.5	281.0
PE(18:2/16:0)	negative	714.5	279.0
PE(18:1/18:1)	negative	742.5	281.0
PE(18:0/18:2)	negative	742.5	279.0
PE(20:2/16:0)	negative	742.5	307.0
PE(18:1/18:2)	negative	740.5	279.0
PE(16:0/20:3)	negative	740.5	305.0
PE(18:2/18:2)	negative	738.5	279.0
PE(16:0/20:4)	negative	738.5	255.0
PE(20:4/18:0)	negative	766.5	303.0
PE(16:0/20:5)	negative	736.5	301.0
PE(18:1/20:4)	negative	764.5	281.0
PE(18:0/20:5)	negative	764.5	301.0
PE(18:2/20:4)	negative	762.5	279.0
PE(22:6/16:0)	negative	762.5	327.0
PE(22:6/18:0)	negative	790.5	283.0
PE(20:4/22:2)	negative	818.6	303.0
PI(18:2/18:0)	negative	861.5	279.0
PI(18:0/20:4)	negative	885.5	283.0
PS(18:0/18:2)	negative	786.5	279.0
Free carnitine	positive	162.1	85.1
Acetyl-carnitine	positive	204.1	85.1
Propionyl-carnitine	positive	218.1	85.1
Butyryl-carnitine	positive	232.2	85.1
Isovaleryl-carnitine	positive	246.2	85.1
Hexanoyl-carnitine	positive	260.2	85.1
Hydroxyhexanoyl-carnitine	positive	276.2	85.1
Octanoyl-carnitine	positive	288.2	85.1
3-Methylglutaryl-carnitine	positive	290.2	85.1
3-hydroxyoctanoyl carnitine	positive	304.2	85.1
Decanoyl-carnitine	positive	316.2	85.1
3-Hydroxy-decanoyl- carnitin	positive	332.2	85.1
Lauroyl-carnitine	positive	344.3	85.1
Myristoyl-carnitine	positive	372.3	85.1
11-carboxyundecanoyl-carnitir	positive	374.3	85.1
3-Hydroxy-tetradecanoyl- carnit	positive	388.3	85.1
Palmitoyl-carnitine	positive	400.3	85.1
Stearoyl-carnitine	positive	428.4	85.1
Tiglyl-carnitine	positive	244.2	85.1
glutaconyl-carnitine	positive	274.1	85.1
Octenoyl-carnitine	positive	286.2	85.1
Hydroxy-cis-5-octenoyl-carnit	positive	302.2	85.1
Decenoyl-carnitine	positive	314.2	85.1
3-Hydroxy-decenoyl- carnitin	positive	330.2	85.1
Dodecenoyl-carnitine	positive	342.3	85.1
Tetradecenoyl-carnitine	positive	370.3	85.1
3-Hydroxy-tetradecenoyl- carnit	positive	386.3	85.1
Palmitoleoyl- carnitine	positive	398.3	85.1
Oleyl-carnitine	positive	426.4	85.1
Decadienoyl-carnitine	positive	312.2	85.1
Palmitodileoyl- carnitine	positive	396.3	85.1
Linoleyl-carnitine	positive	424.3	85.1
CE(14:0)	positive	614.6	369.4
CE(16:0)	positive	642.6	369.4
CE(18:0)	positive	670.7	369.4
CE(16:1)	positive	640.6	369.4
CE(18:1)	positive	668.6	369.4
CE(18:2)	positive	666.6	369.4
CE(20:2)	positive	694.7	369.4
CE(18:3)	positive	664.6	369.4
CE(20:3)	positive	692.6	369.4
CE(20:4)	positive	690.6	369.4
CE(22:4)	positive	718.7	369.4
CE(20:5)	positive	688.6	369.4
CE(22:5)	positive	716.6	369.4
CE(22:6)	positive	714.6	369.4
Cer(d18:0/18:0)	positive	568.6	266.0
Cer(d18:0/20:0)	positive	596.6	266.0
Cer(d18:0/22:0)	positive	624.6	266.0
Cer(d18:1/16:0)	positive	538.5	264.0
Cer(d18:1/18:0)	positive	566.6	264.0
Cer(d18:1/20:0)	positive	594.6	264.0
Cer(d18:1/22:0)	positive	622.6	264.0
Cer(d18:1/24:0)	positive	650.6	264.0
Cer(d18:1/16:1)	positive	536.5	264.0
Cer(d18:1/18:1)	positive	564.5	264.0
Cer(d18:1/20:1)	positive	592.6	264.0
Cer(d18:1/22:1)	positive	620.6	264.0
Cer(d18:1/24:1)	positive	648.6	264.0
Cer(m18:1/20:0)	positive	578.6	248.0
Cer(m18:1/22:0)	positive	606.6	248.0
Cer(m18:1/22:1)	positive	604.6	248.0
CerP(d18:1/12:0)	positive	562.4	264.0
CerP(d18:1/14:0)	positive	590.5	264.0
CerP(d18:1/16:0)	positive	618.5	264.0
CerP(d18:1/18:0)	positive	646.5	264.0
CerP(d18:1/16:1)	positive	616.5	264.0
CerP(d18:1/18:1)	positive	644.5	264.0
Cer(t18:0/24:0)	positive	668.7	282.0
Coenzyme Q10	positive	880.7	197.0
DG(14:0/16:0/0:0)	positive	558.5	313.5
DG(12:0/20:0/0:0)	positive	586.6	257.5
DG(16:0/16:0/0:0)	positive	586.6	313.5
DG(14:0/18:0/0:0)	positive	586.6	285.5
DG(16:0/18:0/0:0)	positive	614.6	341.5
DG(14:0/22:0/0:0)	positive	642.6	285.6
DG(18:0/18:0/0:0)	positive	642.6	341.6
DG(16:0/20:0/0:0)	positive	642.6	369.6
DG(14:0/16:1/0:0)	positive	556.5	311.5
DG(14:1/16:0/0:0)	positive	556.5	283.5
DG(14:0/18:1/0:0)	positive	584.5	339.5
DG(16:0/16:1/0:0)	positive	584.5	311.5
DG(16:1/18:0/0:0)	positive	612.6	341.5
DG(16:0/18:1/0:0)	positive	612.6	339.5
DG(16:0/20:1/0:0)	positive	640.6	313.6
DG(18:0/18:1/0:0)	positive	640.6	339.6
DG(16:1/22:0/0:0)	positive	668.6	311.6
DG(14:0/18:2/0:0)	positive	582.5	285.5
DG(16:1/18:1/0:0)	positive	610.6	339.5
DG(16:0/18:2/0:0)	positive	610.6	337.5
DG(18:1/18:1/0:0)	positive	638.6	339.5
DG(18:0/18:2/0:0)	positive	638.6	337.5
DG(18:1/20:1/0:0)	positive	666.6	339.6
DG(18:1/20:0/0:0)	positive	666.6	367.6
DG(20:0/18:2/0:0)	positive	666.6	337.6
DG(16:1/18:2/0:0)	positive	608.5	337.5
DG(16:0/18:3/0:0)	positive	608.5	335.5
DG(14:0/22:3/0:0)	positive	636.6	391.5
DG(16:1/20:2/0:0)	positive	636.6	365.5
DG(14:1/22:2/0:0)	positive	636.6	285.5
DG(18:1/18:2/0:0)	positive	636.6	337.5
DG(16:0/20:3/0:0)	positive	636.6	313.5
DG(18:1/20:2/0:0)	positive	664.6	365.6
DG(18:2/20:1/0:0)	positive	664.6	337.6
DG(16:1/18:3/0:0)	positive	606.5	335.5
DG(18:2/18:2/0:0)	positive	634.6	337.5
DG(18:1/18:3/0:0)	positive	634.6	335.5
DG(16:0/20:4/0:0)	positive	634.6	313.5
DG(18:2/20:2/0:0)	positive	662.6	365.5
DG(16:0/22:4/0:0)	positive	662.6	313.5
DG(18:1/20:3/0:0)	positive	662.6	339.5
DG(18:2/18:3/0:0)	positive	632.5	335.5
DG(16:1/20:4/0:0)	positive	632.5	311.5
DG(18:2/20:3/0:0)	positive	660.6	365.5
DG(18:1/20:4/0:0)	positive	660.6	361.5
DG(18:1/22:4/0:0)	positive	688.6	339.6
DG(18:3/18:3/0:0)	positive	630.5	335.5
DG(18:2/20:4/0:0)	positive	658.6	337.5
DG(18:1/20:5/0:0)	positive	658.6	339.5
DG(16:0/22:6/0:0)	positive	658.6	313.5
DG(18:2/22:4/0:0)	positive	686.6	389.5
DG(18:1/22:5/0:0)	positive	686.6	339.5
LPA(18:1/0:0)	positive	437.3	339.3
LPC(14:0/0:0)	positive	468.3	184.0
LPC(20:0/0:0)	positive	552.4	184.0
LPC(22:0/0:0)	positive	580.4	184.0
LPC(24:0/0:0)	positive	608.5	184.0
LPC(22:1/0:0)	positive	578.4	184.0
LPC(22:2/0:0)	positive	576.4	184.0
LPC(18:3/0:0)	positive	518.3	184.0
LPC(18:4/0:0)	positive	516.3	184.0
LPC(O-16:0/0:0)	positive	482.4	184.0
LPC(O-18:0/0:0)	positive	510.4	184.0
LPC(O-20:0/0:0)	positive	538.4	184.0
LPC(O-22:0/0:0)	positive	566.5	184.0
LPC(O-16:1/0:0)	positive	480.3	184.0
LPC(O-18:1/0:0)	positive	508.4	184.0
LPC(O-20:2/0:0)	positive	534.4	184.0
LPE(0:0/14:0)	positive	426.3	285.3
LPE(0:0/16:0)	positive	454.3	313.3
LPE(0:0/18:0)	positive	482.3	341.3
LPE(0:0/20:0)	positive	510.4	369.3
LPE(0:0/22:0)	positive	538.4	397.4
LPE(0:0/24:0)	positive	566.4	425.4
LPE(0:0/24:1)	positive	564.4	423.4
LPE(0:0/18:2)	positive	478.3	337.3
LPE(0:0/18:3)	positive	476.3	335.3
LPE(0:0/22:4)	positive	530.3	389.3
LPE(0:0/20:5)	positive	500.3	359.3
LPE(0:0/24:6)	positive	554.3	413.3
LPG(16:0/0:0)	positive	485.3	313.3
LPS(16:0/0:0)	positive	498.3	313.3
MG(16:0/0:0/0:0)	positive	348.3	257.3
MG(18:0/0:0/0:0)	positive	376.4	285.3
MG(18:1/0:0/0:0)	positive	374.3	283.3
PC(16:0/14:0)	positive	706.5	184.0
PC(16:0/22:0)	positive	818.7	184.0
PC(16:0/18:1)	positive	760.6	184.0
PC(18:0/20:1)	positive	816.6	184.0
PC(14:0/18:2)	positive	730.5	184.0
PC(12:0/22:2)	positive	758.6	184.0
PC(16:0/18:2)	positive	758.6	496.6
PC(16:0/20:2)	positive	786.6	496.6
PC(18:2/20:0)	positive	814.6	184.0
PC(14:0/18:3)	positive	728.5	184.0
PC(14:0/20:3)	positive	756.6	184.0
PC(16:1/22:2)	positive	812.6	184.0
PC(22:2/18:1)	positive	840.6	184.0
PC(18:3/14:1)	positive	726.5	184.0
PC(18:2/18:2)	positive	782.6	520.6
PC(18:3/18:1)	positive	782.6	184.0
PC(22:4/16:0)	positive	810.6	184.0
PC(18:0/22:4)	positive	838.6	184.0
PC(14:0/20:5)	positive	752.5	184.0
PC(22:4/14:1)	positive	780.6	184.0
PC(16:0/20:5)	positive	780.6	547.5
PC(18:0/22:5)	positive	836.6	184.0
PC(14:0/22:6)	positive	778.5	184.0
PC(16:0/22:6)	positive	806.6	496.6
PC(20:1/20:5)	positive	834.6	550.6
PC(18:0/22:6)	positive	834.6	524.6
PC(16:1/22:6)	positive	804.6	184.0
PC(18:1/22:6)	positive	832.6	184.0
PC(20:1/22:6)	positive	860.6	184.0
PC(18:2/22:6)	positive	830.6	184.0
PC(20:2/22:6)	positive	858.6	184.0
PC(O-16:0/14:0)	positive	692.6	184.0
PC(O-18:0/20:0)	positive	804.7	184.0
PC(O-16:0/14:1)	positive	690.5	184.0
PC(O-18:1/20:0)	positive	802.7	184.0
PC(O-16:0/14:2)	positive	688.5	184.0
PC(O-16:0/16:2)	positive	716.6	184.0
PC(O-16:0/18:2)	positive	744.6	184.0
PC(O-18:1/20:1)	positive	800.7	184.0
PC(O-20:1/20:1)	positive	828.7	184.0
PC(O-16:2/18:1)	positive	742.6	184.0
PC(O-18:2/18:1)	positive	770.6	184.0
PC(O-18:2/20:1)	positive	798.6	184.0
PC(O-20:2/20:1)	positive	826.7	184.0
PC(O-20:2/22:1)	positive	854.7	184.0
PC(O-18:2/18:2)	positive	768.6	184.0
PC(O-18:3/18:2)	positive	766.6	184.0
PC(O-18:3/20:2)	positive	794.6	184.0
PC(O-20:3/20:2)	positive	822.6	184.0
PC(O-20:2/24:3)	positive	878.7	184.0
PC(O-18:3/18:3)	positive	764.6	184.0
PC(O-18:3/20:3)	positive	792.6	184.0
PC(O-20:3/20:3)	positive	820.6	184.0
PC(O-18:3/20:4)	positive	790.6	184.0
PC(O-20:3/20:4)	positive	818.6	184.0
PC(O-20:4/20:4)	positive	816.6	184.0
PE(16:0/16:0)	positive	692.5	551.5
PE(16:0/18:0)	positive	720.6	579.5
PE(18:0/18:0)	positive	748.6	607.6
PE(16:1/18:0)	positive	718.5	577.5
PE(18:2/12:0)	positive	660.5	519.5
PE(18:2/14:0)	positive	688.5	547.5
PE(22:2/12:0)	positive	716.5	319.0
PE(16:0/18:2)	positive	716.5	575.5
PE(22:2/14:0)	positive	744.6	319.0
PE(18:3/16:0)	positive	714.5	573.5
PE(20:3/18:0)	positive	770.6	629.6
PE(18:3/16:1)	positive	712.5	571.5
PE(18:0/20:4)	positive	768.6	287.0
PE(18:0/22:4)	positive	796.6	655.6
PE(16:1/20:4)	positive	738.5	597.5
PE(20:1/20:4)	positive	794.6	653.6
PE(14:0/22:6)	positive	736.5	595.5
PE(16:0/22:6)	positive	764.5	623.5
PE(18:0/22:6)	positive	792.6	651.5
PE(22:6/20:0)	positive	820.6	679.6
PE(22:6/18:1)	positive	790.5	649.5
PE(22:6/20:1)	positive	818.6	677.6
PE(20:3/20:5)	positive	788.5	647.5
PE(P-18:0/16:0)	positive	704.6	563.6
PE(P-18:0/18:0)	positive	732.6	591.6
PE(P-18:0/20:0)	positive	760.6	619.6
PE(P-18:1/16:0)	positive	702.5	561.5
PE(P-18:1/18:0)	positive	730.6	589.6
PE(P-20:1/20:0)	positive	786.6	645.6
PE(P-18:2/16:0)	positive	700.5	559.5
PE(P-20:2/22:0)	positive	812.7	671.6
PE(P-18:2/18:1)	positive	726.5	585.5
PE(P-18:2/20:1)	positive	754.6	613.6
PE(P-20:2/20:1)	positive	782.6	641.6
PE(P-18:2/18:2)	positive	724.5	583.5
PE(P-18:2/20:2)	positive	752.6	611.6
PE(P-18:2/18:3)	positive	722.5	581.5
PE(P-18:2/20:3)	positive	750.5	609.5
PE(P-20:2/20:3)	positive	778.6	637.6
PE(P-20:2/22:3)	positive	806.6	665.6
PE(P-20:2/24:3)	positive	834.6	693.6
PE(P-18:2/20:4)	positive	748.5	607.5
PE(P-20:2/20:4)	positive	776.6	635.6
PE(P-20:2/20:5)	positive	774.5	633.5
PE(P-20:2/22:5)	positive	802.6	661.6
PE(P-20:2/20:6)	positive	772.5	631.5
PE(P-20:2/22:6)	positive	800.6	659.6
PS(22:6/16:0)	positive	808.5	623.5
SM(d18:0/12:0)	positive	649.5	184.0
SM(d18:0/14:0)	positive	677.6	184.0
SM(d18:0/18:0)	positive	733.6	184.0
SM(d18:0/24:0)	positive	817.7	184.0
SM(d18:1/12:0)	positive	647.5	184.0
SM(d18:1/14:0)	positive	675.5	184.0
SM(d18:1/16:0)	positive	703.6	184.0
SM(d18:0/18:1)	positive	731.6	184.0
SM(d18:1/18:0)	positive	731.6	184.0
SM(d18:1/20:0)	positive	759.6	184.0
SM(d18:1/22:0)	positive	787.7	184.0
SM(d18:1/24:0)	positive	815.7	184.0
SM(d18:2/14:0)	positive	673.5	184.0
SM(d18:1/16:1)	positive	701.6	184.0
SM(d18:1/18:1)	positive	729.6	184.0
SM(d18:1/20:1)	positive	757.6	184.0
SM(d18:1/22:1)	positive	785.7	184.0
SM(d18:1/24:1)	positive	813.7	184.0
SM(d18:1/26:1)	positive	841.7	184.0
SM(d18:2/16:1)	positive	699.5	184.0
SM(d18:2/18:1)	positive	727.6	184.0
SM(d18:2/20:1)	positive	755.6	184.0
SM(d18:2/22:1)	positive	783.6	184.0
SM(d18:2/24:1)	positive	811.7	184.0
SM(d18:2/26:1)	positive	839.7	184.0
TG(14:0/14:0/16:0)	positive	768.7	575.7
TG(14:0/16:0/16:0)	positive	796.7	575.7
TG(14:0/16:0/18:0)	positive	824.8	523.7
TG(16:0/16:0/18:0)	positive	852.8	579.8
TG(14:0/18:0/18:0)	positive	852.8	607.8
TG(14:0/16:0/22:0)	positive	880.8	607.8
TG(14:0/18:0/20:0)	positive	880.8	551.8
TG(14:0/20:0/20:0)	positive	908.9	579.8
TG(16:0/16:0/22:0)	positive	908.9	635.8
TG(18:0/18:0/18:0)	positive	908.9	607.8
TG(12:0/16:0/16:1)	positive	766.7	495.7
TG(12:0/14:0/18:1)	positive	766.7	467.7
TG(14:0/16:0/16:1)	positive	794.7	521.7
TG(14:0/14:0/18:1)	positive	794.7	495.7
TG(14:0/14:0/20:1)	positive	822.8	577.7
TG(14:0/16:0/18:1)	positive	822.8	523.7
TG(16:0/16:0/16:1)	positive	822.8	549.7
TG(16:0/16:0/18:1)	positive	850.8	577.8
TG(14:0/18:0/18:1)	positive	850.8	549.8
TG(14:0/18:0/20:1)	positive	878.8	551.8
TG(16:0/16:1/20:0)	positive	878.8	605.8
TG(14:0/18:1/20:0)	positive	878.8	579.8
TG(16:0/16:1/22:0)	positive	906.8	633.8
TG(18:0/18:0/18:1)	positive	906.8	607.8
TG(14:0/20:0/20:1)	positive	906.8	577.8
TG(16:0/18:0/22:1)	positive	934.9	633.8
TG(16:0/18:1/22:0)	positive	934.9	635.8
TG(12:0/12:0/18:2)	positive	736.6	439.6
TG(14:1/14:1/16:0)	positive	764.7	491.6
TG(14:1/16:0/16:1)	positive	792.7	521.7
TG(14:0/14:1/18:1)	positive	792.7	493.7
TG(14:0/14:0/18:2)	positive	792.7	495.7
TG(14:0/16:1/18:1)	positive	820.7	521.7
TG(16:0/16:1/16:1)	positive	820.7	547.7
TG(14:0/16:0/18:2)	positive	820.7	523.7
TG(14:0/18:1/18:1)	positive	848.8	549.7
TG(16:0/16:1/18:1)	positive	848.8	575.7
TG(14:0/18:0/18:2)	positive	848.8	547.7
TG(16:0/16:1/20:1)	positive	876.8	549.8
TG(14:0/18:1/20:1)	positive	876.8	577.8
TG(14:0/18:0/20:2)	positive	876.8	575.8
TG(16:0/16:1/22:1)	positive	904.8	631.8
