# Supplementary figures and images for: Plasma metabolomic and lipidomic alterations associated with COVID-19
Source: Natl Sci Rev. 2020 Apr 28;7(7):1157–68. doi: 10.1093/nsr/nwaa086 (PMC7197563; doi:10.1093/nsr/nwaa086)

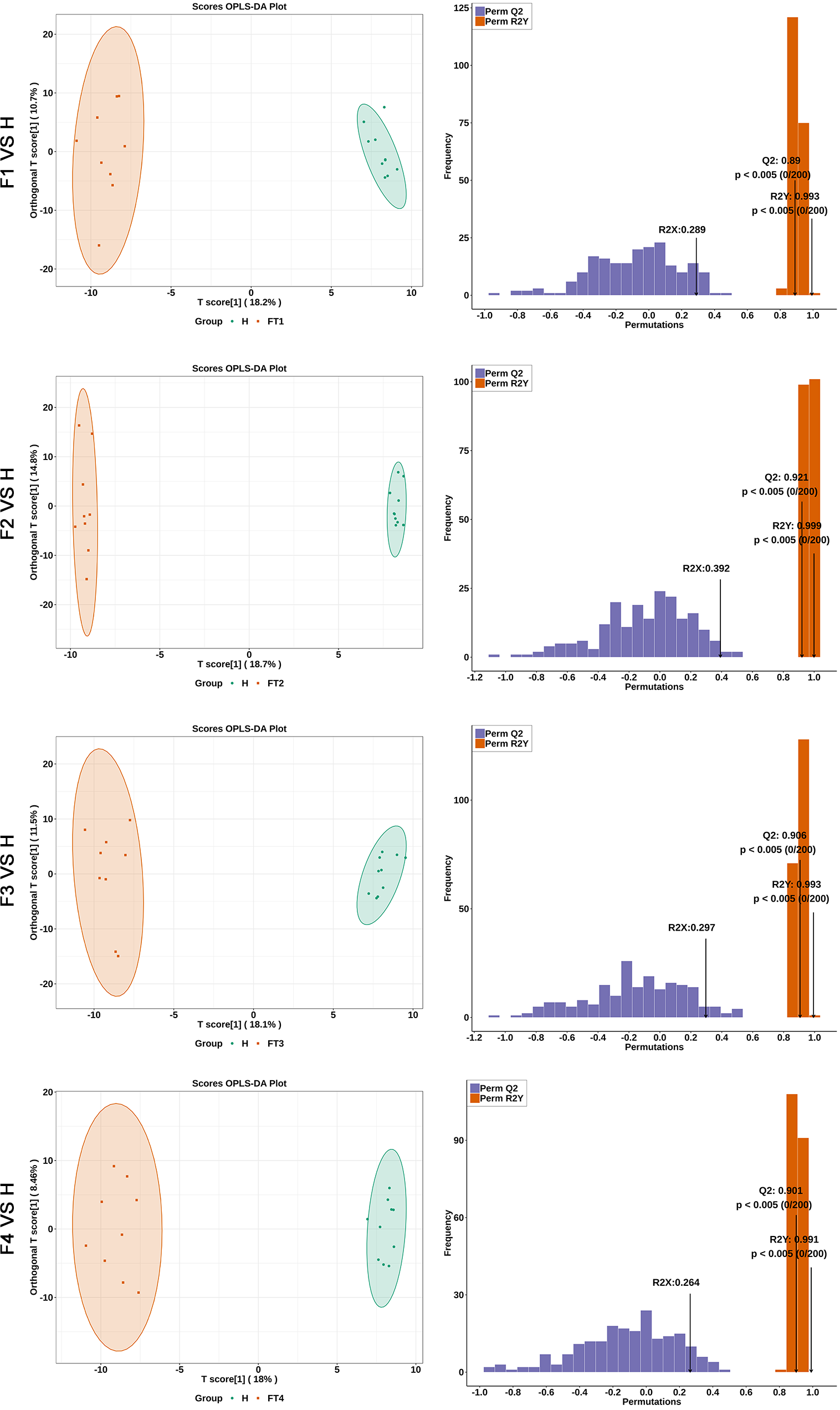

Supplement: nwaa086_Supplemental_File [file nwaa086_supplemental_file.zip › Figure S1.tif]

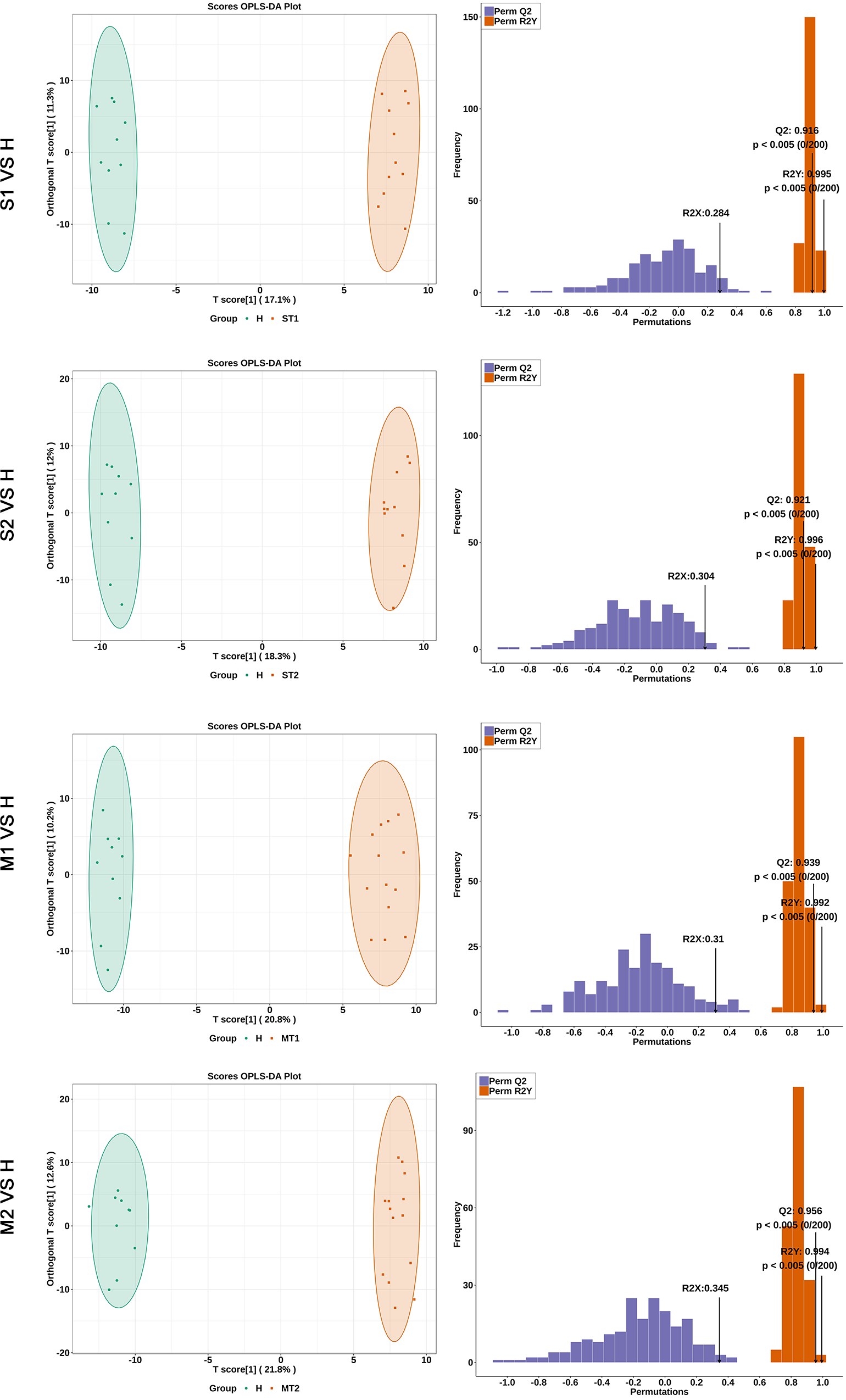

Supplement: nwaa086_Supplemental_File [file nwaa086_supplemental_file.zip › Figure S2.tif]

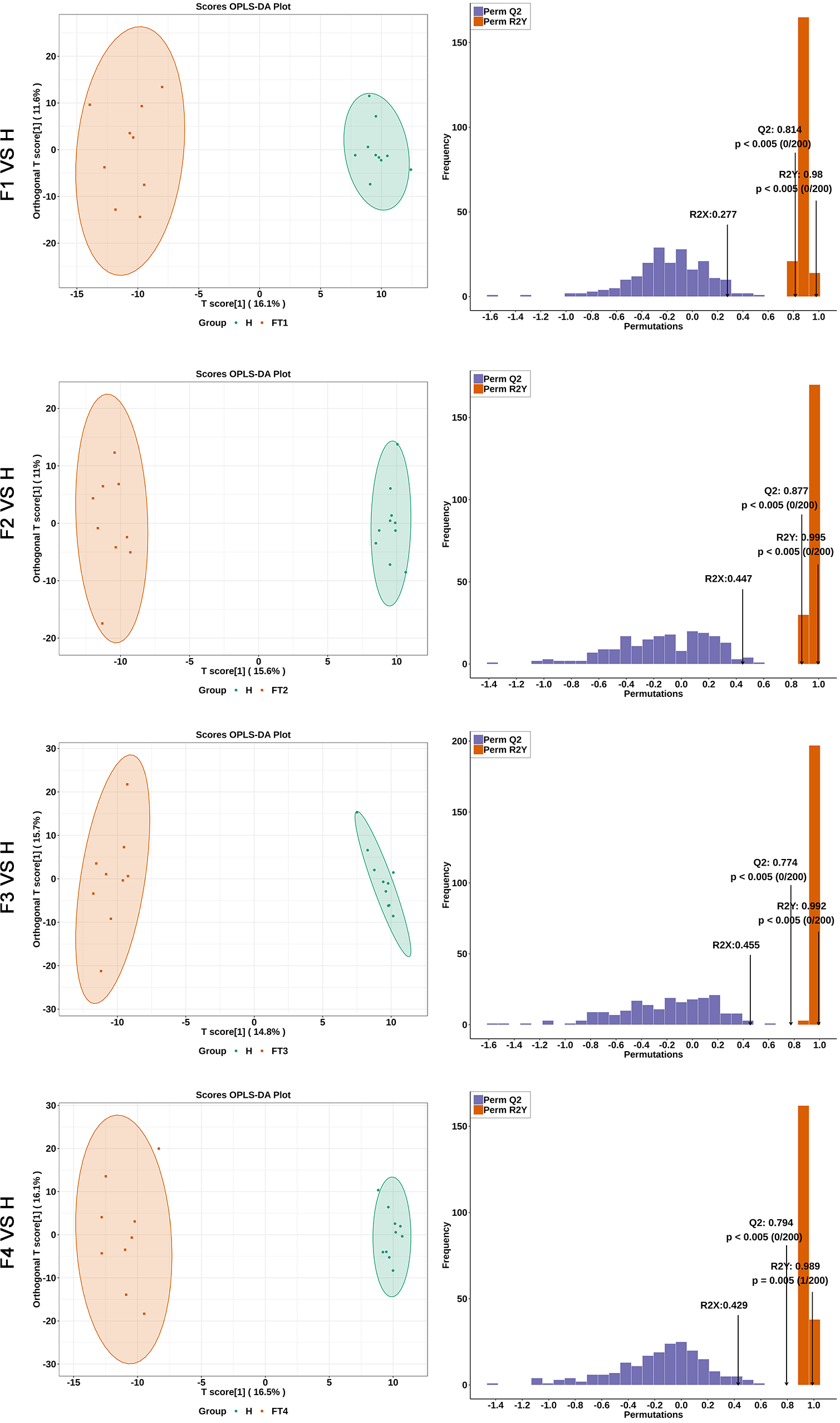

Supplement: nwaa086_Supplemental_File [file nwaa086_supplemental_file.zip › Figure S3.tif]

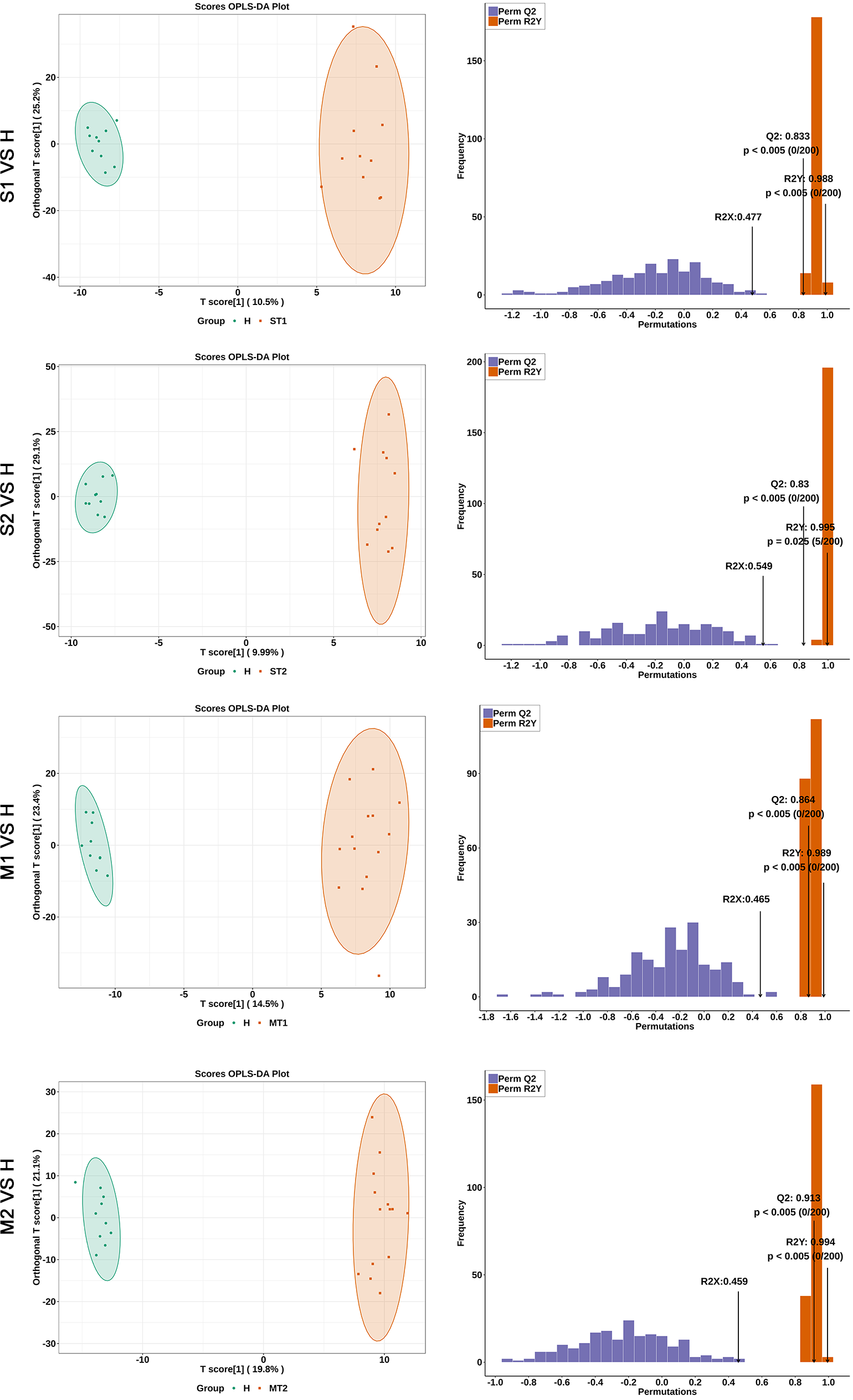

Supplement: nwaa086_Supplemental_File [file nwaa086_supplemental_file.zip › Figure S4.tif]
