# Supplementary material for: Plasma metabolomic and lipidomic alterations associated with COVID-19
Source: Natl Sci Rev. 2020 Apr 28;7(7):1157–68. doi: 10.1093/nsr/nwaa086 (PMC7197563; doi:10.1093/nsr/nwaa086)
Supplement: nwaa086_Supplemental_File [file nwaa086_supplemental_file.zip › Wu-Supplementary Data revised.doc]

**Supplementary Data**

**Supplementary methods and materials**

**Supplementary Figure S1-S4**

**Supplementary Table S1-S15**

**Methods and Materials**

Ethics and Human Subjects

All work performed in this study was approved by the Wuhan Jinyintan Hospital Ethics Committee and written informed consent was obtained from patients. Diagnosis of SARS-CoV-2 infection was based on the New Coronavirus Pneumonia Prevention and Control Program (6th edition) published by the National Health Commission of China. Healthy subjects were recruited from healthcare workers and laboratory workers at Wuhan Jinyintan Hospital and Wuhan Institute of Virology, CAS, none of whom had previously experienced SARS-CoV-2 infection.

Patient Samples

SARS-CoV-2-positive patients were enrolled in the study after diagnosis. Blood sample (≤3mL) from fatal COVID-19 patients were collected over the course of their disease at intervals of 3-5 days. Blood sample (≤3mL) from the patients with severe and mild symptoms were collected at the time when the disease were most serious (3-7 days after hospitalization) and the time before discharge. Single samples were collected from healthy volunteers recruited from healthcare workers and laboratory workers at Wuhan Jinyintan Hospital and Wuhan Institute of Virology. The throat swabs and serological testing of healthy volunteers were negative for SARS-CoV-2. All blood samples were collected after fasting overnight and by potassium-EDTA blood collection tubes. All samples used in this study are described in Table S1. All the blood samples were treated according to the biocontainment procedures of the processing of SARS-CoV-2-positive sample.

Methods for extraction of hydrophilic and hydrophobic compounds

To analysis hydrophilic compounds, sample was thawed on ice, 3 volumes of ice-cold methanol was added to 1 volume of plasma/serum, whirled the mixture for 3 min and centrifuge it with 12,000 g at 4°C for 10 min. Then the supernatant was centrifuged at 12,000 g at 4°C for 5 min, and then collected the supernatant and subjected them to LC-MS/MS analysis.

To analysis hydrophobic compounds, sample was thawed on ice, whirl around 10 s, and then centrifuge it with 3000 g at 4°C for 5 min. Take 50 μL of one sample and homogenized it with 1mL mixture (include methanol，MTBE and internal standard mixture). Whirled the mixture for 2 min. Then added 500 μL of water and whirled the mixture for 1 min, and centrifuged it with 12,000 g at 4°C for 10 min. Extracted 500 μL supernatant and concentrated it. Dissolved powder with 100 μL mobile phase B and subjected to LC-MS/MS analysis.

UPLC conditions of hydrophilic and hydrophobic compounds

The sample extracts of hydrophilic compounds were analyzed using an LC-ESI-MS/MS system (UPLC, Shim-pack UFLC SHIMADZU CBM A system, MS, QTRAP® 6500+ System). The samples were injected onto a Waters HSS T3 column (1.8 µm, 2.1 mm×100 mm). Column temperature, flow rate and injection volume were set 40°C, 0.4 mL/min and 2μL, respectively. Mobile phase was composed of water containing 0.1% formic acid (A) and acetonitrile containing 0.1% formic acid (B). The gradient program initiated from 5% B increased to 90% B in 11.0 min, and held for 1 min and then decreased 5% B for re-equilibrium.

The sample extracts of hydrophobic compounds were analyzed using an LC-ESI-MS/MS system (UPLC, Shim-pack UFLC SHIMADZU CBM A system, MS, QTRAP® 6500+ System). The samples were injected onto a Thermo C30 column (2.6 μm, 2.1 mm×100 mm). Mobile phase was composed of acetonitrile/water (60/40, v/v) containing 0.04% acetic acid and 5 mmol/L ammonium formate (A) and acetonitrile/isopropanol (10/90, v/v) containing 0.04% acetic acid and 5 mmol/L ammonium formate (B). The gradient program initiated from 20%B to 50% in 3 min, to 65% in 2 min, to 75% 4 min and to 90% in 6.5 min. The flow rate, column temperature and injection volume were set 0.35 ml/min, 45°C and 2μL, respectively. The effluent was alternatively connected to an ESI-triple quadrupole-linear ion trap (QTRAP)-MS.

ESI-Q TRAP-MS/MS of hydrophilic and hydrophobic compounds

LIT and triple quadrupole (QQQ) scans were acquired on a triple quadrupole-linear ion trap mass spectrometer (QTRAP), QTRAP® LC-MS/MS System, equipped with an ESI Turbo Ion-Spray interface, operating in positive and negative ion modes and controlled by Analyst 1.6.3 software (Sciex). The ESI source operation parameters were as follows: ion source, turbo spray; source temperature 550 °C; ion spray voltage (IS) 5500 V in positive ion mode (or -4500 V in negative ion mode); ion source gas I (GSI), gas II (GSII), curtain gas (CUR) were set at 45, 55, and 35 psi, respectively; the collision gas (CAD) was medium. Instrument tuning and mass calibration were performed with 10 and 100 μmol/L polypropylene glycol solutions in QQQ and LIT modes, respectively. QQQ scans were acquired as MRM experiments with collision gas (nitrogen) set to 5 psi. Declustering potential (DP) and collision energy (CE) for individual MRM transitions was done with further DP and CE optimization. A specific set of MRM transitions were monitored for each period according to the metabolites within this period. Each sample analysis was conducted by both positive and negative ion modes, and the MRM transitions were listed in Table S15.

Plasma mentalities and lipids data analysis

The mass spectrum data were processed by Software Analyst 1.6.3. The repeatability of metabolite extraction and detection can be judged by total ion current (TIC) and multi peak MRM. Based on home-made MWDB (metadata database) and other databases, qualitative analysis of information and secondary general data was carried out according to retention time (RT) and mass-to-charge ratio. Metabolite structure analysis referred to some existing mass spectrometry public databases, mainly including massbank (http://www.massbank.jp/), knapsack (http://kanaya.naist.jp/knapsack/), HMDB (http://www.hmdb.ca/), and Metlin (http://metlin.scripps.edu/index.php). The metabolite identification was conduced by reference standards in our home-made database and public databases, and more detailed information is listed in Table S14.

For the quality control (QC) of metabolomic analysis, we pipette 10 μL of each sample to pool a QC sample. When running sample sets on column, one QC sample was injected after 10 samples in the sequence. Metabolite quantification was accomplished by using multiple reaction monitoring (MRM) of triple quadrupole mass spectrometry. Opened the mass spectrum file under the sample machine with multiquant software to integrated and calibrated the chromatographic peaks. The peak area of each chromatographic peak represented the relative content of the corresponding substance. Finally, exported all the integral data of chromatographic peak area to save, and used the self-built software package to remove the positive and negative ions of metabolites. We calculated coefficient of variation (CV) values of the metabolites in QC samples, and removed the metabolites whose CV values were larger than 0.5. When the metabolites were detected in both positive and negative ionization modes, we removed the metabolites with larger CVs in either positive or negative mode.

To maximize identification of differences in metabolic profiles between groups, the orthogonal projection to latent structure discriminant analysis (OPLS-DA) model was applied using the MetaboAnalyst online tool (https://www.metaboanalyst.ca/). The OPLS-DA model was evaluated with the relevant R^2^ and Q^2^. And we used the permutation to assess the risk that the current OPLS-DA model is spurious.

Pathway Enrichment

We used the Kyoto Encyclopedia of Genes and Genomes (KEGG) database (http://www.genome.jp/kegg/) to analyze the KEGG pathway enrichment to identify highly enriched metabolic pathways in differential metabolites or lipids. The p-value <0.05 was considered significantly changing pathways and was used for subsequent analysis.

Statistically Processed Datasets

Plasma metabolomics and lipidomics datasets (including fold-change and *P*-values for various group comparisons) are provided in Table S4-S5 and S8-S9. Plasma metabolomics pathway enrichment are provided in Table S6-S7. Plasma lipidomic pathway enrichment are provided in Table S10-S11.

Raw Data

All raw LC-MS/MS data has been deposited to the iProX under the accession number: PXD018307.

Statistics.

The orthogonal-projection-to-latent-structure–discriminant-analysis (OPLS-DA) model was applied using R package “MetaboAnalyst”. And the OPLS-DA model verification was performed by a permutation test repeated 200 times. In general, P < 0.05 indicated the available OPLS-DA model. Student *t* test and fold change were also applied to measure the significance of each metabolite. Statistical significance was analyzed using one-tailed Student’s *t* test or Fisher's exact test, and *P* < 0.05 was considered to be statistically significant. The *P* value was corrected for multiple testing via false-discovery rate (FDR) using the Benjamini-Hochberg method.

Logistic regression analysis and receiver operating characteristic (ROC) analysis were used for diagnosis of COVID-19 patient samples and healthy subjects. ROC curves were utilized to evaluate the biomarkers performance. It was conducted applying R software version 3.6.1. (R Foundation for Statistical Computing, Vienna, Austria).

**Supplementary Figure legends**

Figure S1. The orthogonal projection to latent structure discriminant analysis (OPLS-DA) showed the best possible discrimination of metabolites between fatal COVID-19 patients and healthy people as indicated. The x-axis represents the prediction component that shows differences between groups, and the y-axis represents the orthogonal component differences within the group. R2 represents goodness of fit, Q2 represents goodness of prediction, and *P* value shows the significance level of the model (x-axis = predictive components, y-axis = orthogonal component).

Figure S2. The OPLS-DA showed the best possible discrimination of metabolites between severe or mild COVID-19 patients and healthy people as indicated. The x-axis represents the prediction component that shows differences between groups, and the y-axis represents the orthogonal component differences within the group. R2 represents goodness of fit, Q2 represents goodness of prediction, and *P* value shows the significance level of the model (x-axis = predictive components, y-axis = orthogonal component).

Figure S3. The OPLS-DA showed the best possible discrimination of lipids between fatal COVID-19 patients and healthy people as indicated. The x-axis represents the prediction component that shows differences between groups, and the y-axis represents the orthogonal component differences within the group. R2 represents goodness of fit, Q2 represents goodness of prediction, and *P* value shows the significance level of the model (x-axis = predictive components, y-axis = orthogonal component).

Figure S4. The OPLS-DA showed the best possible discrimination of lipids between severe or mild COVID-19 patients and healthy people as indicated. The x-axis represents the prediction component that shows differences between groups, and the y-axis represents the orthogonal component differences within the group. R2 represents goodness of fit, Q2 represents goodness of prediction, and *P* value shows the significance level of the model (x-axis = predictive components, y-axis = orthogonal component).
